# Deciphering the RNA recognition by Musashi-1 to design protein and RNA variants for *in vitro* and *in vivo* applications

**DOI:** 10.1093/nar/gkaf741

**Published:** 2025-08-11

**Authors:** Anna Pérez-Ràfols, Guillermo Pérez-Ropero, Linda Cerofolini, Luca Sperotto, Joel Roca-Martínez, R Anahí Higuera-Rodríguez, Pasquale Russomanno, Wolfgang Kaiser, Wim Vranken, U Helena Danielson, Alessandro Provenzani, Tommaso Martelli, Michael Sattler, Jos Buijs, Marco Fragai

**Affiliations:** Magnetic Resonance Center (CERM) and Department of Chemistry, University of Florence, and Consorzio Interuniversitario Risonanze Magnetiche di Metalloproteine (CIRMMP), Sesto Fiorentino 50019, FI, Italy; Giotto Biotech S.R.L, Sesto Fiorentino 50019, FI, Italy; Ridgeview Instruments AB, Uppsala SE 752 37, Sweden; Department of Chemistry − BMC, Uppsala University, Uppsala SE 751 23, Sweden; Magnetic Resonance Center (CERM) and Department of Chemistry, University of Florence, and Consorzio Interuniversitario Risonanze Magnetiche di Metalloproteine (CIRMMP), Sesto Fiorentino 50019, FI, Italy; Institute of Structural Biology, Molecular Targets & Therapeutics Center, Helmholtz Munich, Neuherberg 85764, Germany; Bavarian NMR Center, Department of Bioscience, TUM School of Natural Sciences, Technical University of Munich, Munich 85748, Germany; Interuniversity Institute of Bioinformatics in Brussels, VUB/ULB, Brussels 1050, Belgium; Structural biology Brussels, Vrije Universiteit Brussel, Brussels 1050, Belgium; Dynamic Biosensors GmbH, Martinsried 82152, Germany; Magnetic Resonance Center (CERM) and Department of Chemistry, University of Florence, and Consorzio Interuniversitario Risonanze Magnetiche di Metalloproteine (CIRMMP), Sesto Fiorentino 50019, FI, Italy; Dynamic Biosensors GmbH, Martinsried 82152, Germany; Interuniversity Institute of Bioinformatics in Brussels, VUB/ULB, Brussels 1050, Belgium; Structural biology Brussels, Vrije Universiteit Brussel, Brussels 1050, Belgium; AI lab, Vrije Universiteit Brussel, 1050, Brussels, Belgium; Department of Chemistry, Vrije Universiteit Brussel, Brussels 1050, Belgium; Biomedical Sciences, Vrije Universiteit Brussel, Brussels 1050, Belgium; Department of Chemistry − BMC, Uppsala University, Uppsala SE 751 23, Sweden; Science for Life Laboratory, Drug Discovery & Development Platform, Uppsala University, Uppsala SE 751 23, Sweden; Department of Cellular, Computational and Integrative Biology (CIBIO), University of Trento, Trento, 38123, Italy; Giotto Biotech S.R.L, Sesto Fiorentino 50019, FI, Italy; Institute of Structural Biology, Molecular Targets & Therapeutics Center, Helmholtz Munich, Neuherberg 85764, Germany; Bavarian NMR Center, Department of Bioscience, TUM School of Natural Sciences, Technical University of Munich, Munich 85748, Germany; Ridgeview Instruments AB, Uppsala SE 752 37, Sweden; Department of Immunology, Genetics and Pathology, Uppsala University, Uppsala SE 751 85, Sweden; Magnetic Resonance Center (CERM) and Department of Chemistry, University of Florence, and Consorzio Interuniversitario Risonanze Magnetiche di Metalloproteine (CIRMMP), Sesto Fiorentino 50019, FI, Italy

## Abstract

The Human Musashi-1 (MSI-1) is an RNA-binding protein that recognizes (G/A)U_1-3_AGU and UAG sequences in diverse RNAs through two RNA Recognition Motif (RRM) domains and regulates the fate of target RNA. Here, we have combined structural biology and computational approaches to analyse the binding of the RRM domains of human MSI-1 with single-stranded and structured RNA ligands. We have used our recently developed computational tool RRMScorer to design a set of substitutions in the MSI-1 protein and the investigated RNA strands to modulate the binding affinity and selectivity. The *in silico* predictions of the designed protein–RNA interactions are assessed by nuclear magnetic resonance and surface plasmon resonance. These experiments have also been used to study the competition of the two RRM domains of MSI-1 for the same binding site within linear and harpin RNA. Our experimental results shed light on MSI–RNA interactions, thus opening the way for the development of new biomolecules for *in vitro* and *in vivo* studies and downstream applications.

## Graphical Abstract

**Graphical Abstract:**
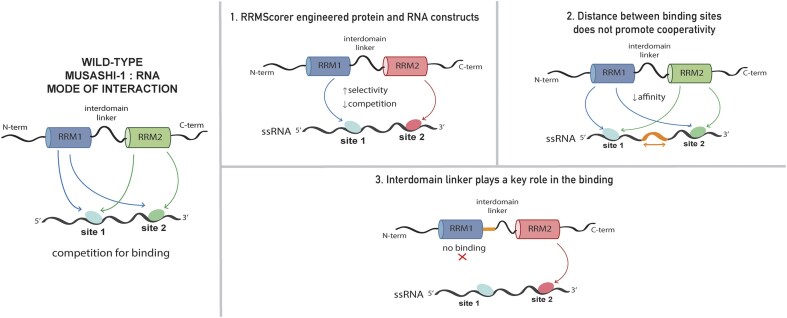

## Introduction

The fate and regulation of RNA messengers (mRNAs) are controlled by RNA-binding proteins (RBP), which recognize specific oligonucleotide sequences non-covalently. RBPs can have single or multiple domains responsible for the recognition and binding of RNA. The RNA Recognition Motif (RRM) [1, 2] is a well-studied and widespread RNA-binding domain in RBP in higher vertebrates [3]. The RRM domain comprises ∼90 residues and adopts a four-stranded antiparallel β-sheet with two α-helices packed against the β-sheet [4]. Specific amino acids present in the four β-strands and in the loops connecting the secondary structure elements are responsible for the interaction of the RRM with, usually, single-stranded RNA. Despite the high conservation of the RRM fold, subtle amino acid differences have driven the evolution of RRMs towards the binding of highly different RNA sequences. Given that the RRM fold is the most abundant RNA-binding domain, there are substantial variations in the binding specificity and interfaces of RRM folds to RNA [5], and even protein–protein interactions mediated by the helical region [6, 7].

This suggests the possibility of rationally designing RRMs toward the selective, high-affinity, binding of specific RNA motifs, which can open novel strategies for the development of bioanalytical tools for in-cell RNA visualization [8–10] or the delivery of therapeutic RNAs protected in ribonucleoprotein (RNP) complexes, similarly to Cas9 guide RNA (Cas9–gRNA) [11, 12]. The rational design of such protein variants and modified RNA sequences requires the use of computational tools based on accurate structural information and an experimental characterization of the interaction by biophysical methodologies. In this respect, we have recently developed a computational tool that estimates and scores the binding preference between RRM domains and a given RNA sequence (RRMScorer) [13].

We chose Musashi (MSI) due to its biological significance and its promising potential in the pharmaceutical field [14, 15]. Overexpression of MSI proteins has been found in several malignant tumours. Importantly, there is a proposed correlation between the protein’s expression level, the proliferative activity of cancer cells, and a poor prognosis [16, 17]. There are two members of the mammalian MSI family: Musashi-1 (MSI-1) and Musashi-2 (MSI-2), which share a 69% sequence identity. MSI-1 contains an N-terminal disordered region followed by two tandem RRMs connected by a short inter-domain linker, ∼10 amino acids long, and a large, disordered tail at the C-terminus. Both RRMs are involved in interactions with RNA, while the C-terminal region is known to bind poly(A)-binding proteins [17] and has been associated with protein aggregation [18] and the formation of cellular condensates [19].

Human MSI-1 is gradually down-regulated during neural differentiation and is involved in maintaining the undifferentiated state of neural stem cells through post-transcriptional control of downstream genes [20, 21]. Once activated, it regulates hundreds of different 3′-UTR regions of mRNAs and therefore it is implicated in various signalling pathways, including Notch and Wnt. Due to its regulatory functions, any alteration of the level of expression of MSI-1 often leads to a disruption of signalling pathways, leading to several diseases, including cancer [4]. This highlights the importance of MSI-1 and remarks on its potential to be a marker and a promising therapeutic target in cancer disease [2, 22, 23].

MSI-1, like many other RBPs, has been considered for many years as an “undruggable” target due to the lack of a well-defined binding pocket. In recent years, several computational and experimental approaches have been developed to try to find small molecule inhibitors for proteins like MSI-1. These approaches focus on trying to disrupt the RNA–protein interaction by blocking the binding interface, altering the protein structure, affecting its dynamics, varying its affinities [15, 24], or degrading the upregulated protein [25]. All these efforts, however, require a good understanding of the RNA–protein binding interface and mode of interaction. In the case of MSI-1 protein, structural information of mouse MSI-1 in complex with several short single-stranded RNA fragments [26, 27] along with *in vitro* SELEX experiments [28] on the same protein have provided insight into the RNA-binding specificity of each RRM domain of the protein. A consensus sequence (G/A)U_1-3_AGU has been reported to have a high affinity for the mouse MSI-1 RRM1, while a preference for the generic UAG motif was observed for MSI-1 RRM2 [26]. However, there is no reported information for the human MSI-1, and more importantly, there is a lack of information on how the two RRMs and the interdomain linker interplay with RNAs of different lengths, compositions, and structures. Understanding the mode of binding of the tandem domain protein with different RNAs could give valuable insights into the interaction and the binding interfaces implicated and could provide important structural information for the design of inhibitors.

In this study, we have characterized the interaction between MSI-1, a tandem RRM protein, and a set of RNA ligands to better understand the structural basis for modulating the affinity and selectivity of these interactions. This experimental data can provide valuable insights that can facilitate the redesign and optimization of both proteins and RNAs.

**Figure 1.**
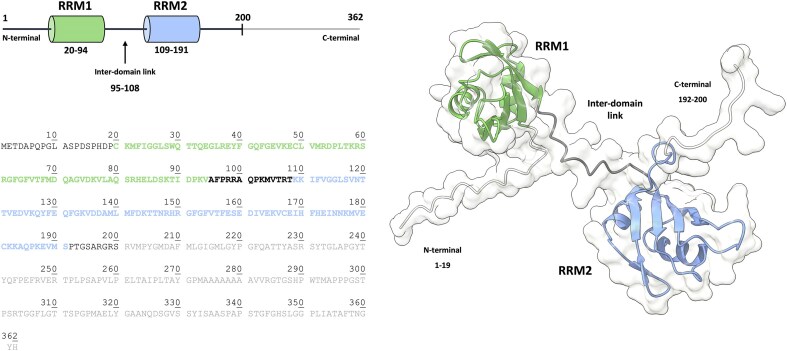
Schematic representation, sequence, and Alpha Fold model of human Musashi-1 RRM_1-2_ (1-200). Highlighted in green, black, and blue the RRM1, inter domain linker, and RRM2, respectively.

Here, we have expressed in *Escherichia coli* the two isolated domains (RRM1 and RRM2), and the tandem domain (RRM_1-2_) of human MSI-1, lacking the C-terminal tail (Fig. 1). We have designed protein substitutions and modified RNA ligand sequences using RRMScorer to modulate affinity, selectivity, and further investigate the binding mechanism between the protein and RNA. Specifically, we have employed complementary biophysical techniques to dissect and investigate the contributions to the interaction between the different constructs of the protein and selected RNA strands. For this, we have combined nuclear magnetic resonance (NMR) spectroscopy, surface plasmon resonance (SPR), fluorescence quenching assays, and size-exclusion chromatography with multi-angle light scattering (SEC-MALS) to obtain comprehensive information on the binding sites, stoichiometry, binding kinetics, and affinity of the interactions. Our results reveal a competition between the two RRMs present in the tandem domain RRM_1-2_ when recognizing the same RNA sequence, leading to a more complex and dynamic interaction than expected. In this regard, the protein variants and the RNA constructs designed by RRMScorer allowed us to investigate the contribution to the binding of the residues located in specific positions of the RRM domains, as well as that of nucleotides within the RNA strands.

## Materials and methods

### RNA strands

Synthetic single-stranded (L) and hairpin (HP) RNA (oligo-L2, oligo-L3, oligo-L3.2, oligo-L3_2C, oligo-HP4, oligo-HP4.2, oligo-HP4_2C, and oligo-HP5) for NMR experiments were purchased from Metabion International AG and Integrated DNA Technologies (IDT) (Schematics 1). Biotinylated RNA oligonucleotides (oligo-L1, oligo-L2, oligo-L2.1, oligo-L3, oligo-L3.2, oligo-HP4, and oligo-HP5, Schematics 1) used for SPR kinetic experiments were purchased from Metabion, Planegg, Germany. RNA used for fluorescence quenching assays (oligo-HP4 and oligo-HP5 constructs) conjugated with a 6-Carboxyfluorescein (6-FAM) fluorophore at the 5′-end and a BHQ-1 at the 3′-end were purchased from Integrated DNA Technologies (IDT).

**Schematics 1.**
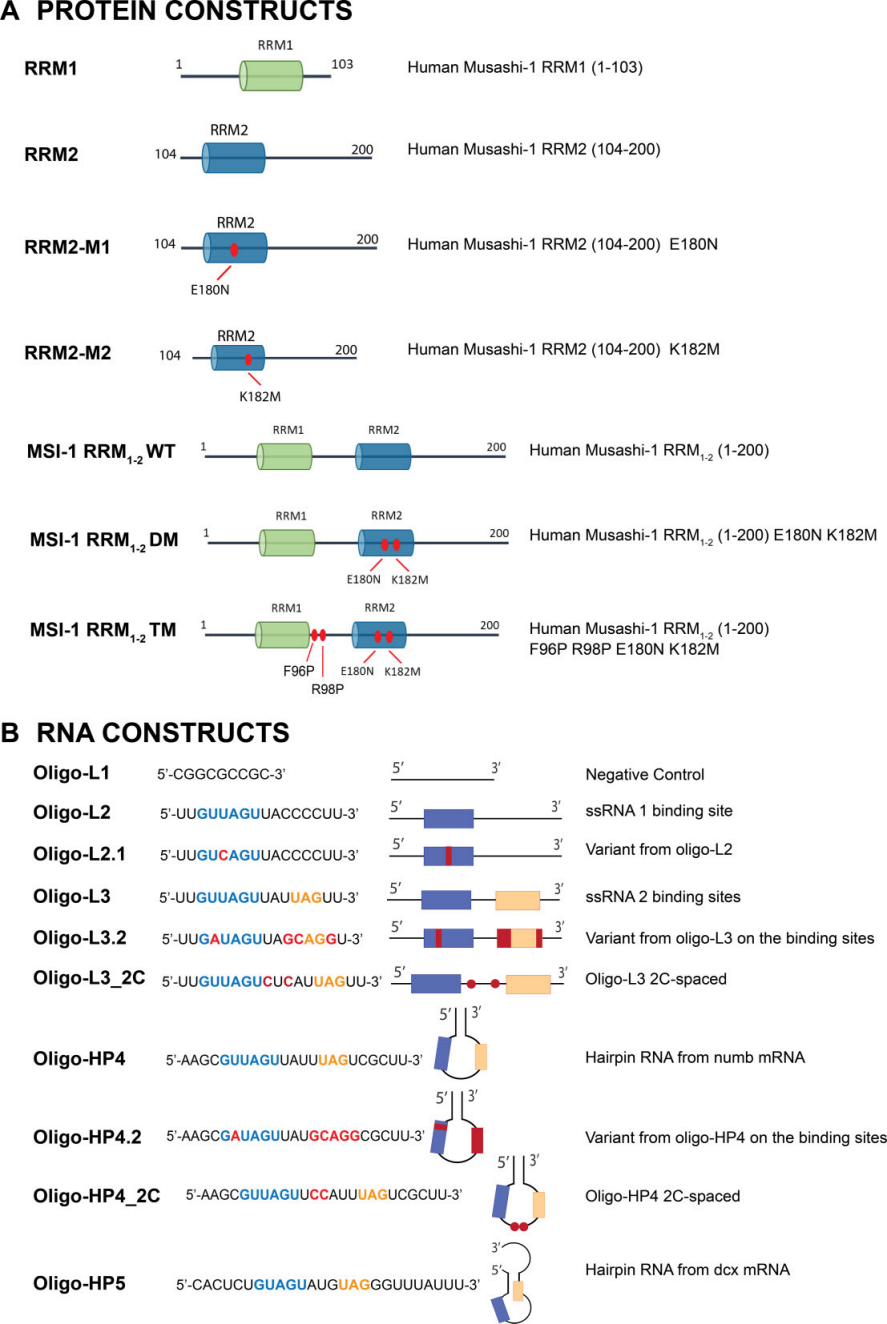
Description of (**A**) protein constructs and (**B**) RNAs used in this study. Highlighted the binding region for each RRM on the RNA sequences.

### Expression and purification of recombinant human Musashi-1 and its variants

Recombinant human MSI-1 proteins and the following variants [RRM1 (1-103), RRM2 (104–200), RRM2 E180N (RRM2-M1, hereafter), RRM2 K182M (RRM2-M2, hereafter), MSI-1 RRM_1-2_ (1-200), MSI-1 RRM_1-2_ (1-200) E180N K182M (referred as “double-mutant” MSI-1 RRM_1-2_ DM, hereafter), and MSI-1 RRM_1-2_ 1–200 F96P R98P E180N K182M (referred as “tetra-mutant” MSI-1 RRM_1-2_ TM, hereafter)] (Schematics 1) were overexpressed in BL21(DE3) *E. coli* cells. Cells were grown in LB or M9 minimal media supplemented with ^15^NH_4_Cl or ^15^NH_4_Cl and ^13^C-glucose at 37 °C until optical density (OD_600_) reached 0.6–0.8. Expression was induced with 0.5 mM of isopropyl β-d-thiogalactoside (IPTG), cells were incubated at 37°C for 3 h and harvested by centrifugation at 4°C, for 15 min at 7500 rpm. Expression and purification steps for each construct are described in detail in the Supplementary Methods.

### NMR measurements and protein assignment

Experiments for the backbone resonance assignment (3D ^1^H–^15^N–^13^C HNCA, HNCACB, and HNCO) were performed on ^13^C, ^15^N isotopically enriched samples of MSI-1 RRM_1-2_ domain at the protein [29] concentration of 300 μM in buffer solution [20 mM MES pH 6.0, 100 mM NaCl, 1 mM dithiothreitol (DTT), and 1 mM protease inhibitors]. NMR spectra were recorded at 298 K on a Bruker AvanceNEO NMR spectrometer operating at 1.2 GHz (^1^H Larmor frequency) and equipped with a TCI 3 mm cryo-probe.

Spectra were processed with the Bruker TOPSPIN software packages and analyzed with CARA (Computer Aided Resonance Assignment, ETH Zurich). The backbone resonance assignment of MSI-1 RRM_1-2_ was obtained by comparing the assignments available in the literature for the individual domains from the mouse MSI-1 protein (BMRB codes: 11450 and 36058) [26, 27] with the NMR spectra recorded on MSI-1 RRM_1-2_ and analyzing triple resonance spectra recorded on MSI-1 RRM_1-2_.

### *R*_1_, *R*_2_, and NOE measurements

For the characterization of human MSI-1 RRM_1-2_ tandem domain protein, *R*_1_, R_2_, and NOE measurements have been performed on the ^15^N- enriched sample and can be found in the Supplementary Methods [29].

### Computational design of residue substitutions in protein and RNA

For the design of protein variants and new RNA constructs, scoring of RRM–RNA interactions upon residue substitutions in both MSI-1 and RNA targets was computed with the RRMScorer method [13] (https://bio2byte.be/rrmscorer/). RRMScorer estimates the binding preferences between any specific RRM and a given RNA solely based on their sequences. In summary, from all available structures in PDB for the RRM–RNA complexes, a set of entries describing the canonical binding mode of RRM domains,^1^ was selected after a careful alignment of the RRM–RNA complexes. The contacts observed within this set of structures, and the positions of the residues involved in the binding on the RRM domains, were integrated in a probabilistic framework to extract propensities for residue-nucleotide contact preferences in specific positions. This method appeared to be particularly suitable for the limited amount of data and residue-level information that is currently available. The most relevant positions of the protein, regarding RNA binding, are short-listed based on RRMScorer database contacts analysis, and for each of them, a preference matrix has been derived showing the binding preferences to a nucleotide for any residue in that specific position.

### Titration of Musashi with RNA strands

The effect of linear (L) single stranded RNA constructs oligo-L2, oligo-L3, and oligo-L3.2 (Schematics 1) was evaluated on ^15^N-isotopically enriched MSI-1 RRM_1-2_, MSI-1 RRM1, and MSI-1 RRM2 proteins at the concentration of 100 μM in the following experimental conditions: 50 mM Tris–HCl, 140 mM NaCl, 1 mM EDTA, and 1 mM protease Inhibitors. The interaction with three other RNA strands oligo-HP4, oligo-HP4.2, and oligo-HP5 (Schematics 1) able to form a hairpin (HP) was also investigated under the same experimental conditions. The pH was 7.2 in the case of MSI-1 RRM_1-2_ and MSI-1 RRM2, while the pH was 7.5 in the case of MSI-1 RRM1, taking into account its lower isoelectric point. The interaction of MSI-1 RRM_1-2_ tandem domain with oligo-HP4_2C, an RNA strand that has a longer spacer between RRM-binding sites, was also evaluated. The effect of RNA constructs (oligo-L2, oligo-L3, oligo-L3.2, oligo-L3_2C, oligo-HP4, oligo-HP4.2, and oligo-HP4_2C) (Schematics 1) was also evaluated on ^15^N-isotopically enriched MSI-1 RRM_1-2_ DM protein at the concentration of 100 μM in the following experimental conditions: 50 mM Tris–HCl, 140 mM NaCl, 1 mM EDTA, and 1 mM protease inhibitors. 2D ^1^H ^15^N HSQC and 2D ^1^H ^15^N TROSY NMR spectra were recorded at 298 K on the single domains and on the tandem domain, respectively, using a Bruker AvanceNEO NMR spectrometer, operating at 900 MHz (^1^H Larmor frequency). In case of MSI-1 RRM_1-2_ TM protein, the interaction with oligo-L3 and oligo-HP4_2C was also investigated by using the same experimental conditions. During the NMR titrations, increasing amounts of the RNA strands were added to the protein solution to reach the final concentrations of 6, 12, 24, 50, 120, and 200 μM of RNA. Spectra were processed with the Bruker TOPSPIN software packages and analysed with CARA (ETH Zurich).

### Size-exclusion chromatography with multi-angle light scattering

Samples of 100 μL were loaded at 0.6 mL/min on a Superdex 200 10/300 GL analytical size-exclusion column (GE Healthcare), and elution was monitored by the following in-line detectors: a light scattering diode array (DAWN EOS, Wyatt Technology UK Ltd.), a dynamic module (WYATT QELS, Wyatt Technology UK Ltd.), UV detector (Smartline UV Detector 2500, Knauer) and a differential refractive index detector (Optilab rEX,Wyatt Technology UK Ltd.).

Chromatograms were analyzed using the ASTRA software (v7.3.2.19, Wyatt Technology UK Ltd.), and the interaction chromatograms were analyzed using the Protein Conjugate template.

Parameters of the specific refractive index increment d*n*/d*c* (mL/g) and UV Extinction coefficient [mL/(mg·cm)] of each domain (RRM1 and RRM2) and of the modifiers (oligo-L2, oligo-L3, oligo-HP4, and oligo-HP5) are found in Supplementary Table S1.

### MSI-1–RNA interaction kinetics experiments

Kinetic experiments were performed using surface plasmon resonance (SPR) based biosensor Biacore 3000 and T 200 (Cytiva). Data analysis was performed using TraceDrawer 1.9.2 and 1.10 (Ridgeview Instruments). A more complete experimental methodology and data analysis are described in the Supplementary Material.

### Fluorescence quenching assays

The RNA oligo-HP4 and oligo-HP5 constructs conjugated with a 6-FAM fluorophore at the 5′-end and a Black Hole Quencher 1 (BHQ1) [18] at the 3′-end were purchased from Integrated DNA Technologies (IDT). The oligos were resuspended with NMR buffer (50 mM Tris pH 7.2, 140 mM NaCl, and 1 mM EDTA) and snap-cooled by heating at 95°C for 5 min followed by incubation in ice for 15 min. All samples were diluted to a final RNA concentration of 400 nM and MSI-1 RRM_1-2_ was added to reach the desired molar ratio. A denaturing RNA control for both oligo-HP4 and oligo-HP5 were prepared by adding 8 M urea and heating the sample at 95°C before the measurement. FAM fluorophore was excited at 480 nm and emission recorded at 520 nm. The standard deviation was determined performing three replicates. Formation and unfolding of the RNA hairpins upon MSI-1 RRM_1-2_ titration was monitored based on the fluorescence emission intensity compared to a denaturing control and an RNA-only control.

### NMR spectroscopy for quenching assays

NMR samples were prepared from stock RNA samples after snap-cooling by heating at 95°C for 5 min and incubation on ice for 15 min. 1D ^1^H experiments were collected on samples of RNA at the concentration of 50 μM in NMR buffer (50 mM Tris pH 7.2, 140 mM NaCl, and 1 mM EDTA) with 10% D_2_O, added as lock signal, and acquired at 298 K on 800 MHz Bruker Avance NMR spectrometer, equipped with cryogenic triple resonance gradient probe. NMR spectra were processed with TopSpin 3.5.

## Results

### NMR characterization of free MSI-1

**Figure 2.**
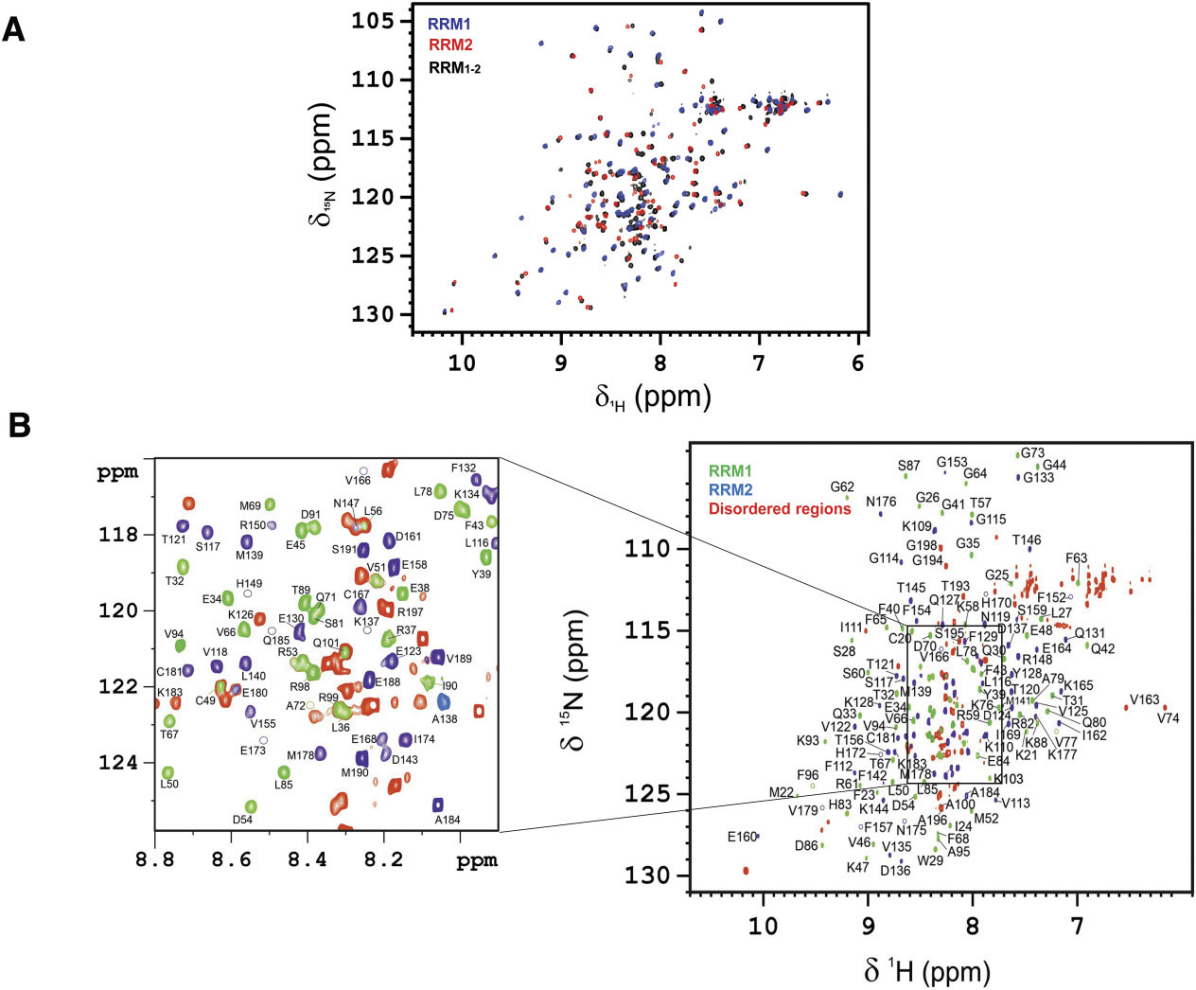
(**A**) Superimposed 2D ^1^H–^15^N HSQC spectra of isolated RRM1 (in blue) and RRM2 (in red), and TROSY spectrum of MSI-1 RRM_1-2_ tandem domain (in black). (**B**) Assignment in a 2D ^1^H–^15^N HSQC of the ^13^C, ^15^N isotopically enriched MSI-1 RRM_1-2_ domain at 900 MHz and 298 K. Highlighted in green, blue, and red the RRM1, RRM2, and disordered regions, respectively.

**Figure 3.**
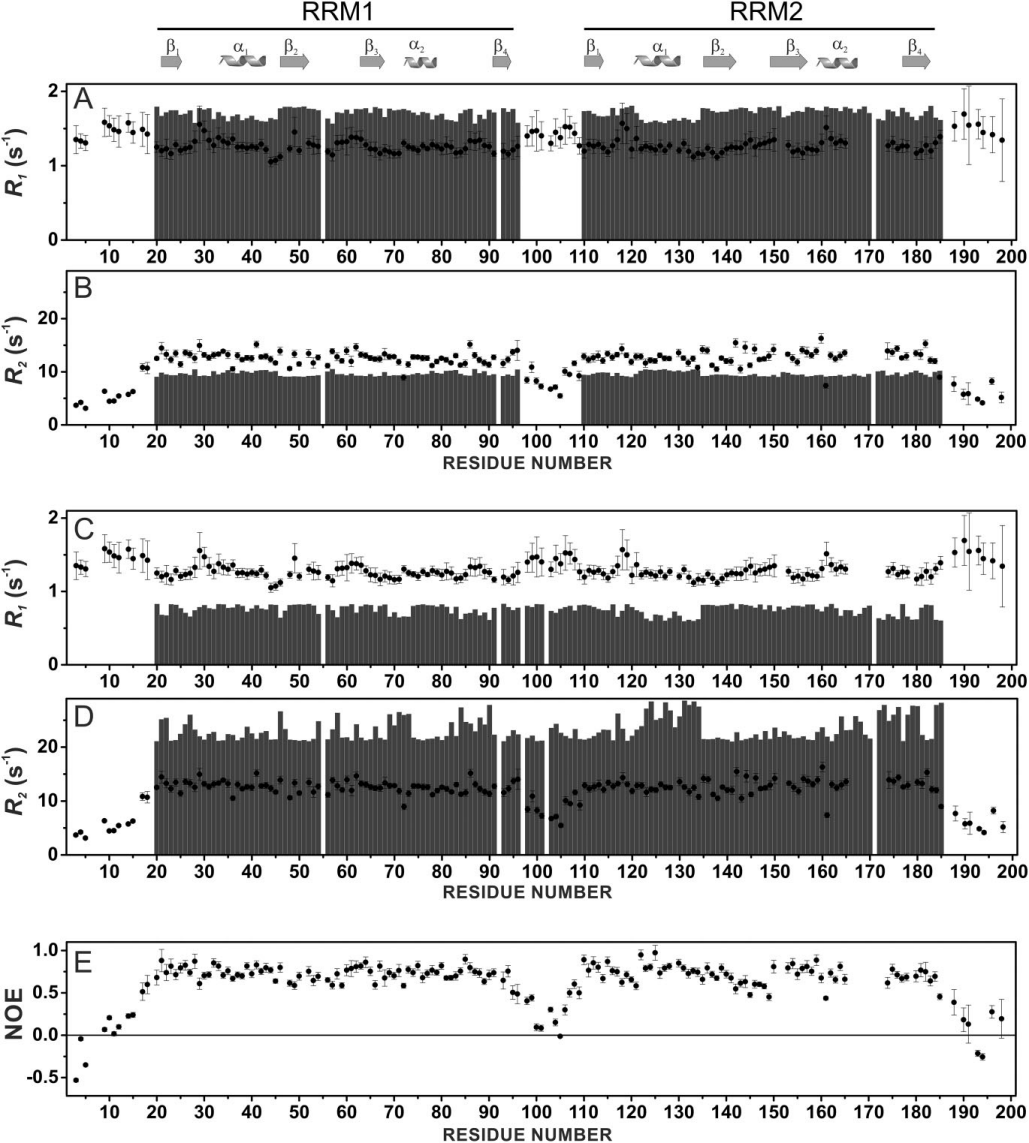
Comparison of experimental backbone ^15^N_H_*R*_1_ values for MSI-1 RRM_1-2_ construct (data collected at 298 K, black filled circles) with the calculated values (gray bars) for isolated RRM1 and RRM2 domains (**A**) and for the full MSI-1 RRM_1-2_ tandem domain construct (**C**). Comparison of experimental backbone ^15^N_H_ R_2_ values for MSI-1 RRM_1-2_ construct (data collected at 298 K, black filled circles) with the calculated values (gray bars) for isolated RRM1 and RRM2 domains (**B**) and for the full MSI-1 RRM_1-2_ tandem domain construct (**D**). Experimental NOE values for MSI-1 RRM_1-2_ construct (data collected at 298 K) (**E**).

A set of 2D ^1^H-^15^N -HSQC spectra of the three constructs (MSI-1 RRM_1-2_ tandem domain, RRM1, and RRM2 isolated domains) were recorded and superimposed to evaluate the folding state and interdomain flexibility of the tandem RRMs of human MSI-1 (see Fig. 2A). All spectra show well-dispersed signals in agreement with folded protein structures. The NMR spectrum of the MSI-1 RRM_1-2_ tandem domain is largely superimposable to the spectra of the isolated domains, as the majority of signals in the spectrum of MSI-1 RRM_1-2_ overlap with either RRM1 or RRM2 signals. The absence of large chemical shift perturbations (CSPs) when comparing the isolated domains with the tandem construct suggests a large interdomain flexibility with few interactions between the two domains or with the linker in the tandem domain construct. In the MSI-1 RRM_1-2_ spectrum, several additional signals that can be attributed to the portion of the interdomain linker are also visible. NMR ^15^N relaxation data corroborate these findings by showing the presence of a sizable interdomain flexibility (Fig. 3), as indicated by the comparison of the experimental data with the theoretical estimates of *R*_1_ and *R*_2_ values calculated by HydroNMR [30] starting from the X-ray structures (see also Supplementary Results S1). The backbone assignment of the MSI-1 RRM_1-2_ tandem domain was obtained starting from the published assignments of the murine isolated RRM domains (BMRB codes: 11450 and 36058) [26, 27], considering the high sequence homology between the mouse and human MSI-1 proteins (99.44%). This was confirmed and complemented by the analysis of triple-resonance spectra. 92.5% of all residues, including those forming the linker region, have been assigned (Fig. 2B), and the protein resonance assignment has been reported in the Biological Magnetic Resonance Data Bank (BMRB) under the accession code: 52590.

### RRM domains compete for the recognition of the (G/A)U_1-3_AGU motif

While RNA-binding studies have been conducted and binding motifs for the RRMs have been reported [17, 20, 26, 27], the binding mechanism of the human MSI-1 is still largely unexplored. Considering the very high sequence homology with the better-characterized mouse protein, it is likely that the minimal RNA sequence recognized by RRM1 and RRM2 domains is UAG. In addition, an *in vitro* selection for high-affinity RNA ligands for MSI-1 identified a longer consensus recognition sequence (G/A)U_1-3_AGU for RRM1 [26–28]. Therefore, a linear (L) RNA oligonucleotide (oligo-L2: 5′-UUGUUAGUUACCCCUU-3′) bearing a single consensus binding motif (G/A)U_1-3_AGU has been designed to explore the binding mechanism of MSI-1.

**Figure 4.**
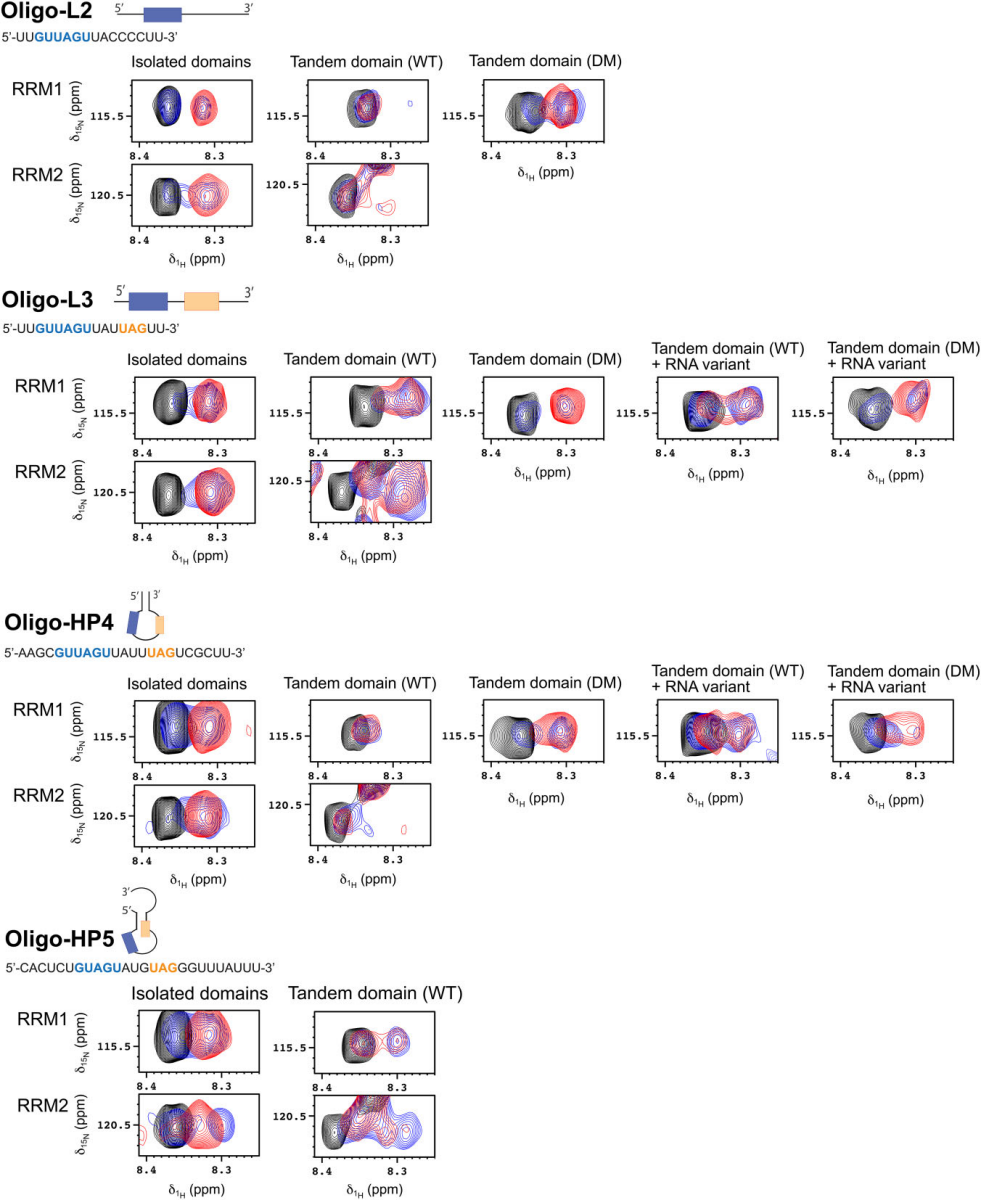
Zoom views of the 2D ^1^H–^15^N HSQC and TROSY spectra, recorded on the isolated RRM1 and RRM2 domains, on the MSI-1 RRM_1-2_ tandem domain wild-type (WT) and double mutant (DM: E180N, K182M), respectively. In black are the spectra of the free proteins, in blue the spectra of the proteins in the presence of sub-stoichiometric concentrations of oligo-L2, oligo-L3, oligo-L3.2, oligo-HP4, oligo-HP4.2, or oligo-HP5 (protein/RNA ratio of ∼1:0.5), and in red the spectra of the proteins in the presence of oligo-L2, oligo-L3, oligo-L3.2, oligo-HP4, oligo-HP4.2, or oligo-HP5 in the protein/RNA ratio of 1:1. The signals assigned to Asp-70 and Glu-130 are displayed in the figure for RRM1 and RRM2 domain, respectively.

The interaction of MSI-1 with oligo-L2 has been investigated by recording 2D ^1^H–^15^N–HSQC NMR spectra of the three protein constructs (MSI-1 RRM_1-2_ tandem domain, RRM1 and RRM2 isolated domains) before and after the addition of increasing amounts of the RNA strand. The interactions between RRM1 and RRM2 with oligo-L2 are in the slow exchange regime on the NMR timescale (Fig. 4 and Supplementary Fig. S1) in agreement with a high affinity of the oligonucleotide for the two proteins. SEC-MALS and kinetic SPR experiments confirmed a 1:1 binding model, with dissociation constants (*K*_D_) (Supplementary Figs S2–S4 and Table 1) in the nanomolar range (see Supplementary Results S2 and S3). Since the UAG sequence is included in the (G/A)U_1-3_AGU motif, the high affinity of RRM2 for oligo-L2 is not unexpected. Conversely, the analysis of the interaction between the MSI-1 RRM_1-2_ tandem domain and oligo-L2 is somewhat surprising. When the tandem domain is titrated with increasing amounts of oligo-L2, some cross-peaks of the free protein broaden and decrease in intensity (Fig. 4) with some signals experiencing small CSPs (Supplementary Fig. S5). At the same time, cross-peaks of a new species cannot be clearly detected, even with an excess of RNA with respect to the protein (protein/RNA molar ratio of 1:2). Interestingly, the signals of residues belonging to the β-sheet surface of both domains are affected by decreases in intensity upon the addition of oligo-L2 (Supplementary Fig. S5). This behavior suggests a competition between the two domains for the binding of oligo-L2. The line-broadening observed in the NMR titrations is also consistent with this scenario, since alternating binding of the oligo in fast to intermediate exchange regime to both RRMs, with varying chemical environments, can rationalize the observed line-broadening.

**Table 1.** Kinetic and affinity mean values of isolated domains with oligos 2–5 based on triplicates

	MSI-1 RRM1			MSI-1 RRM2		
	*k*_a1_ (M^−1^s^−1^)	*k*_d1_ (s^−1^)	*K*_D1_ (nM)	*k*_a1_ (M^−1^s^−1^)	*k*_d1_ (s^−1^)	*K*_D1_ (nM)
Oligo-L2	(3.5 ± 0.2) 10^6^	0.167 ± 0.010	47.1 ± 0.7	(4.6 ± 0.7) 10^6^	0.112 ± 0.007	25.0 ± 3.6
Oligo-L2.1	(2.6 ± 0.9) 10^6^	0.370 ± 0.062	151 ± 16.9	(1.8 ± 0.2) 10^6^	0.218 ± 0.008	123 ± 7.8
Oligo-L3	(4.5 ± 0.1) 10^5^	0.069 ± 0.003	153 ± 8.6	(4.7 ± 0.2) 10^5^	0.038 ± 0.0004	81.6 ± 2.0
Oligo-L3.2	(1.4 ± 0.3) 10^6^	0.120 ± 0.032	85.6 ± 7.4	(2.8 ± 1.2) 10^6^	0.121 ± 0.047	44.5 ± 4.5
Oligo-HP4	(8.3 ± 0.4) 10^6^	0.178 ± 0.013	21.9 ± 2.7	(1.1 ± 0.1) 10^6^	0.038 ± 0.004	37.2 ± 2.4
Oligo-HP5	(8.0 ± 0.5) 10^6^	0.268 ± 0.051	32.8 ± 5.2	(2.1 ± 0.1) 10^6^	0.055 ± 0.002	26.4 ± 1.6
	**MSI-1 RRM2 E180N (RRM2-M1)**			**MSI-1 RRM2 K182M (RRM2-M2)**		
	***k*_a1_ (M^−1^s^−1^)**	***k*_d1_ (s^−1^)**	***K*_D1_ (nM)**	***k*_a1_ (M^−1^s^−1^)**	***k*_d1_ (s^−1^)**	***K*_D1_ (nM)**
Oligo-L2	(8.4 ± 1.1) 10^6^	0.288 ± 0.015	35.5 ± 3.6	(9.6 ± 1.6) 10^5^	0.100 ± 0.01	108 ± 10.2
Oligo-L2.1	(6.7 ± 0.9) 10^6^	0.389 ± 0.044	58.6 ± 3.6	(3.5 ± 0.2) 10^6^	0.227 ± 0.05	62.9 ± 11.0
Oligo-L3	(7.0 ± 0.9) 10^6^	0.507 ± 0.003	74.8 ± 9.3	(1.5 ± 0.1) 10^6^	0.094 ± 0.002	64.5 ± 4.1
Oligo-L3.2	(4.2± 1.1)10^6^	0.187 ± 0.025	49.8 ± 11.4	(1.0± 0.05)10^6^	0.187 ± 0.005	88.5 ± 1.25
Oligo-HP4	(1.4 ± 0.3) 10^7^	0.178 ± 0.011	14.1 ± 2.5	(1.1 ± 0.7) 10^7^	0.177 ± 0.035	39.1 ± 15
Oligo-HP5	(2.6 ± 0.3) 10^7^	0.375 ± 0.036	14.5 ± 0.8	(7.7 ± 0.5) 10^6^	0.112 ± 0.026	14.5 ± 3.0

**Figure 5.**
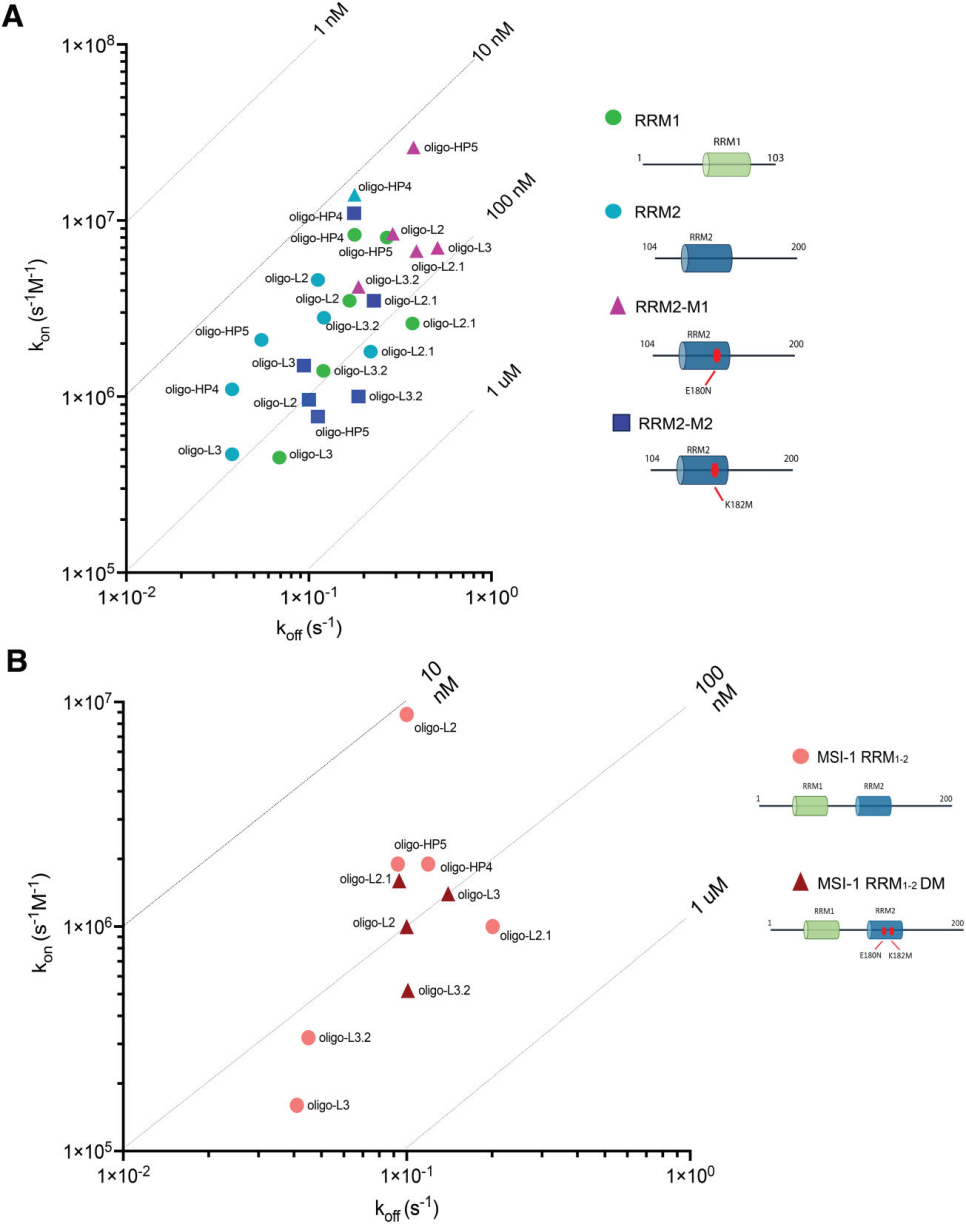
Iso-affinity kinetic plots showing the association rate constant (*y*-axis), dissociation rate constant (*x*-axis) and *K*_D_ values (diagonal lines) of (**A**) RRM1 (green circle), RRM2 (blue circle), RRM2-M1 (purple triangle), and RRM2-M2 (dark blue square) interacting with different RNA oligos (names written in the graph) and (**B**) wild-type MSI-1 RRM_1-2_ (pink circle) and double-mutated MSI-1 RRM_1-2_ DM (red triangle) interacting with different RNA oligos (names written in the graph).

The competition between the two domains in the interaction with oligo-L2 is further confirmed by the results of the kinetic studies, as presented in Fig. 5 and Table 1. The sensorgrams obtained by SPR analysis for the isolated RRM1 and RRM2 domains are very similar (Supplementary Fig. S3) and show interactions with fast association and dissociation rates. Conversely, for the MSI-1 RRM_1-2_ tandem domain, although a fast association is observed (Supplementary Fig. S3), a fast biphasic dissociation follows. Indeed, the dissociation is initially fast and then rapidly slows down, suggesting a subsequent stabilization of the interaction. This behavior has already been attributed to the formation of bivalent interactions between the tandem domain and distinct RNA strands, when the amount of unbound RNA is in excess [31]. The calculated affinities (*K*_D_) to MSI-1 RRM_1-2_ tandem domain are in the nanomolar range for the first monovalent interaction and in the lower micromolar range for the bivalent interaction (see Fig. 5, Table 1, and Supplementary Results S3).

To shed light on this competing phenomenon, we investigated how the two domains, both separately and in tandem, behave when two binding sites are present simultaneously in a linear RNA strand. Therefore, a new RNA oligonucleotide (oligo-L3: 5′-UUGUUAGUUAUUAGUU-3′) containing the consensus sequences known to bind both RRM1 and RRM2 domains, (G/A)U_1-3_AGU [28] and UAG motifs [26], respectively, has been designed. First, the two isolated RRM domains have been titrated separately with oligo-L3 and the interaction monitored by NMR. As observed for oligo-L2, the interactions of isolated RRM1 and RRM2 with oligo-L3 are in the slow exchange regime on the NMR timescale (Fig. 4 and Supplementary Fig. S6). The data indicate that RRM1 and RRM2 can bind both RNA motifs, with the formation of complexes with different protein/RNA stoichiometric ratios as described in detail in the Supplementary Results S4. The NMR data are supported by SEC-MALS analysis, which has been carried out to shed light on the stoichiometry of the interaction between RRM1 and oligo-L3. SEC-MALS chromatogram analysis confirms the presence of two species in solution, the most abundant one with a 2:1 protein/RNA stoichiometry, and a minor one with a 1:1 protein/RNA stoichiometry (see Supplementary Fig. S2 and Supplementary Results S4). Kinetics experiments show high-affinity binding, within the nanomolar range, between oligo-L3 and the isolated domains, with almost 8-fold slower association and <3-fold slower dissociation rate constants with respect to oligo-L2 (Fig. 5, Table 1, and Supplementary Results S3).

Then, we investigated the interaction of the tandem domain protein with oligo-L3. A decrease in the intensity of the cross-peaks of the free protein upon the addition of oligo-L3 is observed in solution NMR experiments. New cross-peaks, corresponding to the MSI-1 RRM_1-2_ tandem domain in complex with oligo-L3, appear and increase in intensity as well (Fig. 4 and Supplementary Fig. S6). Therefore, the interaction of the tandem domain with oligo-L3 occurs in a slow exchange regime on the NMR timescale, unlike the observed interaction with oligo-L2 (Fig. 4). However, in the presence of RNA at a concentration of 100 μM (1:1 protein/RNA molar ratio), the new signals are still broad, so the absence of multiple conformational states cannot be excluded. Assignment of the newly shifted signals is not feasible because of the higher ambiguity due to the higher crowding in the spectra of the MSI-1 RRM_1-2_ tandem domain.

**Figure 6.**
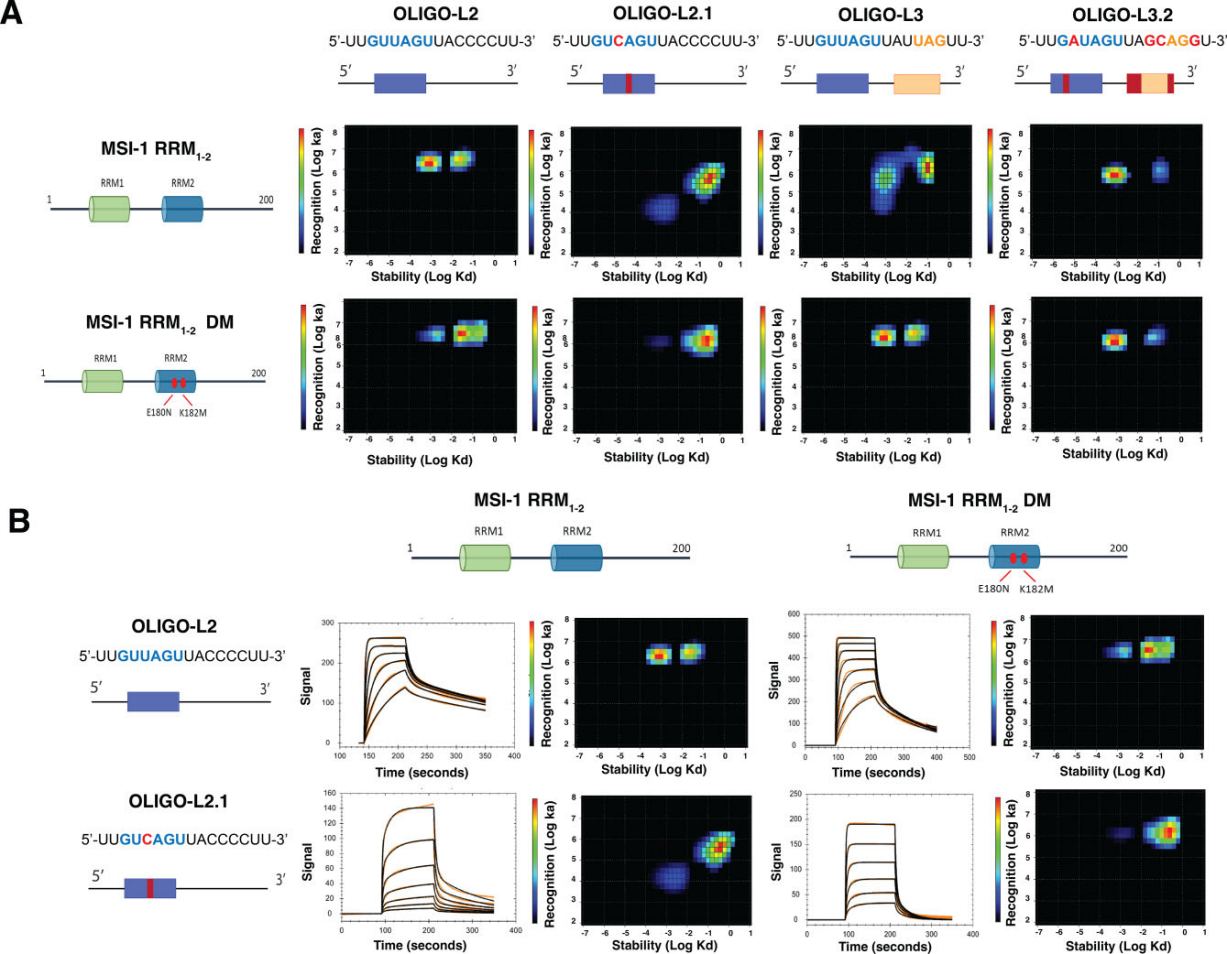
(**A**) Representative InteractionMap from MSI-1 RRM_1-2_ and MSI-1 RRM_1-2_ DM interacting with oligo-L2, oligo-L2.1, oligo-L3, and oligo-L3.2. (**B**) Sensorgrams and InteractionsMaps, corresponding to interactions of MSI-1 RRM_1-2_ with oligo-L2 with concentrations from 7.8 to 250 nM; MSI-1 RRM_1-2_ DM with oligo-L2 with concentration ranging from 7.8 to 500 nM; of MSI-1 RRM_1-2_ with oligo-L2.1 with concentration ranging from 7.8 to 500 nM and MSI-1 RRM_1-2_ DM with oligo-L2.1 with concentration ranging from 7.8 to 250 nM. MSI-1 RRM_1-2_ interactions with oligo-L2 and oligo L2.1, and MSI-1 RRM_1-2_ DM interaction with oligo-L2 were fitted with a 1:2 model (black line). MSI-1 RRM_1-2_ DM interaction with oligo-L2.1 was fitted with a 1:1 model (black line).

The kinetics of the interaction between the MSI-1 RRM_1-2_ tandem domain and oligo-L3 show a much slower association rate constant, resulting in a 4-fold weaker affinity compared to oligo-L2 (Fig. 5 and Table 2). This reduction in the association rate, that is also observed for the isolated RRM domains, is unexpected and InteractionMap analysis has been performed to further evaluate this behavior. InteractionMap shows that the peaks corresponding to the mono- and bivalent interaction are similar for all oligos containing the UAG motif (oligo-L2, -L3, -HP4, and -HP5), but for oligo-L3 these peaks are broader with respect to the association rate constant, indicating a heterogeneous recognition (Fig. 6A).

**Table 2.** Kinetic and affinity mean values of tandem domain protein (MSI-1 RRM_1-2_ and MSI-1 RRM_1-2_ DM) with oligos 2–5 based on triplicates

	MSI-1 RRM_1-2_				MSI-1 RRM_1-2_ DM			
	*k*_a1_ (M^−1^s^−1^)	*k*_d1_ (s^−1^)	*K*_D1_ (nM)	*K*_D2_ (μM)	*k*_a1_ (M^−1^s^−1^)	*k*_d1_ (s^−1^)	*K*_D1_ (nM)	*K*_D2_ (μM)
Oligo-L2	(8.8 ± 6.6) 10^6^	0.100 ± 0.015	57.6 ± 12.9	1.4 ± 0.3	(1.0 ± 0.2) 10^6^	0.100 ± 0.016	104 ± 9.5	10.6 ± 0.08
Oligo-L2.1	(1.0 ± 0.2) 10^6^	0.201 ± 0.05	218 ± 27.5	–	(1.6 ± 0.3) 10^6^	0.094 ± 0.022	67 ± 20.4	–
Oligo-L3	(1.6 ± 0.1) 10^5^	0.041 ± 0.003	248 ± 8.5	2.84 ± 0.2	(1.4 ± 0.2) 10^6^	0.140 ± 0.028	98.5 ± 4.8	2.3 ± 0.01
Oligo-L3.2	(3.2 ± 0.3) 10^5^	0.045 ± 0.001	144 ± 15.0	1.2 ± 0.2	(5.2 ± 0.06) 10^5^	0.101 ± 0.004	98.5 ± 6.9	1.9 ± 0.01
Oligo-HP4	(1.9 ± 0.1) 10^6^	0.119 ± 0.005	76.8 ± 5.9	3.1 ± 0.2	–	–	–	–
Oligo-HP5	(1.9 ± 0.1) 10^6^	0.093 ± 0.006	48.6 ± 5.6	3.0 ± 0.1	–	–	–	–

### How secondary structures in RNA affect the binding with human Musashi-1 protein

Binding of MSI-1 to folded RNA strands [i.e. hairpins (HP)] was then investigated using oligo-HP4 (5′-AAGCGUUAGUUAUUUAGUCGCUU-3′) and oligo-HP5 (5′-CACUCUGUAGUAUGUAGGGUUUAUUU-3′) that were selected because of the different locations of the consensus motifs, known to bind RRM1 and RRM2, respectively. Oligo-HP4 is an RNA fragment from the *NUMB* mRNA [28] that contains both the binding motifs (the (G/A)U_1-3_AGU motif for RRM1 and the UAG motif for RRM2) within the loop region of a hairpin folding (Schematics 1). Oligo-HP5 is an RNA fragment from the *DOUBLECORTIN* (DCX) mRNA [32] and contains one consensus motif (G/A)U_1-3_AGU in the loop region, and the second UAG motif in the double-stranded region of the hairpin (Schematics 1).

Solution NMR spectra show that in the presence of the hairpin constructs, the isolated RRM domains behave similarly to what is observed with the linear RNA sequence containing two binding sites (oligo-L3, see the previous section, Supplementary Results S5 and Supplementary Fig. S7). However, when the MSI-1 RRM_1-2_ tandem domain is titrated with the hairpin constructs, different behaviors are observed (see Supplementary Fig. S8). In particular, in the NMR titration of oligo-HP4 we observe only a decrease in signal intensity, without the appearance of new cross-peaks in the NMR spectra (Fig. 4). Conversely, when the MSI-1 RRM_1-2_ tandem domain is titrated with sub-stoichiometric concentrations of oligo-HP5, new cross-peaks corresponding to the protein in complex with RNA are observed (Fig. 4). Interestingly, also after the addition of an excess of the oligonucleotide with respect to the protein (∼200 μM, protein/RNA ratio of 1:2) the signals of the new species do not increase in intensity, and the signals of the free protein are still present in the spectrum. In this regard, competition between the two domains for the same RNA site, and the presence of multiple species in solution can be hypothesized. Furthermore, the interaction landscape may be complicated by the possibility of an opening of the hairpin structure (see Supplementary Results S5). Therefore, NMR data can give information on the binding regions, but not about the strength of the interaction.

**Figure 7.**
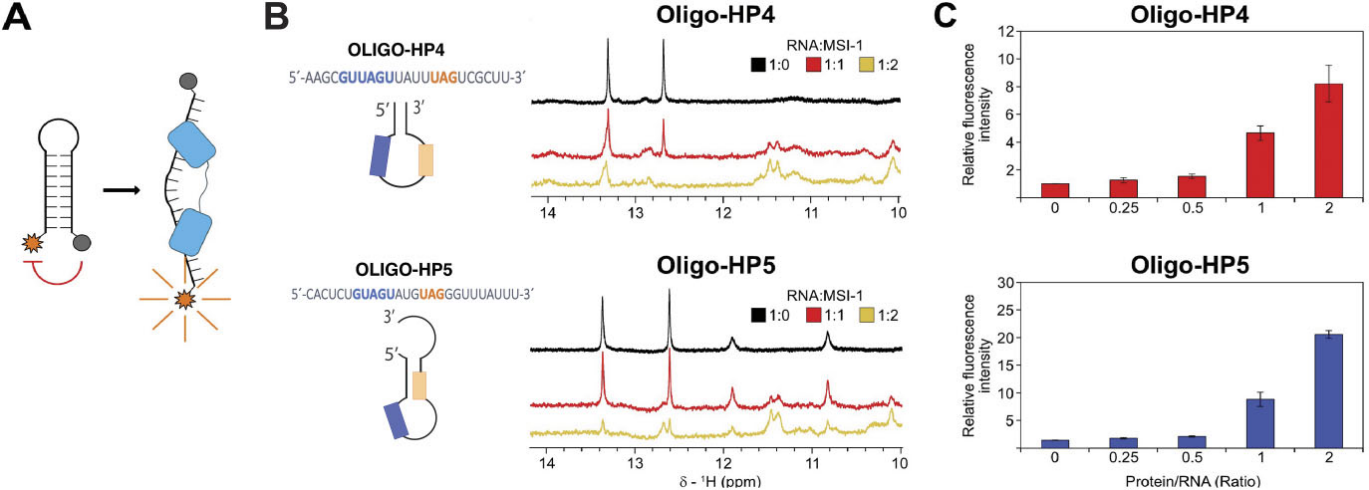
MSI-1 RRM_1-2_ binding to oligo-HP4 and oligo-HP5 involves unfolding of their RNA hairpin structures. (**A**) Schematic representation of the label RNAs used for fluorescence quenching assays highlighting the conjugated fluorophore at the 5′ (in orange) and the quencher at the 3′ (in gray). When the two probe are not close in space (i.e. upon unwinding of the hairpin by protein binding), there is fluorescence emission by the fluorophore. (**B**) Monitoring of oligo-HP4 and oligo-HP5 upon addition of MSI-1 RRM_1-2_ with imino NMR spectra and (**C**) fluorescence quenching assays (bar plots). For fluorescence quenching assays, fluorescence intensity values are normalized respect to the emission of the zero point of the titrations.

To verify whether the RNA hairpins are destabilized by binding of MSI-1, we performed fluorescence quenching assays using oligo-HP4 and oligo-HP5. Quenching of the fluorescence is seen for the hairpin as the fluorescent dye (6-FAM) and the quencher (BHQ1) are in close spatial proximity (Fig. 7) [33, 34]. The fluorescence quenching assay for the RNA-only and denaturing control samples shows a significant difference (Supplementary Fig. S9). Upon titration of the two oligos with MSI-1 RRM_1-2_, a concentration-dependent increase in the fluorescence intensity is observed compared to the control, consistent with the binding of MSI-1 RRM_1-2_ to the single-stranded form of the RNA. This is further confirmed by ^1^H imino NMR spectra monitored upon titration of oligo-HP4 and oligo-HP5 with MSI-1 RRM_1-2_. Reduction of the intensity of the imino signals upon the addition of increasing amounts of MSI-1 RRM_1-2_ (Fig. 7B) indicates unfolding of the RNA hairpin upon binding. Taken together, the fluorescence quenching assays, and NMR measurements indicate that both oligo-HP4 and oligo-HP5 adopt a hairpin conformation, which upon MSI-1 RRM_1-2_ binding is unfolded, leading the protein to interact with a single-stranded RNA conformation.

Kinetics studies show a similar binding of MSI-1 RRM_1-2_ tandem domain to both oligo-HP4 and oligo-HP5, resulting in comparable kinetic rate constants and affinity values for both the monovalent and bivalent interactions (Fig. 5 and Table 1). The affinities for the monovalent interactions are also similar to the values obtained for oligo-L2, while the affinities for the bivalent interactions for both oligo-HP4 and oligo-HP5 are similar to the affinity of the bivalent interactions obtained for oligo-L3. This confirms that the initial interaction (monovalent) of MSI-1 RRM_1-2_ with hairpins structures occurs in the same way as the interaction observed with the linear oligo bearing a single motif (oligo-L2), while the second interaction (bivalent) has a binding strength similar to that observed for the interaction with the linear oligo containing two binding motifs (oligo-L3).

### Designing substitutions for protein and RNA-binding sites to enhance the affinity and specificity of protein–RNA interactions

To investigate the molecular bases of competition between the two RRM domains for the same binding site, we have analysed the binding preferences of RRM1 and RRM2 for various RNA constructs using RRMScorer. First, we have assessed the interactions between each RRM with a generic 3-nucleotides sequence N_x_N_x_N_x_ (where N_x_ can be any nucleic base). Both RRMs display a clear preference for the UAG motif, as expected. However, RRM1 exhibits a slightly higher predicted score, with a 0.015 difference on a logarithmic scale (data not shown).

**Figure 8.**
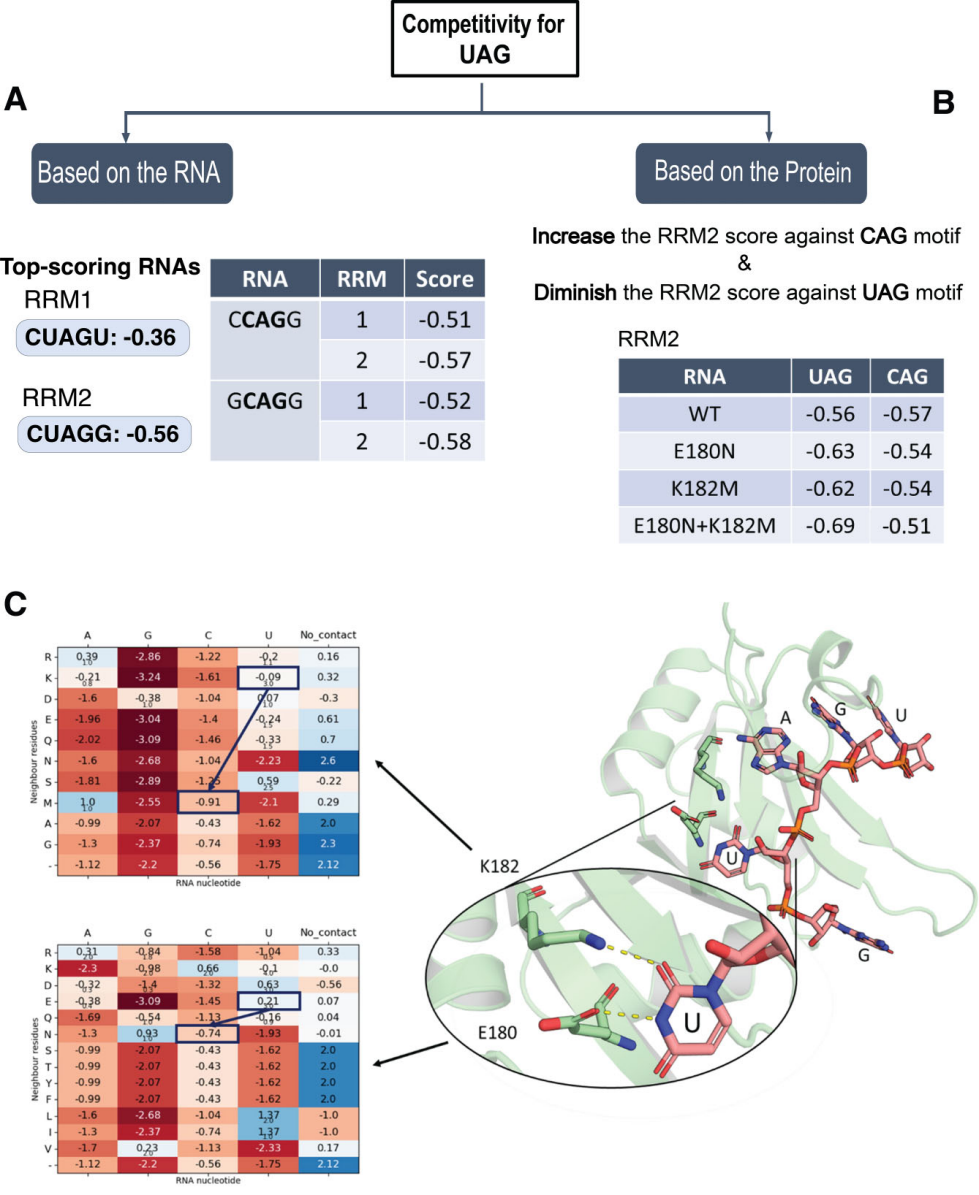
Scoring values from RRMScorer calculations. (**A**) On the left, overall top-scoring RNA motifs for the isolated domains. On the right, top-scoring RNA motifs for the CAG motif. (**B**) Highest scores and proposed mutations based on the protein sequence. (**C**) On the left, residue-level scores for the CAG motif at positions E180 and K182 in RRM2. On the right, structural view of the second RRM bound to the GUAGU motif. The selected substitutions, E180N and K182M, are designed to reduce the score toward uracil while maintaining a relatively higher score toward cytosine, although both scores remain negative.

Next, we have evaluated the top-scoring 5-nucleotide motif N_x_UAGN_x,_ for each RRM, again using RRMScorer. The best scores are obtained for the 5-nucleotide motif CUAGU and CUAGG for RRM1 and RRM2 domain, respectively, with a score difference between domains of 0.2, on the logarithmic scale (the better score of −0.36 is obtained for RRM1, while a worse score of −0.56 is obtained for RRM2, see Fig. 8A). The positions with best absolute scores and largest score differences between the two RRM domains are plotted in Supplementary Fig. S10. This analysis identifies RNA motifs that show a score drop for RRM1 relative to CUAGU, but not for RRM2, where the score remains close to that of the reference CUAGG motif.

Based on this, we hypothesized that an RNA construct containing two distinct short motifs, each specifically targeting one of the RRMs, could enhance the overall binding affinity of the tandem domain by avoiding competitive binding of the two domains for the same site. Because RRM1 and RRM2 have a similar predicted binding score for UAG, our goal was to find a motif that shows the highest possible score drop for RRM1, while maximizing the score for RRM2. This way, we aimed to design an RNA strand containing 3- to 5- nucleotide motif with low affinity for RRM1, but still good affinity for RRM2, to shift the binding preference of the second domain.

The CAG motif was identified as the most promising candidate. In particular, the sequences CCAGG and GCAGG were the best motifs to accomplish both RRM1 and RRM2 score shifts, to replace the canonical UAG-binding site in newly designed RNA strands (see Fig. 8A and Supplementary Fig. S10).

In addition to RNA engineering, we have explored the possibility of enhancing specificity and affinity through residue substitutions on the protein side, using RRMScorer in a similar manner. We have designed a variant of RRM2 with reduced affinity for UAG and increased affinity for CAG motif. For this purpose, we have analysed the possible residues involved in recognizing of the first pyrimidine of the 3-mer motif, aiming to switch preference from uracil to cytosine (from UAG to CAG) (Fig. 8B). According to the results provided by RRMScorer (Fig. 8B and C), we have identified E180N and K182M as the most promising substitutions to switch binding specificity toward the cytosine. However, other substitutions could also have potentially provided similar effects, as they share the same absolute difference in score (i.e E180A or K182S). Nevertheless, we have chosen the ones that gave the worst score possible for uracil, while maintaining good score to cytosine, with the goal to reduce or abolish the competition between RRM domains.

Structural analysis of the RRM2 domain in complex with GUAGU (PDB ID: 5X3Z) revealed, indeed, that residues E180 and K182 interact with uracil by engaging the amine and carbonyl groups of the nucleotide base, respectively (Fig. 8C). The new amino acids present in the modified protein (Asn and Met, respectively) maintain a similar overall steric bulk and are not expected to alter the RRM domain’s structure, while influencing the local charge distribution. Interestingly, these two residues are also present in the same position in other natural RRMs that preferentially bind RNA sequences with cytosine at the corresponding position.

### Experimental evaluation of the substitutions of the protein residues

First, the impact of each residue substitution on RRM2 designed with RRMScorer has been evaluated by analysing their binding to oligo-L2 by SPR. The RRM2 domain bearing the E180N substitution is called RRM2-M1 and the one containing the K182M substitution is called RRM2-M2. When binding oligo-L2, displaying an UAG motif, RRM2-M1 shows a slightly higher association rate constant and a 2-fold faster dissociation rate constant than RRM2, leading to a marginally lower affinity (Fig. 5 and Table 1, and Supplementary Figs S3 and S4). In contrast, RRM2-M2 displays an almost 7-fold slower association rate constant while keeping a similar dissociation rate constant, explaining the >5-fold weaker affinity.

After evaluating the effect of the single mutations on the binding, the impacts of the combined substitutions for the MSI-1 RRM_1-2_ tandem domain on binding to the previously tested and designed RNA oligonucleotides have been investigated. To evaluate if the mutations affect the affinity of the RRM2 domain for the (G/A)U_1-3_AGU motif, the MSI-1 RRM_1-2_ tandem domain, bearing the two mutations E180N and K182M (MSI-1 RRM_1-2_DM, hereafter), has been titrated with increasing amounts of oligo-L2 and NMR spectra recorded. After the addition of oligo-L2, the signals of the free protein in the 2D ^1^H–^15^N TROSY spectrum decrease in intensity, while new cross-peaks, corresponding to the complex between MSI-1 RRM_1-2_DM and oligo-L2 appear and increase in intensity (Fig. 4). More importantly, the signals of the free protein experiencing the largest decreases in intensity after the addition of RNA at a concentration of 25 μM to the protein solution (1:0.25 protein/RNA molar ratio), correspond to all (but two) residues located on RRM1 domain (Supplementary Fig. S11). Although two residues in the C-terminal region of the RRM2 domain still experience some effect, the two mutations on the RRM2 domain largely shift the binding preference of oligo-L2 toward the RRM1 domain. However, the broadening of the signals suggests that some heterogeneity is still present at equimolar concentrations of protein and RNA. This is corroborated by SPR data where a biphasic mode of interaction is still present. Both RRM domains still bind oligo-L2, but the affinity of the bivalent interaction is >7-fold reduced when the double mutation is present (Fig. 5 and Table 2). Also, InteractionMap analysis shows two peaks, representing the monovalent and bivalent interaction, but with a reduced affinity for the bivalent interaction of MSI-1 RRM_1-2_DM compared to MSI-1 RRM_1-2_ (Fig. 6B).

Then, the interaction of MSI-1 RRM_1-2_DM with oligo-L3 has been analyzed. Interestingly, after the addition of oligo-L3, at equimolar concentration with the protein, the linewidth of the new signals of the MSI-1 RRM_1-2_DM in complex with oligo-L3 is generally sharper than what has been observed for the wild-type protein, suggesting the formation of a single species in solution for the protein/RNA complex (see Supplementary Results S6, and Supplementary Figs S12 and S13). More importantly, the signals mostly affected by interaction are assigned to residues majorly located on the RRM1 domain, confirming the two mutations decrease the affinity of the RRM2 domain toward the UAG motif (see Supplementary Results S6). When the interaction of MSI-1 RRM_1-2_DM with oligo-L3 is characterized with SPR, the monovalent interaction has a 2.5 stronger affinity value, due to a 10-fold faster association rate compared with the non-mutated tandem binding (Fig. 5 and Table 2). The monovalent interaction of MSI-1 RRM_1-2_DM with oligo-L3 is also very similar to that with oligo-L2, indicating that the binding is no longer affected by the competition of the two RRM domains for the same motif, as observed for the interaction of the wild-type protein with oligo-L3. This is confirmed by InteractionMap analysis, which showed less heterogeneity with smaller and better-defined peaks when compared with the wild-type protein (Fig. 6A). Nevertheless, MSI-1 RRM_1-2_DM still has a relatively high affinity for the bivalent interaction which is <2-fold weaker compared to the interaction of the wild-type with oligo-L2, but >4-fold stronger than the MSI-1 RRM_1-2_DM interaction with oligo-L2 (Figs 5, 6, and Table 2).

Multiple interactions are also observed by NMR when MSI-1 RRM_1-2_ DM is titrated with oligo-HP4. In this case, more than one species seems to be still present in solution, as suggested by the broadening of the signals (Fig. 4, and Supplementary Figs S14 and S15). Nevertheless, in the presence of oligo-HP4, a lower heterogeneity is observed for the double mutant compared to the wild-type protein, as described in the Supplementary Results S6. However, as for wild-type MSI-1 RRM_1-2_, the opening of the hairpin structure may also be responsible for the observed conformational heterogeneity.

### Experimental evaluation of the nucleobase’s substitutions on the RNA sequence

To evaluate the preference of binding for the minimal RNA motif, we have designed oligo-L2.1 (5′-UUGUCAGUUACCCCUU-3′), where the UAG motif has been replaced by a CAG motif. When interacting with the isolated RRM1 and RRM2 domains, SPR data revealed a 3-fold and almost 5-fold weaker affinity, respectively. Conversely, RRM2-M1 and RRM2-M2, bearing mutations to shift the binding preference from UAG to CAG, showed a slightly (3-fold and less than 2-fold) stronger affinity with respect to wild-type RRM2 (see Fig. 5, Table 1 and Supplementary Fig. S4), mostly due to a better recognition.

These effects are more evident when the interaction of oligo-L2.1 with MSI-1 RRM_1-2_ tandem domain is evaluated. Oligo-L2.1 is expected to diminish the binding partitioning between the two domains and to exhibit a lower binding affinity than oligo-L2 for MSI-1 RRM_1-2_. This is confirmed by the 4-fold weaker affinity for monovalent binding. The bivalent interaction, although still present, provides a small contribution to the overall interaction. Therefore, only the values for the monovalent interaction are shown in Table 2. This negligible contribution is confirmed by InteractionMap analysis, which does not show a clear stabilized fraction compared to the previously obtained results with oligo-L2 (Fig. 6A). This indicates that the MSI-1 RRM_1-2_ reduces its ability to form bivalent interactions on the sensor chip for the CAG binding motif.

Compared to binding of MSI-1 RRM_1-2_ to oligo-L2.1, the mutated tandem protein shows a >3-fold increase in affinity for the monovalent interaction with improved recognition and slower dissociation. The affinity of MSI-1 RRM_1-2_ DM for oligo-L2.1 is in the same range as that of the wild-type protein for oligo-L2, -HP4, and -HP5 (Fig. 5 and Table 2, and Supplementary Results S8). As previously observed for the wild-type protein, the interaction is strongly dominated by a rapid 1:1 interaction and becomes similar to that observed for interactions of isolated RRMs with the oligo. The contribution of bivalent interactions to the overall sensorgrams is low at higher oligo immobilization levels, and becomes negligible at lower oligo densities. Therefore, binding curves have been analyzed using the 1:1 model for two replicates with lower RNA densities, while the 1:2 model has been applied to interactions at higher RNA densities, and only the results from the strongly dominating rapid interaction are reported in Table 2. The weak or insignificant contribution of bivalent interaction indicates that RRM2 is leading the recognition with oligo-L2.1. This was confirmed by InteractionMap analysis of the MSI-1 RRM_1-2_ DM interaction with oligo-L2.1 that shows a dominating peak corresponding to the monovalent binding (Fig. 6A).

NMR spectroscopy has been used to investigate the interaction of the modified RNA oligonucleotides bearing two binding sites (oligo-L3.2 (5′-UUGAUAGUUAGCAGGU-3′) and oligo-HP4.2 (5′-AAGCGAUAGUUAUGCAGGCGCUU-3′)) with the wild-type MSI-1 RRM_1-2_ protein and with the “double-mutant” MSI-1 RRM_1-2_DM. The interaction of the wild-type tandem domain protein with oligo-L3.2 is in the slow exchange regime on the NMR timescale, as observed with the original oligo-L3 (see Supplementary Results S7). However, at equimolar protein/RNA ratio, the signals of the wild-type MSI-1 RRM_1-2_ in the 2D ^1^H-^15^N TROSY spectrum appear split, suggesting the presence of multiple species in solution (Fig. 4). This effect is more evident than with the original oligo-L3, and it is not possible to rule out that one of the two RRM domains is not interacting with the RNA. As expected, the modification of the RNA-binding site weakens the interaction of the oligo with the wild-type MSI-1 RRM_1-2_ protein (see Supplementary Results S7). In this case, residues experiencing the largest effect are spread on both domains and not only on RRM1, as observed for the interaction of the double mutant with oligo-L3.

SPR experiments show a 2-fold faster association of MSI-1 RRM_1-2_ to oligo-L3.2 compared with oligo-L3, resulting in a 2-fold stronger affinity. This finding indicates that the substitution of the UAG with CAG motif reduces heterogeneous recognition of multiple motifs on the same RNA strand by RRM domains. Conversely, the double mutant exhibits similar affinities for the monovalent and bivalent interaction with oligo-L3 and oligo-L3.2. The double mutant MSI-1 RRM_1-2_ DM binds 2.6-fold slower to oligo-L3.2 compared to oligo-L3. However, the association is still more than three times faster than that observed for the interaction between the wild-type tandem domain and oligo-L3 (Table 2), and InteractionMap analysis for both the wild-type and mutated tandem domain with oligo-L3.2 shows well-defined peaks similar to those observed for oligo-L2 with a dominating peak corresponding to the monovalent binding (Fig. 6A). At the same time, the NMR data that show that, in the presence of oligo-L3.2 in a 1:1 molar ratio, a single species is visible for MSI-1 RRM_1-2_DM (Fig. 4).

The effects of oligo-HP4.2 on the wild-type MSI-1 RRM_1-2_ and MSI-1 RRM_1-2_DM have been also investigated with NMR spectroscopy (see Supplementary Results S7). Modifications on the RNA construct have weakened the interaction of the RRMs for the second binding site in order to disfavor the formation of possible species involving more than a single MSI-1 protein. Unfortunately, heterogeneity is still observed in solution with different combinations of protein–RNA binding modes: e.g. complexes with RRM1 or RRM2 bound to the (G/A)U_1-3_AGU binding site for both the wild-type and double-mutated proteins.

These findings corroborate the idea that the modifications present in oligo-HP4.2, when combined with mutations on the protein, do not prevent the formation of different complexes in which the (G/A)U_1-3_AGU motif can interact with either one or the other RRM domain.

### Investigating the effects of longer spacers between the two binding sites in RNA strands

To further investigate the structural factors influencing RNA recognition by the tandem domain, as well as how the two RRMs interact during RNA binding, new RNA strands have been designed starting from oligo-HP4. These strands feature longer spacers between the (G/A)U_1-3_AGU and UAG-binding sites.

The results obtained with oligo-HP4, which contains two recognition sites for the tandem domain on a hairpin structure, suggest a more complex and heterogeneous interaction than initially expected. Instead of observing the binding of both RRMs from the tandem domain to the same RNA to form single one to one complex, more species are present in solution. To exclude a possible steric hindrance between the two tethered RRMs caused by the too close proximity of the two binding sites in the oligo-HP4, we have designed new RNA strands (oligo-L3_2C and oligo-HP4_2C) with longer spacers. These two oligos incorporate two additional cytosine nucleotides between the (G/A)U_1-3_AGU and UAG-binding sites. The selection of these two cytosines and their specific positions were carefully chosen to prevent the formation of extra binding sites and to avoid elongating the existing ones.

The interaction of the designed oligos has been investigated by solution NMR on the MSI-1 RRM_1-2_DM, which has been shown to exhibit improved binding specificity for oligo-L3 and oligo-HP4. MSI-1 RRM_1-2_DM has been titrated with increasing amounts of oligo-L3_2C and oligo-HP4_2C, respectively, and the interaction monitored by NMR spectra. In each titration, after adding the oligo-L3_2C or oligo-HP4_2C, the signals of the free protein in the 2D ^1^H–^15^N TROSY spectrum show a decrease in intensity, while new cross-peaks, corresponding to the complex between MSI-1 RRM_1-2_DM and the RNAs, appear in the spectra and increase in intensity (Supplementary Fig. S16). However, after the addition of equimolar concentrations oligo-L3_2C or oligo-HP4_2C to the protein, we could not infer the formation of a single species in solution for the protein–RNA complex. This is proved by signal broadening and splitting (see Supplementary Fig. S16), suggesting a slightly lower binding specificity. The data suggest that the interaction between the protein and the new oligos is weaker than previously observed. This is evidenced by the lower reduction in intensity of the protein signals, especially from RRM2, when sub-stoichiometric amounts of both new oligos were present in solution (see Supplementary Figs S13, S15, and S17). This contrasts with the results obtained using the original oligos, in particular with oligo-L3 (see Supplementary Figs S13, S16, and S17).

The analysis of the interaction between MSI-1 RRM_1-2_WT and oligo-HP4_2C was also interesting. Similar to the findings with oligo-HP4, we observed a decrease in signal intensity in the NMR spectra, without the emergence of new cross-peaks. In addition to the reduction in intensity, some signals showed CSPs, indicating a semi-fast to intermediate exchange regime on the NMR timescale (Supplementary Fig. S16). In contrast to previous observations, when the protein was titrated with oligo-HP4, the interaction with oligo-HP4_2C involves many residues from both the linker region and the RRM2 domain. This is demonstrated by the plots showing decreases in signal intensity (see Supplementary Fig. S18).

### The interdomain linker contributes to RNA recognition and binding

The different role of the linker observed in the interaction of WT with oligo-HP4 and oligo-HP4_2C led us to further investigate its involvement in RNA recognition and binding.

The substitutions in the linker connecting the two domains have been designed to decrease the conformational space sampled by these domains and alter potential interactions between the linker and the RRMs. Two proline residues have been introduced to replace Phe-96 and Arg-98 in MSI-1 RRM_1-2_DM, resulting in a newly designed “mutant” referred to as MSI-1 RRM_1-2_TM, hereafter. The analysis of CSPs calculated from the 2D ^1^H–^15^N TROSY NMR spectra of MSI-1 RRM_1-2_DM and MSI-1 RRM_1-2_TM indicates that these substitutions impact not only the neighboring residues in the protein sequence, but also residues located on the β-platform of RRM1 (see Supplementary Fig. S19).

The interaction of MSI-1 RRM_1-2_TM with the original linear RNA, oligo-L3, and the modified hairpin RNA, oligo-HP4_2C, has been investigated using solution NMR. Notably, substituting the two linker residues with proline residues significantly reduces the affinity of MSI-1 for oligo-L3. In the 2D ^1^H–^15^N TROSY spectra of MSI-1 RRM_1-2_TM, the addition of increasing amounts of oligo-L3 leads to noticeable shifts in the protein signals, with minimal changes in line broadening (see Supplementary Fig. S16). This observation suggests that the binding of this oligo occurs in a fast exchange regime on the NMR timescale. Interestingly, only the residues belonging to the RRM2 domain are affected by CSP (see Supplementary Fig. S17), while the residues in the RRM1 domain do not participate in this interaction. This suggests that substitutions in the interconnecting linker can alter its flexibility, thereby affecting the conformational space accessible to both domains. As a result, there is a notable decrease in the binding affinity of MSI-1 RRM_1-2_TM compared to MSI-1 RRM_1-2_DM for the oligo-L3.

Also, the interaction of the protein with oligo-HP4_2C is weakened by linker substitutions, and only the RRM2 domain appears to be involved in the binding (see Supplementary Figs S16 and S18). Upon adding oligo-HP4_2C, we observe a CSP and a change in signal line width, indicating a slightly stronger interaction between MSI-1 RRM_1-2_TM and oligo-HP4_2C compared to oligo-L3 (see Supplementary Fig. S16). These findings suggest that placing the binding site within a hairpin secondary structure prepares the oligo for better interaction with the constrained MSI-1 RRM_1-2_TM.

## Discussion

The design of novel RRMs or RNA strands with high affinity could be relevant to investigating the regulation of gene expression, as well as in the discovery of *in vivo* RNA targets of RRMs with a still unknown function or interaction. In this study, we have used the computational tool RRMScorer to engineer variants of the human MSI-1 protein able to bind a novel RNA sequence with improved binding selectivity. Using complementary biophysical techniques, we have investigated how both the wild-type and mutant MSI-1 proteins interact with native and modified RNA strands to uncover the structural determinants of binding specificity.

Our data indicate that the wild-type human MSI-1 tandem domain protein recognizes both (G/A)U_1-3_AGU and UAG motifs across linear and structured RNAs with high affinity (Fig. 5, Tables 1, and 2). When binding to a linear oligo containing one binding site like oligo-L2, RRM1 and RRM2 compete for this site, leading to a biphasic dissociation pattern in SPR data. Among the four tested oligos, only oligo-L3 allowed simultaneous binding of both RRMs within a single MSI-1 molecule, forming a stable 1:1 complex.

This study also provides insights on how RNA secondary structure influences MSI-1 binding. Hairpin RNAs, such as oligo-HP4 and -HP5, which contain two binding sites, exhibited fast association kinetics, likely due to the hairpin conformation. NMR and SPR data confirmed high-affinity interactions to both oligos with both isolated domains, while SEC-MALS analysis showed that two RRM1 proteins bind oligo-HP4 simultaneously. Importantly, these hairpin structures did not inhibit binding, and their loop regions allowed accommodation of two RRMs without significantly altering the binding kinetics.

In contrast to what was previously observed with oligo-L2, NMR titrations of the tandem domain with oligo-HP4 indicated the presence of high molecular weight species involving two or more MSI-1 proteins. This might be due to partial hairpin unfolding upon MSI-1 binding, as supported by fluorescence quenching assays and ^1^H imino NMR spectra. The formation of intermolecular over intramolecular complexes, unlike the behavior observed with linear oligo-L3, suggests that the two RRMs within a single MSI-1 protein may not bind both hairpin sites simultaneously. In case of oligo-HP5, NMR experiments showed the persistence of the signals of the free protein together with the signals of the new species, even in the presence of an excess of the hairpin, implying that only one site of the oligo (likely the (G/A)U_1-3_AGU site on the loop), is being bound, and that its interaction with the second domain is prevented by the involvement of the second site (UAG) in the double strand structure. Furthermore, following this hypothesis, the structure of the MSI-1–RNA complex could make the second domain of the protein unavailable to bind a second oligo.

Multiple RRMs in RBPs is known to enhance specificity and affinity by engaging different consensus motifs cooperatively or independently [3–5, 35–40]. Interestingly, our data reveals a more complex interaction model for MSI-1, where the RRMs in tandem compete rather than cooperate. This highlights MSI-1 as a model system for understanding its RBP–RNA recognition.

The results obtained from the modification on the protein and oligonucleotides sequences designed by RRMScorer provided significant and intriguing data. SPR experiments helped elucidate the individual contributions of the RRM2 substitutions to binding kinetics and affinity. The E180N mutation (RRM2-M1) showed a higher affinity than RRM2-M2. With oligo-L2.1, which lacks the UAG motif and contains a single CAG motif, the increased affinity of RRM2 could largely be attributed to E180N mutation, although both contributed to an extent.

When testing the double mutant tandem domain protein, in the presence of oligo-L2, MSI-1 RRM_1-2_ DM exhibited roughly 2-fold and 10-fold reduced affinity for the (G/A)U_1-3_AGU motif in the monovalent and bivalent interactions, respectively. MSI-1 RRM_1-2_ DM showed a stronger monovalent interaction to oligo-L2.1, compared with the binding of both MSI-1 RRM_1-2_ and MSI-1 RRM_1-2_ DM to oligo-L2. This is mostly due to the improved affinity of modified RRM2 for the CAG motif, as expected from the RRMScorer prediction. The bivalent interaction, already weak in MSI-1 RRM_1-2_ DM with oligo-L2, is completely absent with oligo-L2.1.

Further evidence of altered domain interaction was observed in NMR titrations with oligo-L3, where MSI-1 RRM_1-2_ DM showed formation of a single protein-RNA complex, with RRM1 primarily responsible for spectral changes. This contrasts with the wild-type protein, which formed multiple species due to competition between domains for UAG sites. Improved selectivity for RRM1 was also noted when MSI-1 RRM_1-2_ DM was titrated with the folded oligo-HP4. Although some heterogeneity persisted, the reduced affinity of the modified RRM2 for UAG led to weaker effects on RRM2 and decreased conformational variability.

Introducing the CAG substitution in RNA strands restored the binding affinity for the modified RRM2 domain, leading to more specific complexes than those formed by wild-type MSI-1 with non-substituted RNAs. For instance, a single species was observed when oligo-L3.2 was titrated with MSI-1 RRM_1-2_ DM. SPR confirmed that this mutant displayed slightly higher affinity for oligo-L3.2, than the wild-type, with a faster association rate. This supports the improved selectivity conferred by the substitutions, also observed in the interactions conducted by NMR with hairpin constructs. When oligo-HP4.2 was added to MSI-1 RRM_1-2_ DM, only two predominant species seem to be present in solution. This behavior is significantly different from that of the wild-type MSI-1 in the presence of non-substituted oligo-HP4, where a larger heterogeneity was observed.

Although heterogenicity was not completely abolished, substitutions designed with RRMScorer proved to be efficient and a clear shift of specificity of the modified protein toward a newly designed RNA sequence was observed. In addition, we explored whether increasing the distance between the two binding sites in the RNA strand in both linear and hairpin RNA strands would affect the binding specificity and lead to a cooperative binding (potentially by eliminating steric clashes or expanding the conformational space). However, the introduction of two additional cytosines did not lead to an improvement in affinity. Instead, we observed a slight broadening of the signals experiencing chemical shift variations, suggesting a slight weaker interaction compared to the original oligos. These findings indicate that increasing the distance between the two binding sites did not result in cooperativity between the two domains with these oligos.

Moreover, substitutions of residues in the interdomain linker dramatically reduce the binding affinity. The linker can, indeed, influence RNA recognition and binding by (i) affecting the conformational space explored by the two domains, (ii) interacting directly with the RNA strand, and (iii) interacting with each domain. The loss of affinity was particularly pronounced in the RRM1 domain for the tested oligonucleotides. This decrease in affinity seems to be linked to changes in the interactions between the linker and the RRM1 domain, as suggested by the CSP observed in RRM1 domain. Additionally, the decreased affinity may also result from the limited capability of the two domains to reorient and explore conformational space.

In summary, this research focused on exploring the interactions of both the isolated single domains of MSI-1 and the tandem construct with a set of RNA oligos derived from the NUMB mRNA sequence, which is recognized for its interaction with the protein. Our results indicate the followings:

- Both domains exhibit a strong affinity for the two binding sites.
- When both domains are present in solution, forming a tandem domain, they compete for these sites.
- No cooperativity has been observed with these oligonucleotides, as the affinity of the tandem domain for oligonucleotides with two binding sites seems at most similar to that of the isolated domain for single sites. This suggests that the reduced heterogeneity of species, observed when the tandem domain interacts with oligonucleotides containing two binding sites, is due to the ability of the two domains to bind to both sites simultaneously, thereby minimizing competition between each other. Even with oligo-L3, which appears to form a dominant species with the tandem domain, there is no significant tethering effect from the linker. If there was a tethering effect, it would have notably increased the affinity of the tandem domain for the oligonucleotide, potentially resulting in the formation of a single species.
- For the investigated oligos, increasing the distance between the two binding sites does not result in cooperativity between the two domains with these oligos, since a decrease in the affinity and selectivity is observed.
- The linker is crucial for the interaction. Substitutions at this level could, indeed, significantly alter the protein’s function.

Overall, this study provides a comprehensive analysis of human MSI-1 RNA recognition and binding, as well as demonstrates the utility of computational design for engineering selective protein–RNA interactions. These findings provide new insights for favoring or disrupting protein–RNA interactions to modulate the action of MSI-1, associated with pathological conditions, thus laying the groundwork for developing new RNA-targeting tools and biotherapeutics.

## Supplementary Material

gkaf741_Supplemental_File

## Data Availability

The protein resonance assignment has been reported in the Biological Magnetic Resonance Data Bank (BMRB) under the accession code: 52590. The data underlying this article will be shared on request to the authors.

## Notes

Present address: MRC Protein Phosphorylation and Ubiquitylation Unit, University of Dundee, Dundee DD1 5EH, UK

Present address: Institute of Structural and Molecular Biology, University CollegeLondon, London WC1E 6BT, United Kingdom
