# Supplementary material for: Deciphering the RNA recognition by Musashi-1 to design protein and RNA variants for in vitro and in vivo applications
Source: Nucleic Acids Res. 2025 Aug 11;53(15):gkaf741. doi: 10.1093/nar/gkaf741 (PMC12342889; doi:10.1093/nar/gkaf741)
Supplement: gkaf741_Supplemental_File [file gkaf741_supplemental_file.docx]

**SUPPLEMENTARY DATA**

**Deciphering the RNA recognition by Musashi-1 to design protein and RNA variants for in vitro and in vivo applications**

Anna Pérez-Ràfols*^1,2,3^, Guillermo Pérez-Ropero^4,5^, Linda Cerofolini^1^, Luca Sperotto^6,7^, Joel Roca-Martínez^8,9,10^, Rosa Anahí Higuera-Rodríguez^11^, Pasquale Russomanno^1^, Wolfgang Kaiser^11^, Wim Vranken^8,9,12,13,14^, U. Helena Danielson^5,15^, Alessandro Provenzani^16^, Tommaso Martelli^2^, Michael Sattler^6,7^, Jos Buijs^4,17^ and Marco Fragai*^1^

^1^ Magnetic Resonance Center (CERM) and Department of Chemistry, University of Florence, and Consorzio Interuniversitario Risonanze Magnetiche di Metalloproteine (CIRMMP), Sesto Fiorentino 50019 (FI), Italy.

^2^ Giotto Biotech S.R.L, Sesto Fiorentino 50019, FI, Italy.

^3^ Present address: MRC Protein Phosphorylation and Ubiquitylation Unit, University of Dundee, DD1 5EH, Dundee, UK

^4^ Ridgeview Instruments AB, Uppsala SE 752 37, Sweden

^5^ Department of Chemistry – BMC, Uppsala University, Uppsala SE 751 23, Sweden

^6^ Institute of Structural Biology, Molecular Targets & Therapeutics Center, Helmholtz Munich, Neuherberg 85764, Germany

^7^ Bavarian NMR Center, Department of Bioscience, TUM School of Natural Sciences, Technical University of Munich, Munich 85748, Germany

^8^ Interuniversity Institute of Bioinformatics in Brussels, VUB/ULB, Brussels 1050, Belgium.

^9^ Structural biology Brussels, Vrije Universiteit Brussel, Brussels 1050, Belgium.

^10^ Present address: University College London, London, WC1E 6BT, UK

^11^ Dynamic Biosensors GmbH, Martinsried 82152, Germany.

^12^ AI lab, Vrije Universiteit Brussel, Brussels 1050, Belgium

^13^ Chemistry Department, Vrije Universiteit Brussel, Brussels 1050, Belgium

^14^ Biomedical sciences, Vrije Universiteit Brussel, Brussels 1050, Belgium

^15^ Science for Life Laboratory, Uppsala University, Uppsala SE 751 23, Sweden.

^16^ Department of Cellular, Computational and Integrative Biology (CIBIO), University of Trento, Trento 38123, Italy

^17^ Department of Immunology, Genetics and Pathology, Uppsala University, Uppsala SE 751 85, Sweden

*Corresponding authors e-mails: [aperezirafols001@dundee.ac.uk](mailto:aperezirafols001@dundee.ac.uk) ; [fragai@cerm.unifi.it](mailto:fragai@cerm.unifi.it)

**Table of Contents**

[Suppl. Methods 3](#_Toc200643646)

[Plasmids preparation 3](#_Toc200643647)

[Expression and purification of the recombinant isolated domains RRM1, RRM2, RRM2 E180N and RRM2 K182M of Human Musashi-1 (MSI-1) 3](#_Toc200643648)

[Expression and purification of recombinant MSI-1 RRM_1-2_, MSI-1 RRM_1-2_ DM and MSI-1 RRM_1-2_ TM 4](#_Toc200643649)

[R_1_, R_2_ and NOE measurements 4](#_Toc200643650)

[Surface Plasmon Resonance (SPR) 5](#_Toc200643651)

[Suppl. Results 5](#_Toc200643652)

[1. NMR characterization of free MSI. 5](#_Toc200643653)

[2. Interaction of the isolated MSI-1 RRM1 (1-103) and MSI-1 RRM2 (104-200) domains with a linear single-binding site RNA (oligo-L2). 6](#_Toc200643654)

[3. MSI-1 binding to oligos: Kinetics and affinity 6](#_Toc200643655)

[4. Interaction of isolated RRM domains with a linear RNA with two-binding sites RNA (oligo-L3). 7](#_Toc200643656)

[5. Interaction of isolated and tandem RRM domains with hairpin RNAs bearing two-binding sites (oligo-HP4 and oligo-HP5). 7](#_Toc200643657)

[6. Experimental evaluation of protein residues substitutions using NMR 8](#_Toc200643658)

[7. Experimental evaluation of nucleobase’s substitutions on the RNA sequence using NMR. 9](#_Toc200643659)

[8. Kinetic evaluation of UAG and CAG motif for the isolated wild-type and modified RRM2 domain 10](#_Toc200643660)

[Suppl. Tables and Figures 11](#_Toc200643661)

# Suppl. Methods

## Plasmids preparation

The plasmid encoding the full-length human Musashi-1 protein (hMSI-1, residues 1-261, Uniprot code: O43347) into the pET29 vector between the NdeI and XhoI sites and containing a Strep-Tag and TEV cleavage site at N-terminus was purchased by Twist Bioscience. To produce the MSI-1 human RRM1 domain (RRM1, residues 1-103), the hMSI-1 plasmid was modified by Site-Directed Mutagenesis to replace Met-104 with a stop codon.

The plasmid encoding the MSI-1 RRM2 domain (RRM2, residues 104-200) into the pET21a vector, between the NdeI and XhoI sites, and containing a Strep-Tag and TEV cleavage site at N-terminus was purchased by Twist Bioscience. To produce the RRM2 mutants, Site-Directed Mutagenesis was performed on the RRM2 plasmid to obtain the following mutations E180N (RRM2 E180N) or K182M (RRM2 K182M).

The gene encoding the native MSI-1 RRM_1-2_ into the pET29 vector, between the NdeI and XhoI, was purchased by Twist Bioscience. To produce the E180N/K182M mutant, Site-Directed Mutagenesis was performed on the gene MSI-1 RRM_1-2_ to obtain the double mutant construct MSI-1 RRM_1-2_ DM. The plasmid encoding the MSI- RRM_1-2_ (residues 1-200) containing the four mutations F96P/R98P/E180N/K182M (MSI-1 RRM_1-2_ TM) into the pET29 vector, between the NdeI and XhoI was purchased by Twist Bioscience.

## Expression and purification of the recombinant isolated domains RRM1, RRM2, RRM2 E180N and RRM2 K182M of Human Musashi-1 (MSI-1)

Recombinant human MSI-1 RRM1 and MSI-1 RRM2 (and its mutants) proteins were overexpressed in BL21(DE3) *E. coli* cells. Cells were grown in LB or M9 minimal media supplemented with ^15^NH_4_Cl or ^15^NH_4_Cl and ^13^C-glucose at 37 ºC until optical density (OD_600_) reached 0.6-0.8. Subsequently, expression was induced with 0.5 mM of isopropyl β-D-thiogalactoside (IPTG), cells were incubated at 37 ºC for 3 h and harvested by centrifugation at 4 ºC, for 15 min at 7500 rpm. Cell pellet was resuspended in lysis buffer [100 mM Tris-HCl pH 8.0, 150 mM NaCl, 1 mM EDTA, Proteases Inhibitor Cocktail (SIGMAFAST protease inhibitor cocktail tablets, EDTA-free, same used for all purifications)], ruptured by sonication and separated by centrifugation at 30000 rpm for 35 min at 4 °C. Soluble fraction was filtered with a 0.22 µm membrane and purified using a Strep-Tag HP 5 mL column. Elution fraction was collected and purified to homogeneity by size exclusion chromatography using a Hi Load 26/60 Superdex 75 pg column that was previously equilibrated in 50 mM Tris-HCl pH 7.5, 150 mM NaCl, 0.5 mM EDTA, Proteases Inhibitor Cocktail. The purified fractions were collected, and a treatment with 5% PEI solution was performed in order to remove DNA/RNA attached to the protein. The resulting suspension was centrifuged at 7800 rpm for 40 min at 4 °C to remove the precipitate containing the PEI-nucleic acid complexes. The protein in solution was recovered from the excess of PEI in the supernatant by precipitation with 70% ammonium sulfate. After overnight incubation at 4 °C, this suspension was centrifuged at 7800 rpm for 40 min at 4 °C, and the pellet containing the protein was re-suspended in 70% ammonium sulfate and washed two time with the same buffer to eliminate PEI. Finally, the supernatant was discarded; the MSI-1 RRM1 protein was resuspended in RNAse free buffer [50 mM Tris-HCl, pH 7.5, 140 mM NaCl, 0.5 mM EDTA, Proteases Inhibitor Cocktail] and filtered with a 0.22 µm membrane, whereas MSI-1 RRM2 protein (and its mutants) was resuspended in RNAse free buffer [50 mM Tris-HCl, pH 7.2, 140 mM NaCl, 1 mM EDTA, Proteases Inhibitor Cocktail].

## Expression and purification of recombinant MSI-1 RRM_1-2_, MSI-1 RRM_1-2_ DM and MSI-1 RRM_1-2_ TM

Recombinant human MSI-1 RRM_1-2_ and the other protein variants were overexpressed in BL21(DE3) GOLD *E. coli* cells. Cells were grown in LB or M9 minimal media supplemented with ^15^NH_4_Cl or ^15^NH_4_Cl and ^13^C-glucose at 37 ºC until optical density (OD_600_) reached 0.6-0.8. Subsequently, expression was induced with 0.5 mM of isopropyl β-D-thiogalactoside (IPTG), cells were incubated at 37 ºC for 4 h and harvested by centrifugation at 4 ºC, for 15 min at 7500 rpm. Cell pellet was resuspended in lysis buffer [50 mM Tris-HCl, pH 8.0, 500 mM NaCl, 10% glycerol, Proteases Inhibitor Cocktail], ruptured by sonication and separated by centrifugation at 30000 rpm for 35 min at 4 °C. Soluble fraction was collected and a treatment with 5% PEI solution was performed. The resulting suspension was centrifuged at 7800 rpm for 40 min at 4 °C to remove the precipitate containing the PEI-nucleic acid complexes. The protein in solution was recovered from the excess of PEI in the supernatant by precipitation with 70% ammonium sulfate and washed as described before for the single domains. Finally, the supernatant was discarded; the proteins were resuspended in 20 mM Tris-HCl, pH 9.0, Proteases Inhibitor Cocktail buffer. Soluble proteins were filtered with a 0.22 µm membrane and purified by an ion exchange chromatography using an Anion exchange Q FF 16/10 column previously equilibrated in 20 mM Tris-HCl, pH 9.0, Proteases Inhibitor Cocktail. Elution was performed against 20 mM Tris-HCl, pH 9.0, 1 M NaCl, Proteases Inhibitor Cocktail. The proteins were filtered and further purified to homogeneity by size exclusion chromatography using a Hi Load 26/60 Superdex 75 pg column that was previously equilibrated in 50 mM Tris-HCl, pH 7.8, 150 mM NaCl, Proteases Inhibitor Cocktail buffer. The purified fractions were collected, and the buffer was exchanged using a HiPrep 26/10 Desalting column, previously equilibrated with the RNAse free buffer, for the NMR assignment experiments [20 mM MES, pH 6.0, 100 mM NaCl, 0.5 mM EDTA, Proteases Inhibitor Cocktail] or the RNAse free buffer for NMR titration experiments [50 mM Tris-HCl, pH 7.2, 140 mM NaCl, 1 mM EDTA, Proteases Inhibitor Cocktail].

## R_1_, R_2_ and NOE measurements

Experiments for the determination of ^15^N longitudinal and transverse relaxation rates and ^15^N- ^1^H NOE were recorded on a Bruker AvanceNEO NMR spectrometer operating at 700 MHz (^1^H Larmor frequency) using a ^15^N- enriched sample of the human MSI-1 RRM_1-2_ tandem domain protein at 298 K [protein concentration of 140 µM in 20 mM MES pH 6.0, 100 mM NaCl, 1 mM DTT, 1 mM protease inhibitors]. ^15^N Longitudinal relaxation rates (R_1_) were measured using a sequence modified to remove cross-correlation effects during the relaxation delay.^29^ Inversion recovery times ranging between 20-2000 ms, with a recycle delay of 3.5 s, were used for the experiments. ^15^N transverse relaxation rates (R_2_) were measured using a Carr-Purcell-Meiboom_Gill (CPMG) sequence ^29^, with delays ranging between 16.96 and 220.48 ms and a re-focusing delay of 450 µs. Longitudinal and transverse relaxation rates were determined by fitting the cross-peak intensities as a function of the delay to a single-exponential decay using the Topspin Dynamic Center software. Heteronuclear NOE values were obtained from the ratio of the peak height for ^1^H-saturated and unsaturated spectra.

## Surface Plasmon Resonance (SPR)

SPR based experiments were performed by immobilizing different RNA strands on a streptavidin coated gold chip (SCB-SAD 200M and SCBS-SAD 200M, Xantec, Dusseldorf, Germany) as described in ^30^. The different proteins were diluted in a two-fold manner with concentrations ranging from 1 μM to 1.9 nM and injected sequentially from the lowest to the highest concentration in running buffer (50 mM Tris-HCl, 140 mM NaCl, 0.5 mM EDTA and 0.05% Tween) at 25 ºC. The surface was regenerated after each cycle by injecting a regeneration buffer solution (1 M NaCl, 1 M MgCl_2_). The flow rate was 60 µL/min for the RRMs and 100 µL/min for the tandem MSI-1 RRM_1-2_. The association time varied between 1 to 2 min and the dissociation time between 2 to 3 min.

The RRMs interactions and MSI-1 RRM_1-2_ DM interaction with **oligo-L2.1** results were fitted with a 1:1 model. MSI-1 RRM_1-2_ and MSI-1 RRM_1-2_ DM interactions were fitted with a heterogeneous ligand model and afterwards the rapid 2:1 method was applied to calculate the bivalent affinities. **Oligo-L3** and **oligo-L2.1** interactions with MSI-1 RRM_1-2_ and MSI-1 RRM_1-2_ DM were further analysed with InteractionMap (Ridgeview Diagnostics AB, Uppsala, Sweden). All interaction models and analysis methods are described in detail ^30^.

# Suppl. Results

## NMR characterization of free MSI.

To characterize the protein dynamics, measurements of longitudinal (R_1_) and transverse (R_2_) relaxation rates of the backbone amide nitrogen were performed on a ^15^N – enriched sample of the MSI-1 RRM_1-2_ construct. Theoretical estimates of R_1_ and R_2_ values and of the rotational correlation time were calculated by HydroNMR from the X-ray structures of the isolated RRM1 (residues 20-96, PDB 2RS2) and RRM2 (residues 110-186, PDB 5X3Z) domains, and from a model of the RRM_1-2_ tandem domain (residues 20-186) and are displayed together with the experimental data in **Figure 3**. The experimental rotational correlation time obtained from R_1_, R_2_ and NOE experimental data (8.4 ± 0.4 ns) is in between the calculated values for the isolated (5.8 ns for both domains) and tandem domains (14.9 ns). This indicates that the RRM1–RRM2 construct does not behave as a rigid body but displays instead inter-domain flexibility, simulating a protein of lower molecular weight. In detail, the comparison of experimental R_1_ and R_2_ data with theoretical values calculated for the isolated RRM1 and RRM2 domains shows that experimental R_1_ values are smaller and R_2_ values are larger than their theoretical counterparts (**Figure 3, panels A, B**). At the same time, experimental R_1_ values are higher and experimental R_2_ values are lower than theoretical estimates calculated for the MSI-1 RRM_1-2_ construct (**Figure 3, panels C, D**), indicating interdomain dynamics. Further indication of the presence of inter-domain flexibility is provided by the ^15^N–^1^H NOE for the linker residues, experiencing values lower than 0.5 (from Ala95 up to Lys109, see **Figure 3, panel E**). The small NOE values of the residues in the linker between the two domains are evidence of fast motions on ps-ns timescale (faster than the overall protein-tumbling rate). Very small NOE values are found also for the N- and C-terminal tails (from Thr3 up to His17 and from Gln185 up to Ser200), indicating fast motions also at this level.

## Interaction of the isolated MSI-1 RRM1 (1-103) and MSI-1 RRM2 (104-200) domains with a linear single-binding site RNA (oligo-L2).

During the NMR titration, upon the addition of increasing concentrations of the **oligo-L2** to the solution of RRM1 or RRM2, the intensity of the signals of the free protein in the 2D ^1^H-^15^N HSQC decrease in intensity, while new cross-peaks, corresponding to the complex with **oligo-L2**, appear and increase in intensity (**Figure 4**). This indicates that both interactions are in the slow exchange regime on the NMR timescale. As expected, the signals of the free protein experiencing the largest decrease in intensity after the addition of RNA at the concentration of 25 µM to the protein solution (1:0.25 protein/RNA molar ratio) correspond to residues located in the β-platform as well as in loop-1 (β_1_-α_1_) and loop-3 (β_2_-β_3_) of the isolated domain (**Figure S1A**). In the presence of RNA at the concentration of 100 µM (1:1 protein/RNA molar ratio), the signals of the free protein in both cases have completely disappeared, while the signals of a new single homogeneous species are visible. The new signals for each complex have been partially reassigned, and the analysis of the chemical shift perturbation (CSP) performed (**Figure S1B**). The residues corresponding to the assigned signals experiencing the largest changes are located in the β-platform as well as in loop-1 (β_1_-α_1_) and loop-3 (β_2_-β_3_) of the isolated domain and correspond to the already described binding area for the isolated domain (PDB: 2RS2^27^ and 5X3Z ^26^) for RRM1 and RRM2, respectively. Unfortunately, many signals could not be reassigned because of their too large CSP. These signals belong as well to residues located on the β-platform.

Binding was corroborated in both cases by SEC-MALS analysis, which also provided information about the binding stoichiometry. First, the isolated domains and **oligo-L2** were analysed separately to ascertain the monomeric form of each isolated macromolecule (**Figure S2 panels A, B and C**). Then, the interaction of each RRM with **oligo-L2** at the protein/RNA molar ratios of 1:0.50 and 1:1 was evaluated. Protein Conjugate Analysis shows a clear 1:1 interaction, confirming the ability of both domains to bind the same RNA sequence (**Figure S2 panels G and H**).

## MSI-1 binding to oligos: Kinetics and affinity

Kinetic rate constants and affinity values were quantified for a series of RRM-RNA interactions (**Table 1-2**). Results with **oligo-L2**, **oligo-L2.1**, **oligo-HP4** and **oligo-HP5** were previously published in Pérez-Ropero et al ^30^, while the interactions with **oligo-L3** and **-L3.2** have not been previously published. All obtained sensorgrams for RRM1 and RRM2 were characterized by a fast association and dissociation rates as shown in **Table 1**, with affinity values in the nanomolar range. Single RRMs have a single binding domain for RNA, therefore they are only able to bind a single RNA molecule in a 1 to 1 fashion. Consequently, a 1:1 diffusion corrected model was employed to fit the data, rendering an optimal fitting for all interactions (**Figure S3 and S4**).

MSI-1 RRM_1-2_ binding to RNA shows a fast association followed by a biphasic dissociation, which is fast during the initial few seconds and slows down rapidly suggesting a complex stabilization of the interaction (**Figure S3**). This binding has been previously described as bivalent, and a method to quantify the association rates of the monovalent fraction, and the affinity of both mono and bivalent components was presented ^30^. This method was used to analyze sensorgrams for RRM_1-2_ interaction and resulting values are presented in **Table 1**. RRM1, RRM2 and the monovalent interaction of RRM_1-2_ interacted similarly with **oligo-L2**, with RRM2 showing a slightly stronger affinity, nonetheless affinities were similar ranging from 25 to 58 nM. Bivalent affinity of **oligo-L2** was 1.4 µM.

Both RRMs had similar affinity values in the double-digit nanomolar range when interacting with **oligo-HP4** and -**HP5**. As previously mentioned, these oligos harbour two binding motifs and they have been proved to form hairpins in solution. RRM1 had between 4 and 8-fold faster association rate constant for **oligo-HP5** and **-HP4**, respectively, and around 5-fold faster dissociation rate constant than RRM2 for both **oligo-HP4** and **-HP5**. However, the affinity values were similar despite the differences in the kinetic profile (**Figure S3, Table 1**).

## Interaction of isolated RRM domains with a linear RNA with two-binding sites RNA (oligo-L3).

During the NMR titrations, upon the addition of increasing concentrations of **oligo-L3** to either RRM1 or RRM2 domain, a decrease in the intensity of the cross-peaks of the free protein is observed, while new cross-peaks, corresponding to the RRMs in complex with **oligo-L3**, appear and increase in intensity (**Figure 4**). Therefore, also the interaction of RRM1 and RRM2 with **oligo-L3** is in the slow exchange regime on the NMR timescale. As observed for **oligo-L2**, the signals of the free proteins experiencing the largest decreas in intensity, after the addition of **oligo-L3** at the concentration of 25 µM to the protein solution (1:0.25 protein/RNA molar ratio), correspond to residues located in the β-platform (**Figure S6 panels A and C**). Interestingly, after the addition of **oligo-L3** at the concentration of 50 µM (1:0.5 protein/RNA molar ratio), for both RRMs, the signals of the free protein have almost completely disappeared, while the new appeared signals are very broad. This phenomenon can be explained by the occurrence of multiple heterogeneous species in solution. Indeed, RRM1 and RRM2 can bind both RNA binding sites, with the formation of complexes with different protein/RNA stoichiometric ratios. Instead, in the presence of RNA at the concentration of 100 µM (1:1 protein/RNA molar ratio) the linewidth of these new signals sharpens (**Figure 4**). The CSP between the resonances of the new partially reassigned signals of the complexes and the resonances of the free proteins is displayed in **Figure S6, panel B and C**. The residues corresponding to the assigned signals experiencing the largest changes are located in the β-platform. Unfortunately, also in this case, many signals could not be reassigned because of their too large CSP. These signals belong as well to residues located on the β-platform. As expected, most of the affected residues are in common between **oligo-L2** and **oligo-L3**.

SEC-MALS analysis was also carried out in this case to shed light on the stoichiometry of the interaction between RRM1 and **oligo-L3** and support the NMR data. The interaction of RRM1 with **oligo-L3** was evaluated at the protein/RNA molar ratio of 1:0.50. The Protein Conjugate Analysis confirmed the presence of two species in solution, the most abundant one with a 2:1 protein/RNA stoichiometry and a minor one with a 1:1 protein/RNA stoichiometry **Figure S2 panel I**. Moreover, no free protein was observed.

## Interaction of isolated and tandem RRM domains with hairpin RNAs bearing two-binding sites (oligo-HP4 and oligo-HP5).

The isolated RRM domains display a similar behaviour to what is observed with the linear RNA sequence with two binding sites (**oligo-L3**). The interactions of isolated RRM1 and RRM2 with both hairpin **oligo-HP4** and **oligo-HP5** are in the slow exchange regime on the NMR timescale (**Figure 4**). The effects due to **oligo-HP4** and **oligo-HP5** are also located on the β-platforms of the isolated domains (**Figure S7**). Also in these cases, the formation of multiple heterogeneous species cannot be excluded in the presence of RNA in sub-stoichiometric concentrations with respect to the protein.

SEC-MALS experiments were performed to shed light on the stoichiometry of the complexes. First, the isolated RRM1 domain, **oligo-HP4** and **oligo-HP5** were individually analysed to ascertain the monomeric form of each isolated macromolecule (**Figure S2 panel E and F**). For both the **oligo-HP4** and RRM1, a monomeric form was detected, however, aggregates of RNA were observed with **oligo-HP5**. Then, the interaction of RRM1 with **oligo-HP4** and **oligo-HP5** at different protein/RNA molar ratios was evaluated. The presence of species with different reciprocal protein/RNA stoichiometric ratio was observed in solution (**Figure S2, panel J and K**), confirming the heterogeneity detected through NMR experiments. We selected the chromatogram corresponding to an interaction of RRM1 and **oligo-HP4** with a molar ratio of 1:0.5 and attempted to analyze it with the Protein Conjugate Analysis. A mixture of complexes with the protein/RNA stoichiometric ratios of 2:1 and 1:1 was detected. Additionally, free RNA was present. However, the amount of each component cannot be determined with precision, due to the overlapping of the peaks.

We next investigated the interaction of **oligo-HP4** and **oligo-HP5** with the tandem domain MSI-1 RRM_1-2_ protein through solution NMR. In the NMR titration of the MSI-1 RRM_1-2_ with **oligo-HP4** we observed only a decrease in signal intensity without the appearance of new cross-peaks (**Figure S8**). In the presence of RNA, at the protein/RNA molar ratio of 1:0.25, a general decrease of the protein signals is observed (**Figure 4**) with some residues experiencing a larger effect (Ser15, Ile24, Gly25, Gly26, Leu50, Met52, Gly64, Phe65, Val66, Val94, Ala95, Arg107, Thr108, Val113, Gly115, Leu140, Met141, Phe142, Thr146, Arg150, Lys183, Ale184, Gln185, Glu188 and Met190) and few residues experiencing minor CSP (Asp14, Lys21, Met22, Ile24, Cys49, Met52, Lys58, Gly64, Phe65, Phe68, Ala100, Lys109, Gly115, Lys134, Gly153, Phe154, Val155, Ile162, Glu164, Lys165, Val166, Glu180, Ala184, Gln185, Glu188 and Met190).

In the titration of MSI-1 RRM_1-2_ tandem domain with **oligo-HP5**, instead, in the presence of the sub-stoichiometric concentrations of **oligo-HP5** with respect to Musashi-1, the appearance of new cross-peaks corresponding to the protein in complex with the RNA has been observed (**Figure S8**). However, also after the addition of RNA in excess with respect to the protein (~ 200 μM, protein/RNA ratio of 1:2) the signals of the new species do not increase in intensity, and the signals of the free protein are still present (**Figure 4**). Competition in the binding of the same RNA site between the two domains may be present, and multiple species formed in solution. Furthermore, the interaction landscape may be complicated by the possibility of an opening of the hairpin structure. Therefore, NMR data can give information only about the binding regions, but not about the strength of the interaction. In this respect, the residues experiencing the largest changes are located in the same region of those interacting with the other oligos (Ser15, Ile24, Gly26, Met52, Gly64, Val94, Ser117, Asp124, Val135, Met139, Leu140, Met141, Thr146, His149, Gly153, Val163, Ala184, Val189, see Figure S7).

## Experimental evaluation of protein residue substitutions using NMR

Next, we have studied by solution NMR the effect of the two protein residue substitutions on the interaction of Musashi-1 with RNAs containing two binding sites: i) a linear single stranded RNA (**oligo-L3**), and ii) a folded RNA bearing the two binding motifs within the loop region of a hairpin folding (**oligo-HP4**).

Upon the addition of increasing concentrations of the **oligo-L3** to the solution of MSI-1 RRM_1-2_ DM, a decrease in intensity of the signals of the free protein and the appearance of new cross-peaks were observed, as previously obtained for the wild-type protein (**Figure 4, S12 and S13**). The signals of the free protein experiencing the largest decreases in intensity after the addition of RNA at the concentration of 25 µM to the protein solution (1:0.25 protein/RNA molar ratio) correspond to residues mainly located in the RRM1 domain (Asp14, Ser15, Met22, Gly25, Gly26, Thr31, Leu50, Ser60, Arg61, Phe65, Val66, Phe68, Lys76, Leu85, Lys93, Ala95, Val155, Ala184, Gln185 and Lys187) (**Figure S13, position A and B i and iii**). More interestingly, in the presence of RNA in a 1:1 molar ratio with respect to the protein, the linewidth of the signals of the mutant is sharper than what observed for the wild-type protein. These data suggest the formation of a single species in solution for the protein/RNA complex.

The interaction of MSI-1 RRM_1-2_ DM with **oligo-HP4** has been then performed. In this case, we see a different behaviour with respect to what has been observed for the wild-type protein. Indeed, upon the addition of increasing concentrations of the **oligo-HP4** to the solution of MSI-1 RRM_1-2_ DM, the intensity of the signals of the free protein in the 2D ^1^H-^15^N TROSY spectrum decrease in intensity, while new cross-peaks, corresponding to MSI-1 RRM_1-2_ DM in complex with **oligo-HP4**, appear and increase in intensity (**Figure 4, S14 and S15**). As observed for **oligo-L3,** the signals of the free protein experiencing the largest decreases in intensity after the addition of RNA at the concentration of 25 µM to the protein solution (1:0.25 protein/RNA molar ratio) correspond to residues mainly located in the RRM1 (Asp14, Ser15, Met22, Gly25, Gly26, Thr31, Leu50, Ser60, Arg61, Phe65, Val66, Phe68, Lys76, Leu85, Lys93, Ala95, Val155, Ala184, Gln185 and Lys187) (**Figure S15, position A and B i and iii**). Therefore, lower heterogeneity is observed for the double mutant protein in the interaction with **oligo-HP4** with respect to the wild-type. However, more than one species seems to still be present in solution, as indicated by the broadening of the signals (**Figure 4 and S14**).

## Experimental evaluation of nucleobase’s substitutions on the RNA sequence using NMR.

The interaction of **oligo-L3.2** with the wild-type protein has been first investigated. During the NMR titration, upon the addition of increasing concentrations of **oligo-L3.2** to MSI-1 RRM_1-2_ WT protein, a decrease in the intensity of the cross-peaks of the free protein is observed, while new cross-peaks, corresponding to the wild-type tandem domain protein in complex with **oligo-L3.2**, appear and increase in intensity (**Figure 4 and S12 position B**). Therefore, also the interaction of the wild-type tandem domain protein with **oligo-L3.2** is in the slow exchange regime on the NMR timescale, as observed with **oligo-L3**. The signals of the free proteins experiencing the largest decreases in intensity after the addition of **oligo-L3.2** at the concentration of 25 µM to the protein’s solution (1:0.25 protein/RNA molar ratio) correspond to residues located in the β-platform, as well as in loop-1 (β_1_-α_2_) and loop-3 (β_2_-β_3_) of both RRMs (**Figure S13, position A and B i and ii**). (Asp4, Ile24, Gly25, Gly26, Gly64, Val94, Arg107, Val113, Gly114, Val118, Met139, Glu180 and Val189).

The effect of **oligo-HP4.2** mutant on the wild-type MSI-1 RRM_1-2_ protein has been then investigated. Upon the addition of the RNA to the protein solution, the signals of the free protein in the 2D ^1^H-^15^N TROSY decrease in intensity, while new cross-peaks, corresponding to MSI-1 RRM_1-2_ WT in complex with **oligo-HP4.2**, appear and increase in intensity, unlike what was observed in the presence to **oligo-HP4** (**Figure 4 and S14 position B**). The signals of the free protein experiencing the largest decreases in intensity after the addition of **oligo-HP4.2** at the concentration of 25 µM to the protein’s solution (1:0.25 protein/RNA molar ratio) correspond to residues located in the β-platform as well as in in loop-1 (β_1_-α_2_) and loop-3 (β_2_-β_3_) of both RRMs (**Figure S15, position A and B i and ii**) (Ser15, Met22, Ile24, Gly25, Gly26, Met52, Gly64, Ala95, Met104, Arg107, Thr108, Val113, Gly114, Gly115, Leu116, Ser117, Met139, Leu140, Phe142, Asp143, Thr146, Arg150, Gly153, Val155, Ser159, Glu164, Ala184, GLn185 and Val189). However, after the addition of RNA in a 1:1 ratio with respect to the protein (~ 100 μM, RNA) the signals of the new species do not increase further in intensity, and signals of the free protein seem to be still present.

The interaction of the double mutant tandem domain of Musashi with the newly designed RNA strands, **oligo-L3.2** and **oligo-HP4.2**, has been evaluated with solution NMR. During both titration of MSI-1 RRM_1-2_ DM with **oligo-L3.2** and **oligo-HP4.2**, the cross-peaks of the free protein decrease in intensity while new cross-peaks, corresponding to the protein in complex with the RNA, appear and increase in intensity (**Figure 4**). The signals of the free proteins experiencing the largest decreases in intensity after the addition of **oligo-L3.2** and **oligo-HP4.2** at the concentration of 25 µM to the proteins solution (1:0.25 protein/RNA molar ratio) correspond to residues located on both RRMs (for MSI-1 RRM_1-2_ DM with **oligo-L3.2**: Asp18, Met22, Ile24, Gly26, Cys49, Val66, Val94, Ala95, Arg107, Ile111, Gly114, Gly115, Phe132, Met139, Asp143, Thr146 and Asn147, **Figure S13 panel A and B iv**; for MSI-1 RRM_1-2_ DM with **oligo-HP4.2**: Ser15, Lys21, Met22, Ile24, Gly25, Gly26, Leu50, Met52, Arg61, Gly64, Phe68, Asp91, Lys93, Val94, Ala95, Lys103, Arg107, Val113, Met141, Val155, Ala184, Gln185, Lys187, Val189 and Met190, **Figure S15 panel A and B iv**).

## Kinetic evaluation of UAG and CAG motif for the isolated wild-type and modified RRM2 domain

**Oligo-L2.1** binding motif was engineered to modify RRMs binding likelihood, and subsequently a difference in affinity is expected. Both RRM1 and RRM2 had similar association and dissociation rate constants, and therefore similar affinity values. The RRM1 association rate constant was similar to the one obtained with **oligo-L2**, while the dissociation rate constant was 2-fold faster, resulting in a 3-fold weaker affinity. RRM2 binding to **oligo-L2.1** displayed a two-fold slower association rate constant and a two-fold faster dissociation rate constant, leading to an almost 5-fold weaker affinity value (**Figure S4, Table 2**).

RRM2-M1 binding to **oligo-L2.1** showed an almost 5-fold faster association rate constant, with no affection on the dissociation rate constant compared with RRM2. This produced a 3-fold stronger affinity for RRM2-M1 compared with RRM2. RRM2-M2 displayed a similar association rate constant and a slightly slower dissociation rate constant, leading to a less than two-fold stronger affinity. Both RRM2-M1 and M2 displayed faster association and dissociation rate constants than RRM2. RRM2-M1 association rate constant was 10-fold faster, while the dissociation rate constant was almost 10-fold slower. RRM2-M2 showed a similar profile to RRM2 with less than 3-fold faster association and 3-fold slower dissociation. Affinities for RRM2-M1 and M2 were respectively 58.6 nM and 62.9 nM (**Figure S4, Table 2**).

**Oligo-L3.2** presented a substitution in one of the UAG motifs, which turned to CAG. Both RRM1 and RRM2 had similar dissociation rate constants with a two-fold difference in the association rate constant, being RRM2 faster than RRM1. Therefore, there was an almost two-fold stronger affinity of RRM2 compared with RRM1. Regarding, MSI-1 RRM_1-2_, the UAG change for CAG was reflected in the monovalent affinity, with a 6-fold slower association than **oligo-L3** and a similar dissociation rate for both RRM_1-2_ and RRM_1-2_ DM. They showed a similar kinetic profile and affinity values when interacting with **oligo-L3.2**, including the bivalent affinity, that is not affected (**Figure S4, Table 2**).

# Suppl. Tables and Figures

#

|  | MSI-1 RRM1 | MSI-1 RRM2 | Oligo  L2 | Oligo  L3 | OligoHP4 | Oligo  HP5 |
| --- | --- | --- | --- | --- | --- | --- |
| dn/dc (ml/g) | 0.185 | 0.185 | 0.180 | 0.180 | 0.180 | 0.180 |
| UV extinction coefficient (ml/(mg·cm)) | 1.045 | 0.586 | 6.200 | 6.200 | 6.200 | 6.200 |
| Theoretical MW (KDa) | 13.491 | 12.133 | 4.975 | 5.023 | 7.287 | 8.245 |

**Table S1.** Size Exclusion Chromatography with Dynamic Light Scattering parameters of MSI-1 RRM1, RRM2 and oligos.

**
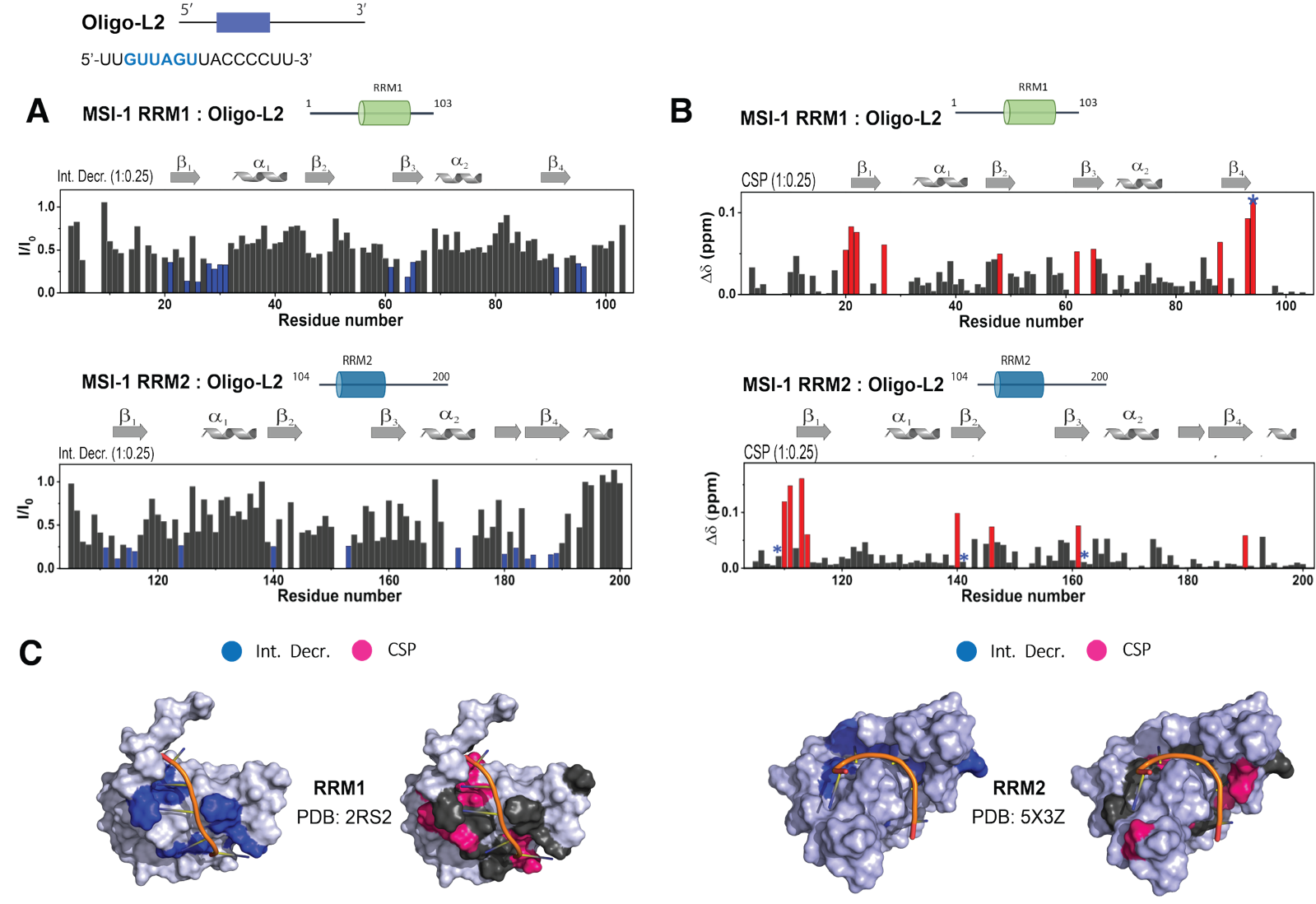
**

**Figure S1.** Interaction of the isolated (RRM1 and RRM2) with **oligoL2**. (A) Plot of the decreases in intensity of the signals of the isolated RRM1 and RRM2 domains. The residues experiencing the largest changes during the interaction with **oligo-L2** at a protein: RNA molar ratio of 1:0.25 have been highlighted in blue (RRM1: Lys21, Ile24, Gly26, Ser28, Trp29, Gln30, Thr31, Arg61, Gly64, Phe65, Asp91, Ala95 and Phe96; RRM2: Ile111, Val113, Gly115, Leu116, Asp124, Leu140, Gly153, his172, Glu180, Lys182, A184, Gln185, Glu188 and Val189). (B) Plot of the Chemical Shift Perturbation (CSP) of the signals of the isolated RRM1 and RRM2 domains. The residues experiencing the largest changes during the interaction with **oligo-L2** at the protein: RNA molar ratio of 1:1 have been highlighted in red (RRM1: Lys21, Met22, Gln30, Thr31, Arg61, Gly64, Lys93 and Val 94: RRM2: Lys110, Ile111, Val113, Gly114, Leu140, Thr146, Asp161 and Met150). Residues not able to assign (RRM1: Phe23, Ile24, Gly26, Trp29, Glu30, Thr31, Leu56, Thr89, Asp91, Ala95, Phe98 and Ala100; RRM2: Thr108, Gly115, Leu116, Vl118, Met139, Gly153, His172, Lys182, Ala184, Glu188 and Val189). Residues with ambiguity in the assignment have been pointed at with a blue star. (C) Mapping of the residues experiencing the largest effect in each RRM on the structure (PDB:2RS2 for RRM1 and PDB: 5X3Z for RRM2). Highlighted in blue the residues experiencing the largest intensity decrease and in pink the ones experiencing the largest chemical shift perturbation.

**
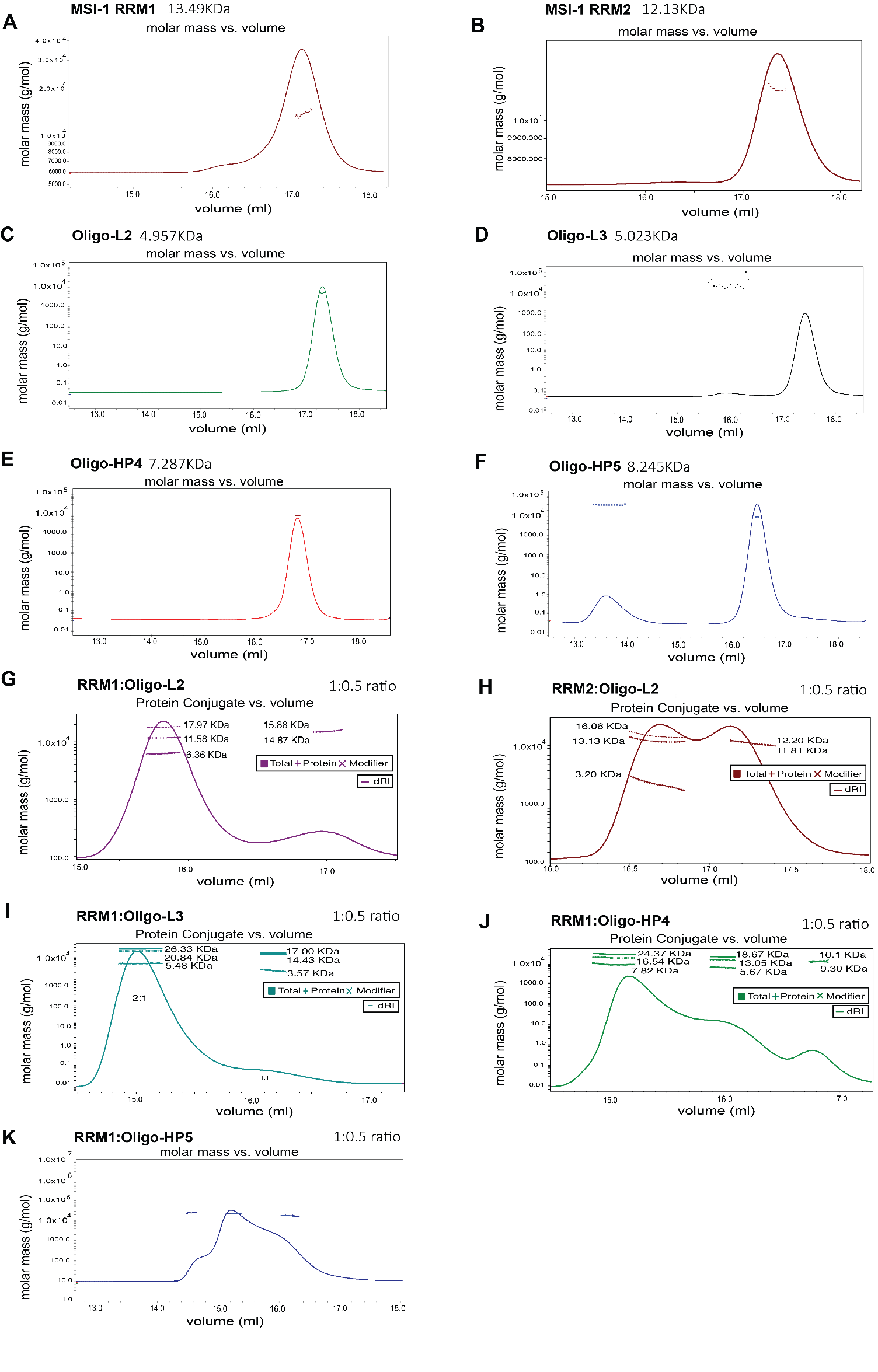
**

**Figure S2.** SEC-MALS chromatograms and protein Conjugation analysis. (A) Chromatogram of the MSI-1 RRM1. Analysis of the peak corresponds to a molecular weight (MW) value of 13.86 kDa close to the theoretical value of 13.49 kDa. The peak corresponds to the monomeric form of the protein. (B) Chromatogram of the MSI-1 RRM2. Analysis of the peak corresponds to a MW value of 12.16 kDa similar to its theoretical MW of 12.49 kDa, and therefore, corresponding to the monomeric form of the protein. (C) Chromatogram of **oligo-L2**. Analysis shows one single peak with a MW of 5.14 kDa, close to the theoretical value of 4.95 kDa. Therefore, it shows the monomeric form of the RNA. (D) Chromatogram of **oligo-L3**. Analysis shows two peaks, one with really weak intensity of 16 kDa corresponding to some aggregate form of the RNA, and a second peak with much higher intensity with a MW of 5.12 kDa. The theoretical MW of this RNA is 5.023 kDa which indicates this second peak to be the monomeric form of **oligo-L3**. (E) Chromatogram of **oligo-HP4**. Analysis of the peak corresponds to a MW value of 7.911 kDa similar to its theoretical MW of 7.287 kDa, and therefore, corresponding to the monomeric form of **oligo-HP4**. (F) Chromatogram of **oligo-HP5**. Analysis shows two clear peaks, one of 38.10 kDa corresponding to some aggregate form of the RNA, and a second peak with much higher intensity with a MW of 8.77 kDa. The theoretical MW of this RNA is 8.245 kDa which indicates this second peak to be the monomeric form of **oligo-HP5**. (G) SEC –MALS Protein Conjugate analysis of the isolated RRM1 with **oligo-L2** at protein/RNA molar ratio of 1:0.50. Binding observed in a 1:1 stoichiometry. (H) SEC –MALS Protein Conjugate analysis of the isolated RRM2 with **oligo-L2** at protein/RNA molar ratio of 1:0.50. Binding observed in a 1:1 stoichiometry. (I) SEC–MALS Protein Conjugate analysis of the isolated RRM1 with **oligo-L3** at protein/RNA molar ratio of 1:0.50. Binding observed in both RRMs in 1:2 and 1:1 stoichiometry. (J) SEC –MALS Protein Conjugate analysis of the isolated RRM1 with **oligo-HP4** at protein/RNA molar ratio of 1:0.50. Several species of different stoichiometric ratios are present in solution. (K) Chromatogram of the isolated RRM1 with **oligo-HP5** at protein/RNA molar ratio of 1:0.50. Many species of different stoichiometric ratios are present in solution.

**
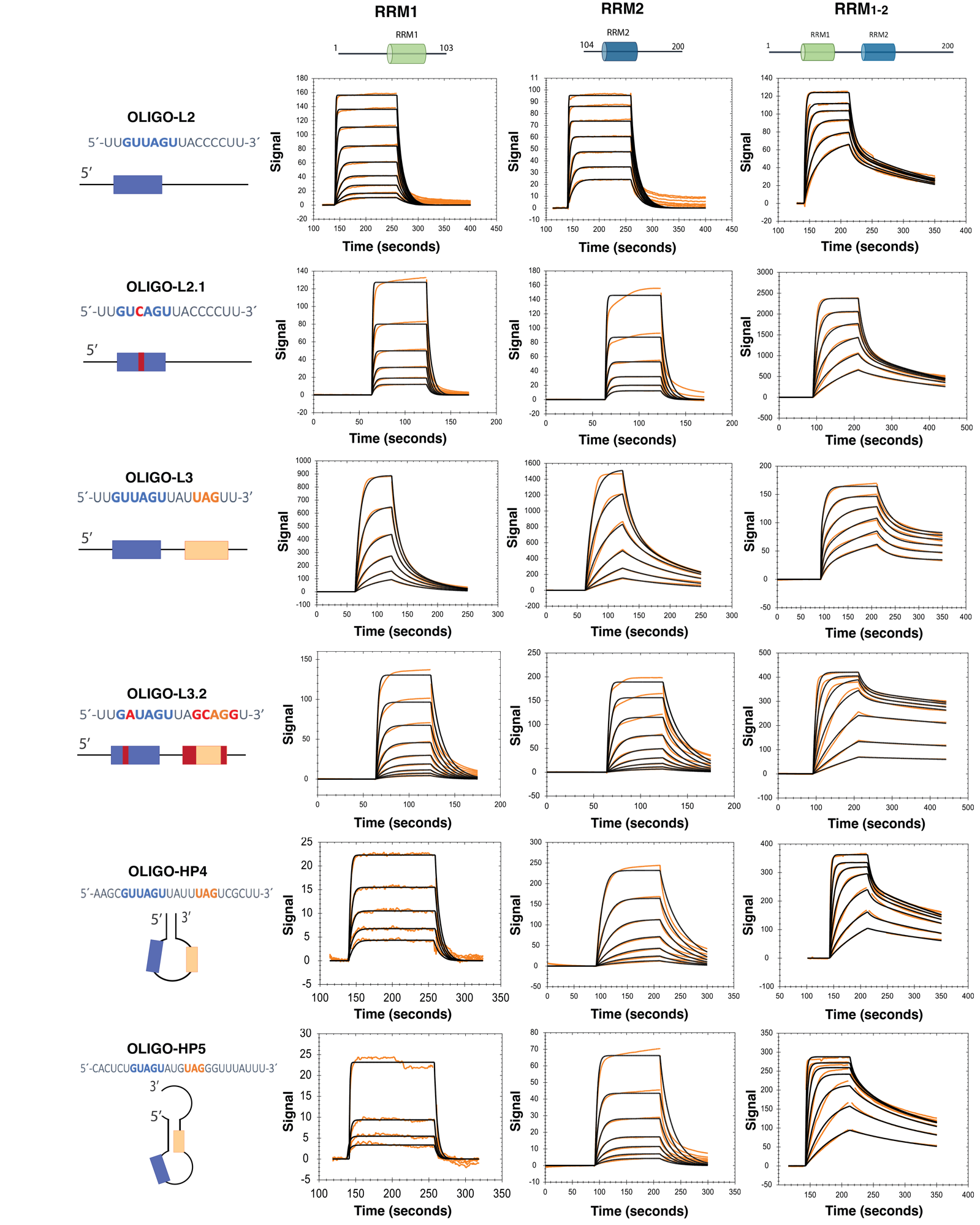
**

**Figure S3.** Sensorgrams from RRM1, RRM2 and MSI-1 RRM_1-2_ interacting with **oligo-L2, L2.1, L3, L3.2, HP4** and **HP5**, RRM1 and RRM2 interactions were fitted with a 1:1 model, while MSI-1 RRM_1-2_ were fitted with a 1:2 model. The fitted line is shown in black. The concentration ranges for each interaction were: RRM1 with **oligo-L2** 1.9 to 500 nM; RRM2 with **oligo-L2** 7.8 to 500 nM; RRM1 and RRM2 with **oligo-L2.1** 15.65 to 500 nM; RRM1 and RRM2 with **oligo-L3** 15.65 to 500 nM; RRM1 and RRM2 with **oligo-L3.2** 1.9 to 500 nM; RRM1 with **oligo-HP4** 3.9 to 62.5 nM; RRM1 with **oligo-HP5** 7.8, 15.65, 31.25 and 62.5 nM; RRM2 with **oligos-HP4** and **-HP5** 1.9 to 125 nM; MSI-1 RRM_1-2_ with **oligo-L2** 7.8 to 500 nM; MSI-1 RRM_1-2_ with **oligo-L2.1** 7.8 nM to 250 nM; MSI-1 RRM_1-2_ with **oligo-L3** 3.9 to 125 nM; MSI-1 RRM_1-2_ with **oligo-L3.2** 3.9 to 250 nM, and MSI-1 RRM_1-2_ with **oligos-HP4** and **-HP5** 1.9 to 500 nM.

**
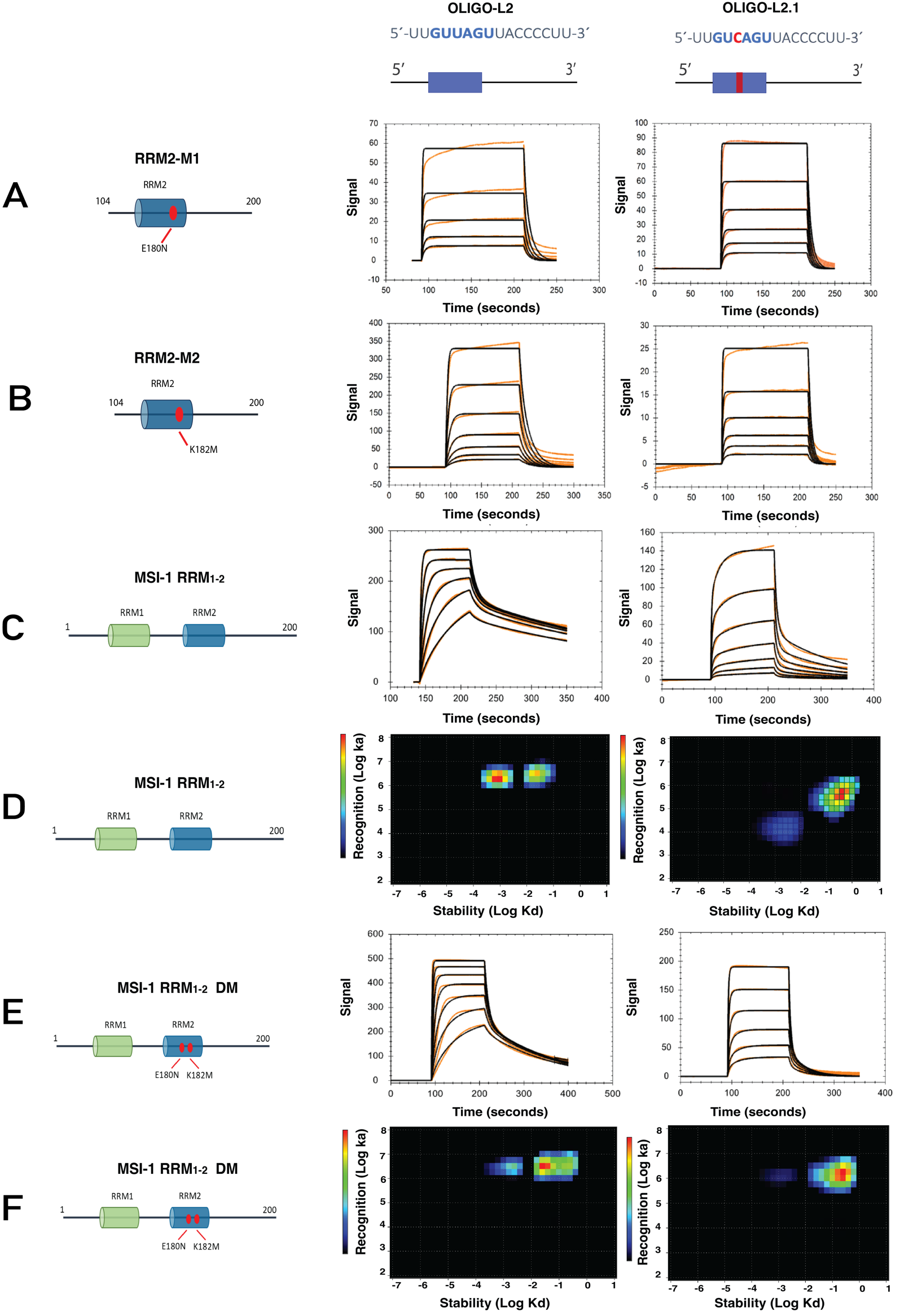
**

**Figure S4.** (A) Sensorgrams corresponding to RRM2-M1 interaction with **oligo-L2** with conc. range from 15.65 to 250 nM and **oligo-L2.1** with conc. range from 15.65 to 500 nM. (B) Sensorgrams corresponding to RRM2-M2 interaction with **oligo-L2** with conc. range ranging from 7.8 to 500 nM and with **oligo-L2.1** with conc. range from 15.65 to 500 nM. (C) Sensorgrams and (D) InteractionsMaps corresponding to interactions of MSI-1 RRM_1-2_ with **oligo-L2** with concentrations from 7.8 to 250 nM and with **oligo-L2.1** with conc. ranging from 7.8 to 500 nM. (E) Sensorgrams and (F) InteractionsMaps corresponding to interactions MSI-1 RRM_1-2_ DM with **oligo-L2** with conc. ranging from 7.8 to 500 nM and with **oligo-L2.1** with conc. ranging from 7.8 to 250 nM

**
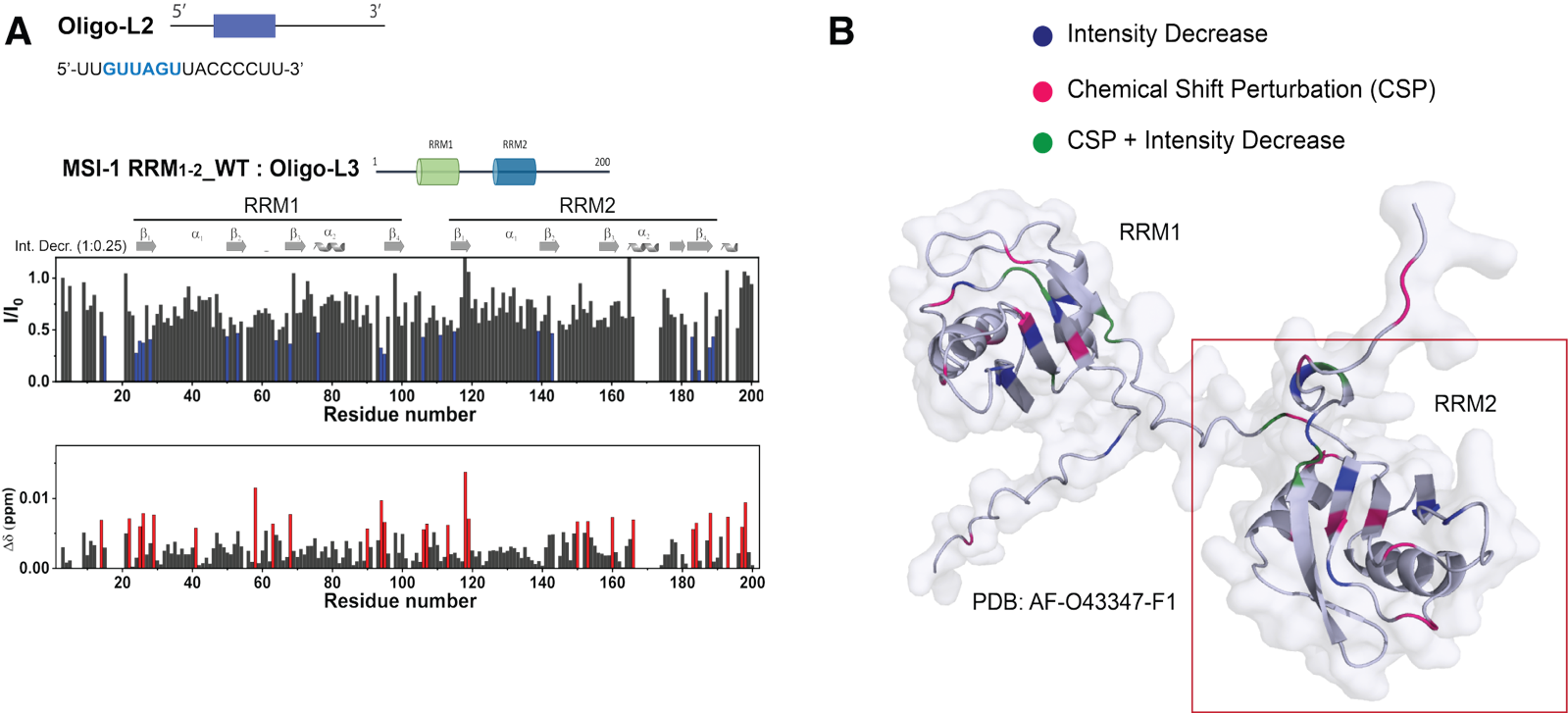
**

**Figure S5.** Interaction of MSI-1 RRM_1-2_ with **oligo-L2**. (A) On top, plot of the decreases in intensity of the signals of MSI-1 RRM_1-2_ protein. The residues experiencing the largest changes during the interaction with **oligo-L2** at a protein: RNA molar ratio of 1:0.25 have been highlighted in blue (at 1:0.25 protein/RNA molar ratio, mostly affected residues are: Ser15, Ile24-Gly26, Ser28, Leu50, Arg53, Gly64, Phe68, Lys76, Val94-Ala95, Thr106, Ile111, Gly115, Met139, Asp143, Lys183, Gln185 and Glu188-Val189). Below, plot of the Chemical Shift Perturbation (CSP) of the signals of the MSI-1 RRM_1-2_ protein. The residues experiencing the largest changes during the interaction with **oligo-L2** at the protein: RNA molar ratio of 1:0.25 have been highlighted in red (Asp14, Met22, Gly25-Gly26, Trp29, Gly41, Lys58, Phe63, Phe68, Ile90, Val94-Ala95, Thr106-Arg107, Val113, Val118-Asn119, Arg150, Gly153, Glu160, Val166, Lys183-Ala184, Glu188, Thr193 and Arg197-Gly198). (B) Mapping of the residues experiencing the largest effect on the RRM_1-2_ structure (Alpha fold model) at molar ratio 1:0.25. Highlighted in blue the residues experiencing the largest Intensity decrease, in pink the one experiencing the largest CSP, and in green the ones experiencing the largest effect in both Intensity decrease and CSP.

**
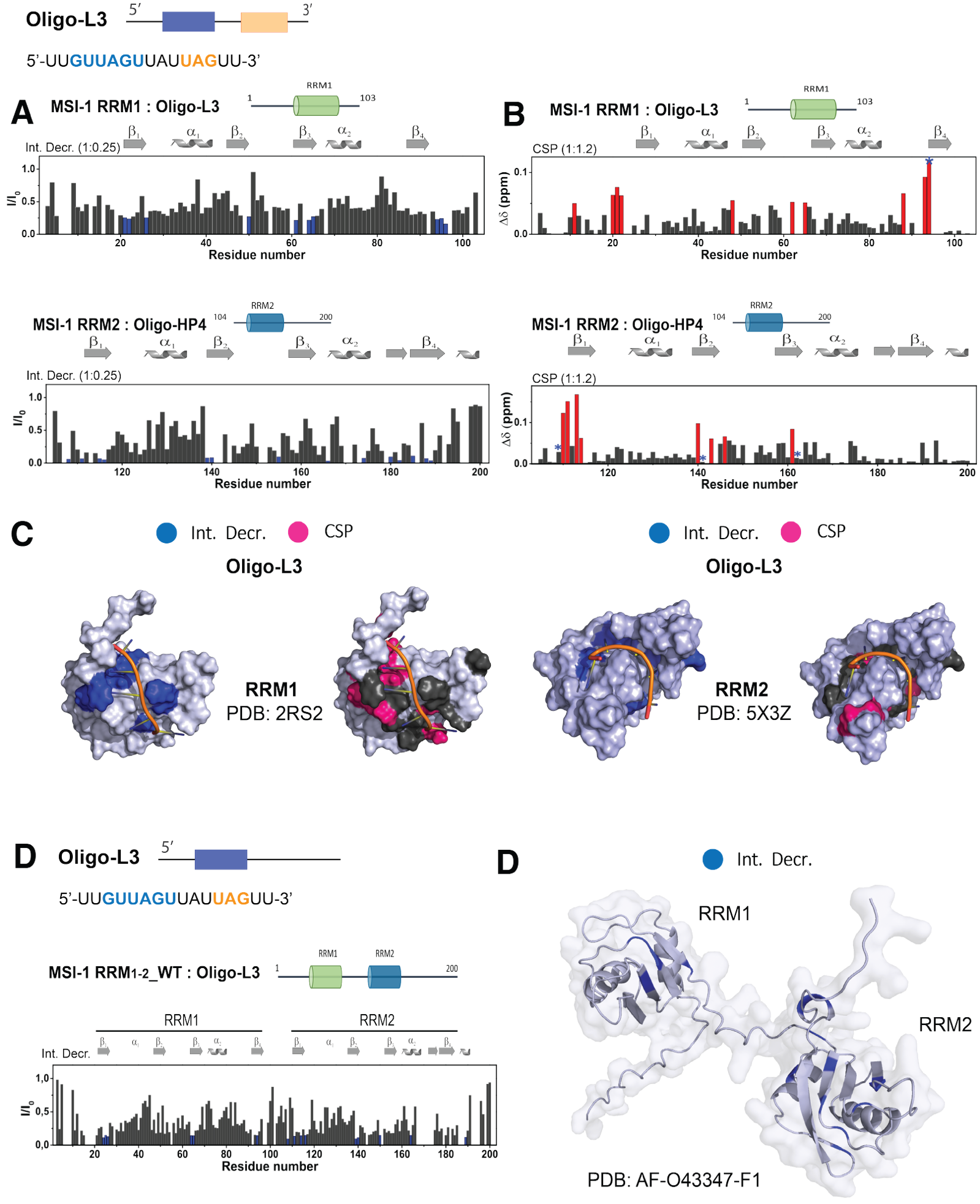
**

**Figure S6.**  Interaction of RRM1, RRM2 and MSI-1 RRM_1-2_ with **oligo-L3**. (A) Plot of the decreases in intensity of the signals of the isolated RRM1 and RRM2 domains. The residues experiencing the largest changes during the interaction with **oligo-L3** at a protein: RNA molar ratio of 1:0.25 have been highlighted in blue (RRM1: Lys21, Met22, Gly26, Leu50, Arg61, Gly64, Phe65, Val94, Ala95 and Phe96; RRM2: Thr108, Ile111, Gly115, Leu116, Met139, Leu140, Val155, Val166, Ile174, Glu180, Lys182, Gln185, Glu188 and Val189). (B) Plot of the Chemical Shift Perturbation (CSP) of the signals of the isolated RRM1 and RRM2 domains. The residues experiencing the largest changes during the interaction with **oligo-L3** at the protein: RNA molar ratio of 1:1 have been highlighted in red (RRM1: Lys21, Gln30, Thr31, Arg61, Gly64, Lys93, Val94 and Ala100; RRM2: Lys110, Ile111, Val113, Gly114, Leu140, Asp143, Thr146 and Asp161; residues with ambiguity in the assignment have been pointed at with a blue star). Residues not able to assign (RRM1: Phe23, Ile24, Trp29, Thr89, Asp91, Ala95 and Phe96; RRM2: Arg107, Thr108, Gly115, Leu116, Met139, Met141, Gly152, Val155, His172, Glu180, Lys182, Ala184, Glu188, Val189). (C) Mapping of the residues experiencing the largest effect in each RRM on the structure (PDB:2RS2 for RRM1 and PDB: 5X3Z for RRM2). Highlighted in blue the residues experiencing a larger Intensity decrease and in pink the residues experiencing a larger chemical shift perturbation. (D) Plot of the decreases in intensity of the signals of MSI-1 RRM_1-2_ protein. The residues experiencing the largest changes during the interaction with **oligo-L3** at a protein: RNA molar ratio of 1:0.25 have been highlighted in blue (Lys21, Met22, Gly26, Leu50, Arg61, Gly64, Phe65, Val94, Ala95 and Phe96, Thr108, Ile111, Gly115, Leu116, Met139, Leu140, Val155, Val166, Ile174, Glu180, Lys182, Gln185, Glu188 and Val189). (E) Mapping of the residues experiencing a larger intensity decrease effect on the RRM_1-2_ structure (Alpha fold model) at molar ratio 1:0.25. Highlighted in blue the residues experiencing a larger Intensity decrease.

**
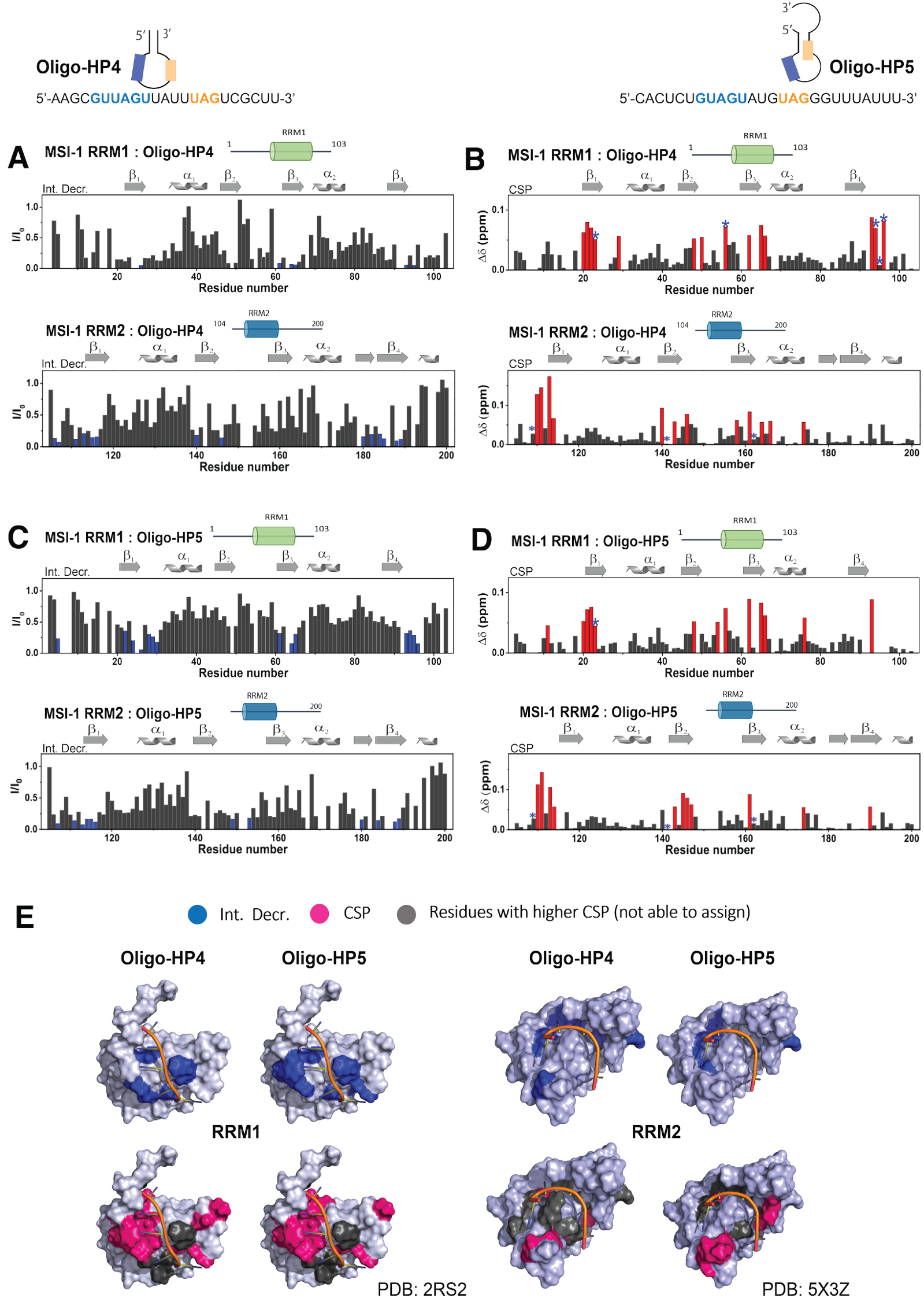
**

**Figure S7.** Interaction of RRM1 and RRM2 with **oligo-HP4** and **oligo-HP5**. (A) Plot of the decreases in intensity of the signals of the isolated RRM1 and RRM2 domains. The residues experiencing the largest changes during the interaction with **oligo-HP4** at a protein: RNA molar ratio of 1:0.25 have been highlighted in blue (RRM1: Ile24, Gly26, Ser60, Arg61, Gly64, Phe65, Asp91, Lys93 and Ala95; and RRM2: Thr106, Arg107, Ile111, Val113, Gly115, Leu116, Leu140, Thr146, Glu180, Lys182, Ala184, Gln185, Glu188 and Val189). (B) Plot of the Chemical Shift Perturbation (CSP) of the signals of the isolated RRM1 and RRM2 domains. The residues experiencing the largest changes during the interaction with **oligo-HP4** at the protein: RNA molar ratio of 1:1 have been highlighted in red (RRM1: Cys20, Lys21, Met22, Phe23, Trp29, Thr31, Glu48, Leu50, Leu56, Gly62, Phe65, Val66, Lys93, Val94 and Phe96; and RRM2: Lys110, Ile111, Val113, Gly114, Leu140, Asp143, Thr146, Glu158, Asp161, Glu164, Val166 and Ile174; residues with ambiguity in the assignment have been pointed at with a blue star). Unfortunately, many signals could not be reassigned because of their too large CSP (RRM1: Ile24, Gly26, Gln30, Ser60, Arg61, Gly64, Thr89 and Ala95; and RRM2: Arg107, Thr108, Gly115, Leu116, Val118, Met139, Met141, Arg150, Gly153, Val155, His172, Lys182, Ala184, Glu188 and Val189). (C) Plot of the decreases in intensity of the signals of the isolated RRM1 and RRM2 domains. The residues experiencing the largest changes during the interaction with **oligo-HP5** at a protein: RNA molar ratio of 1:0.25 have been highlighted in blue (RRM1: Ala5, Met22, Ile24, Gly26, Ser28, Trp29, Gln30, Arg61, Gly64, Phe65, Lys93, Val94, Ala95 and Phe96; and RRM2: Arg107, Ile111, Val113, Gly114, Gly115, Leu116, His149, Gly153, Glu180, Ala184, Glu188 and Val189). (D) Plot of the Chemical Shift Perturbation (CSP) of the signals of the isolated RRM1 and RRM2 domains. The residues experiencing the largest changes during the interaction with **oligo-HP5** at the protein: RNA molar ratio of 1:1 have been highlighted in red (RRM1: Ala11, Cys20, Lys21, Met22, Phe23, Glu48, Asp54, Leu56, Gly62, Phe65, Val66, Lys76 and Lys93; and RRM2: Lys110, Ile111, Val113, Gly114, Asp143, Thr145, Thr146, Asn147, Asp161, Ile174 and Met190; residues with ambiguity in the assignment have been pointed at with a blue star). Unfortunately, many signals could not be reassigned because of their too large CSP (RRM1: Ile24, Gly26, Trp29, Gln30, Ser60, Arg61, Gly64, Val94, Ala95 and Phe96; and RRM2: Arg107, Thr108, Gly115, Leu116, Met141, Gly153, Val155, His172, Glu180, Lys182, Ala184, Glu188 and Val189). (E) Mapping of the residues experiencing the largest effect in each RRM on the structure (PDB:2RS2 for RRM1 and PDB: 5X3Z for RRM2). Highlighted in blue the residues experiencing the largest Intensity decrease and in pink the one experiencing a larger CSP.

**
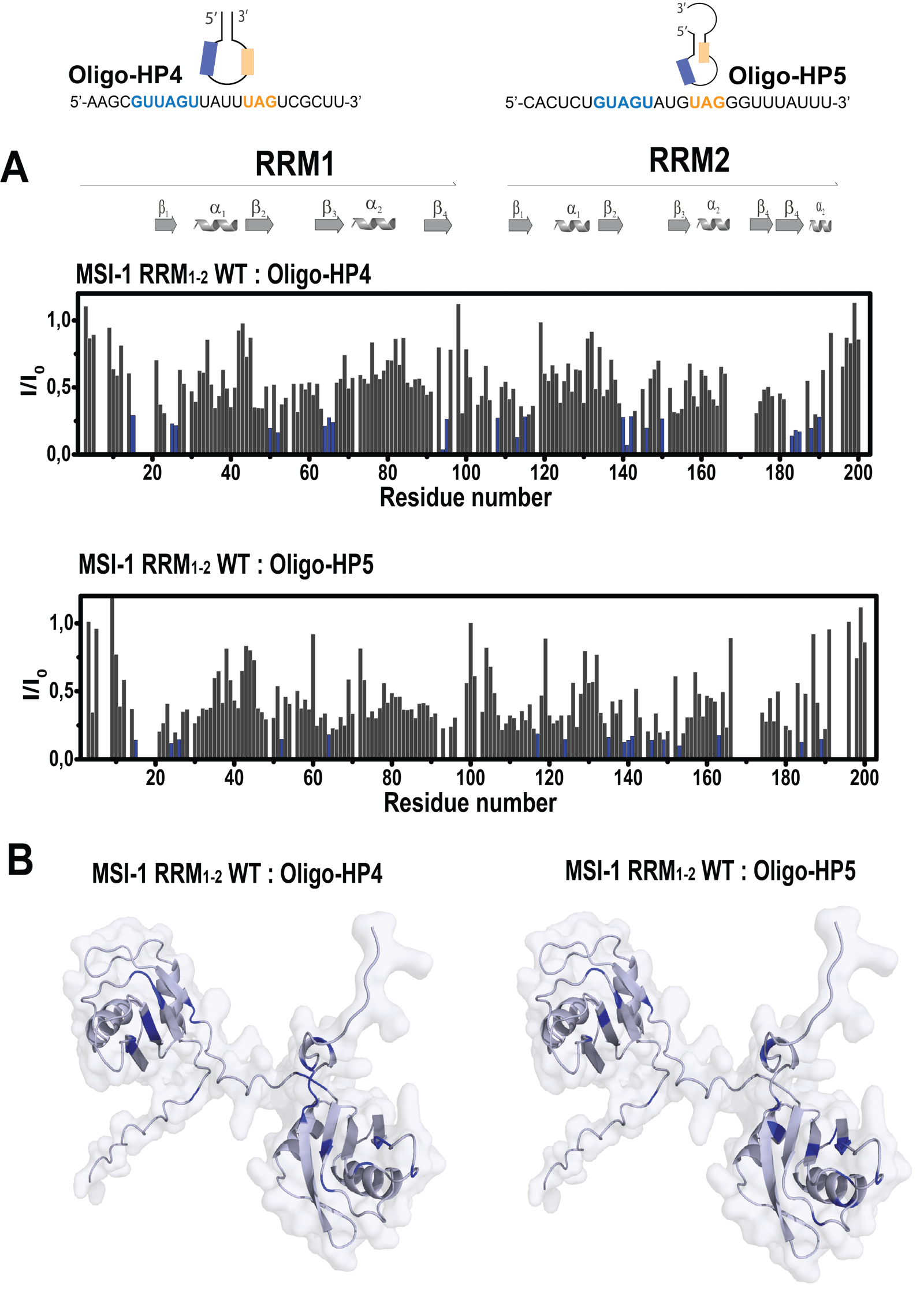
**

**Figure S8.** Interaction of RRM_1-2_ with **oligo-HP4** and **oligo-HP5**. (A) Plot of the decreases in intensity of the signals of the MSI-1 RRM_1-2_ protein. The residues experiencing the largest changes during the interaction with **oligo-HP4** and **oligo-HP5** at a protein: RNA molar ratio of 1:0.25 have been highlighted in blue. (B) Mapping of the residues experiencing the largest effect in the tandem domain protein on the structure (AF-O43347-F1) upon the binding. Highlighted in blue the residues experiencing the largest Intensity decrease.


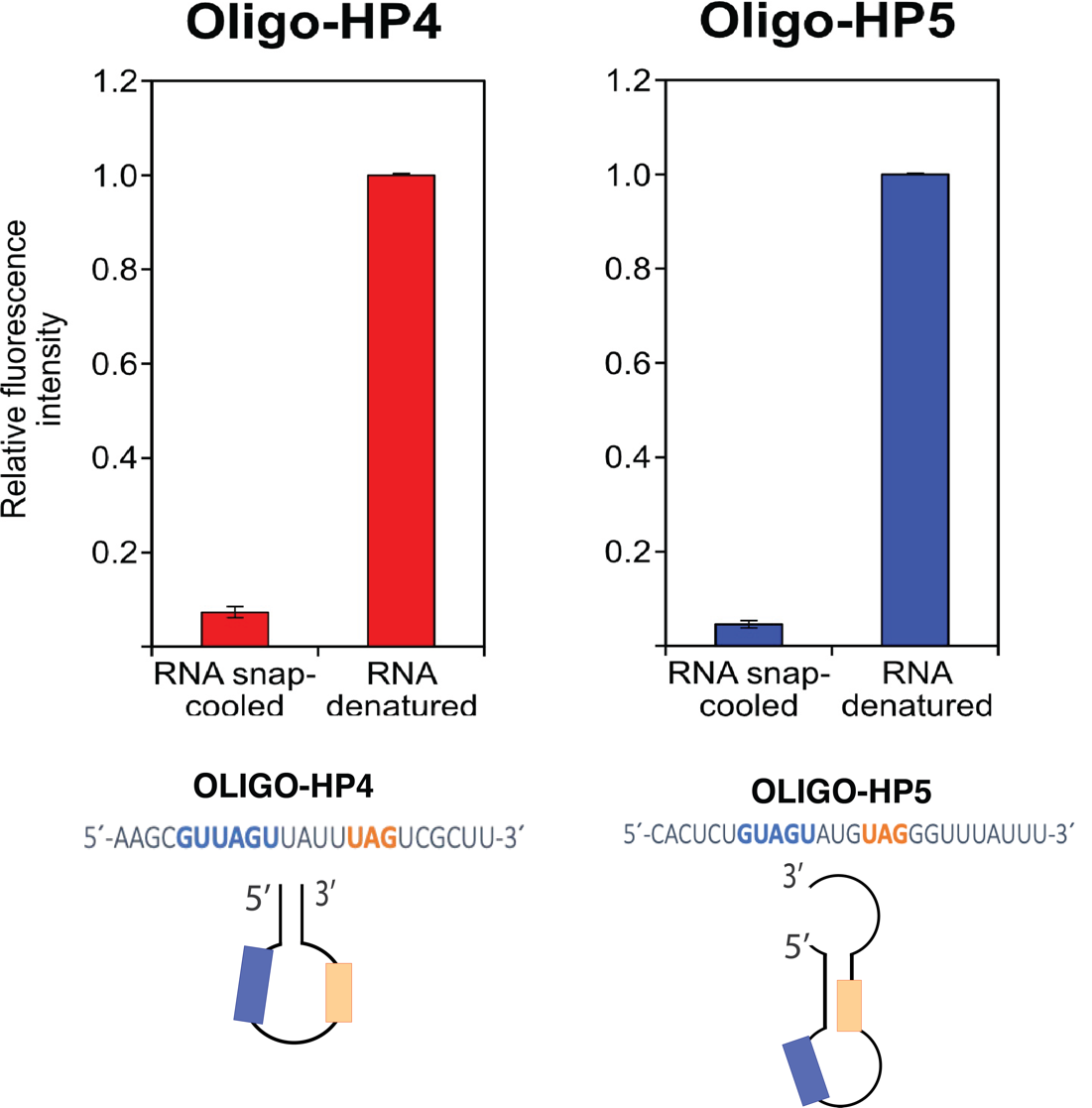


**Figure S9.** Control experiments for fluorescence quenching assays of **oligo-HP4** (in red) and **oligo-HP5** (in blue). The RNA folded state after snap cooling (low fluorescence intensity) and the unfolded/denatured state (high fluorescence intensity) were measured for both oligos. The fluorescence intensities are normalized respect to the RNA denatured state.

**
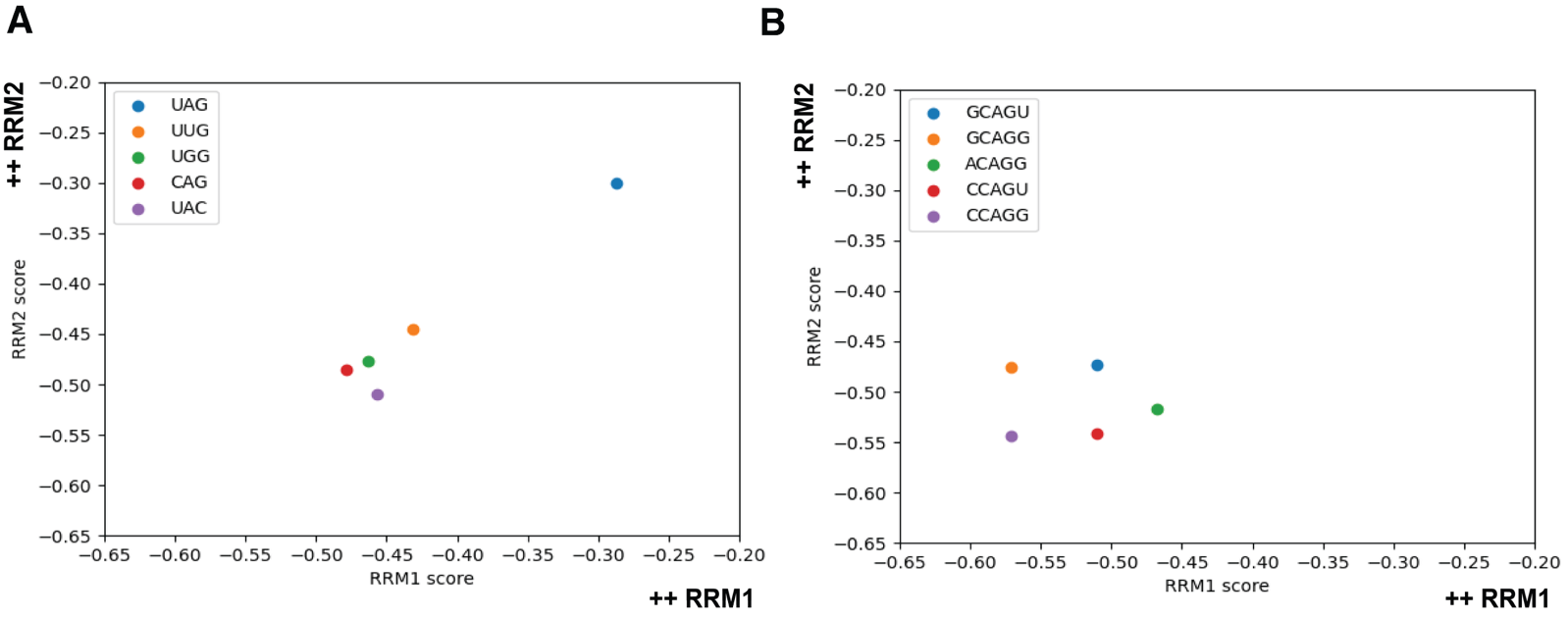
**

**Figure S10.** Scoring values from RRMScorer calculations. Scores are computed for each residue-nucleotide interacting position individually and the final score is normalized from 0 to 1. Low values indicate that the RRM-RNA complex does not show very favourable contacts while higher values indicate that the contacts are very often observed in the training data set of the multiple sequence alignment. (A) Top-scoring RNAs for the isolated domains. (B) RRMScorer values of 5 nucleotide mutations scored against RRM1 (x-axes) and RRM2 (y-axes) of MSI-1 protein.

**
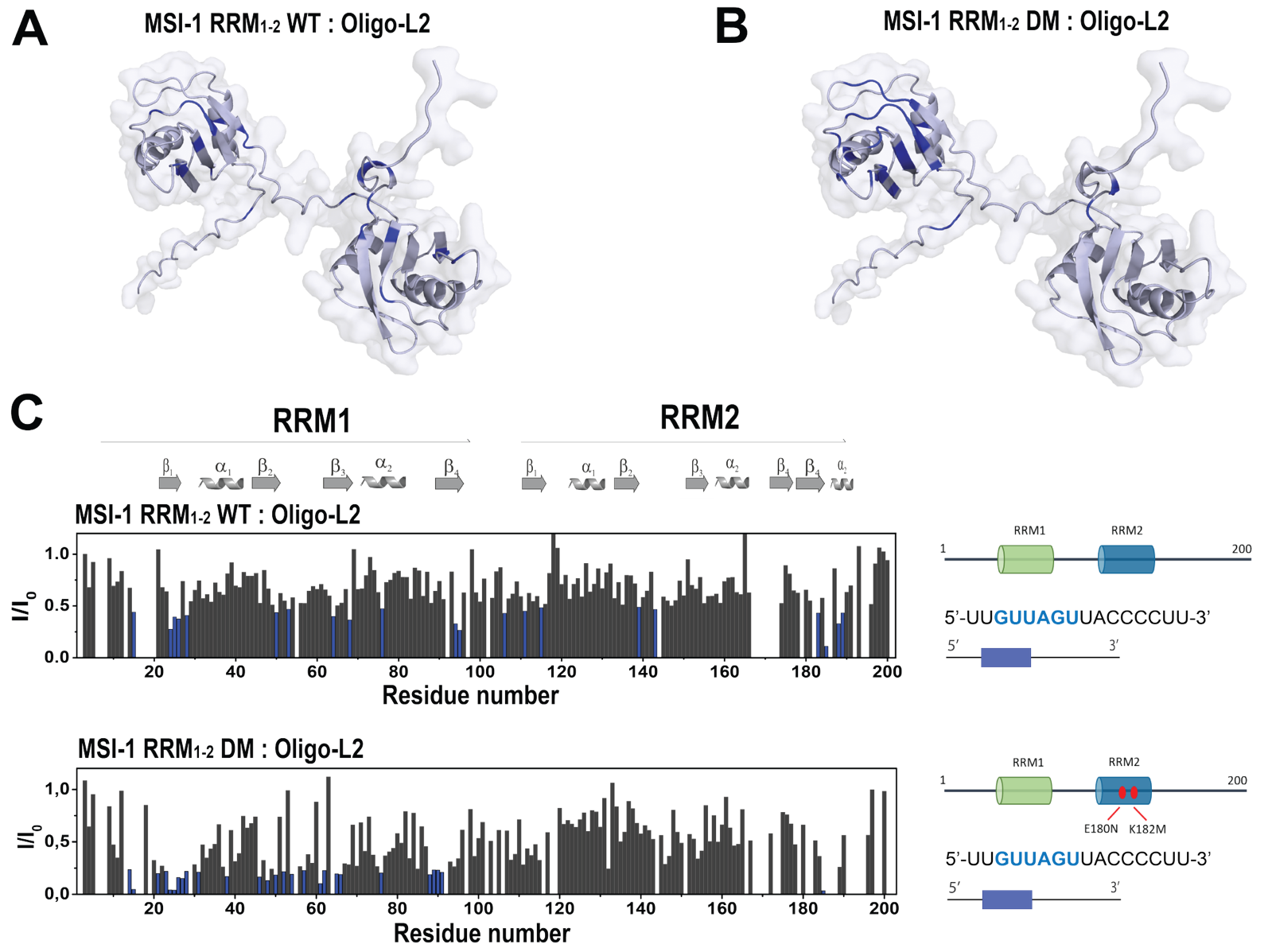
**

**Figure S11.** Comparison of interaction between MSI-1 RRM_1-2_ WT and MSI-1 RRM_1-2_ DM proteins with **oligo-L2**. (A) Mapping of the residues experiencing a larger effect in the wild-type tandem domain protein on the structure (AF-O43347-F1) upon the binding. Highlighted in blue the residues experiencing a larger Intensity decrease at a protein/RNA molar ratio of 1:0.25. (B) Mapping of the residues experiencing a larger effect in the DM tandem domain protein on the structure (AF-O43347-F1) upon the binding. Highlighted in blue the residues experiencing a larger Intensity decrease at a protein/RNA molar ratio of 1:0.25. (C) Graphic highlighting in blue on the WT and DM tandem domains the residues most affected by an intensity decrease effect during the interaction with **oligo-L2** at a molar ratio of protein/RNA of 1:0.25.

**
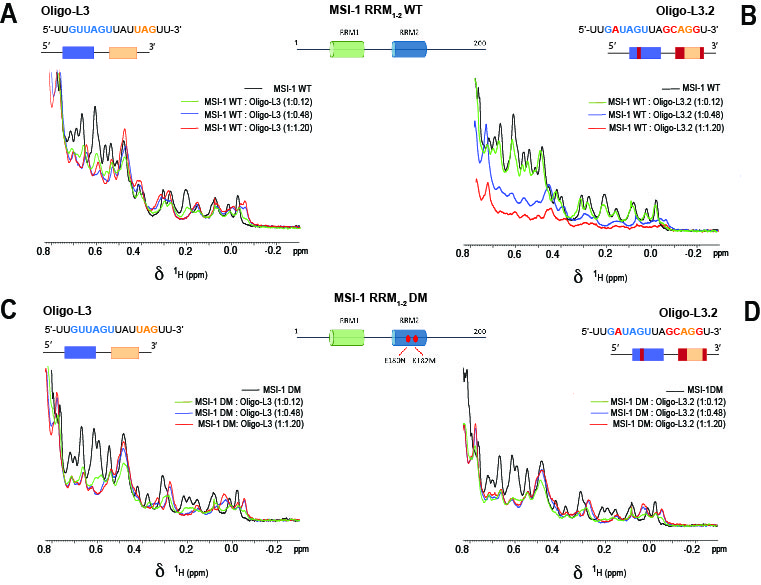
**

**Figure S12.** One-dimensional NMR analysis comparison of MSI-1 RRM_1-2_ WT and MSI-1 RRM_1-2_ DM proteins interaction with **oligo-L3** and **oligo-L3.2.** (A) One-dimensional NMR titration steps of MSI-1 RRM_1-2_ WT interaction with **oligo-L3**. (B) One-dimensional NMR titration steps of MSI-1 RRM_1-2_ WT interaction with **oligo-L3.2**. (C) One-dimensional NMR titration steps of MSI-1 RRM_1-2_ DM interaction with **oligo-L3**. (D) One-dimensional NMR titration steps of MSI-1 RRM_1-2_ DM interaction with **oligo-L3.2**.

**
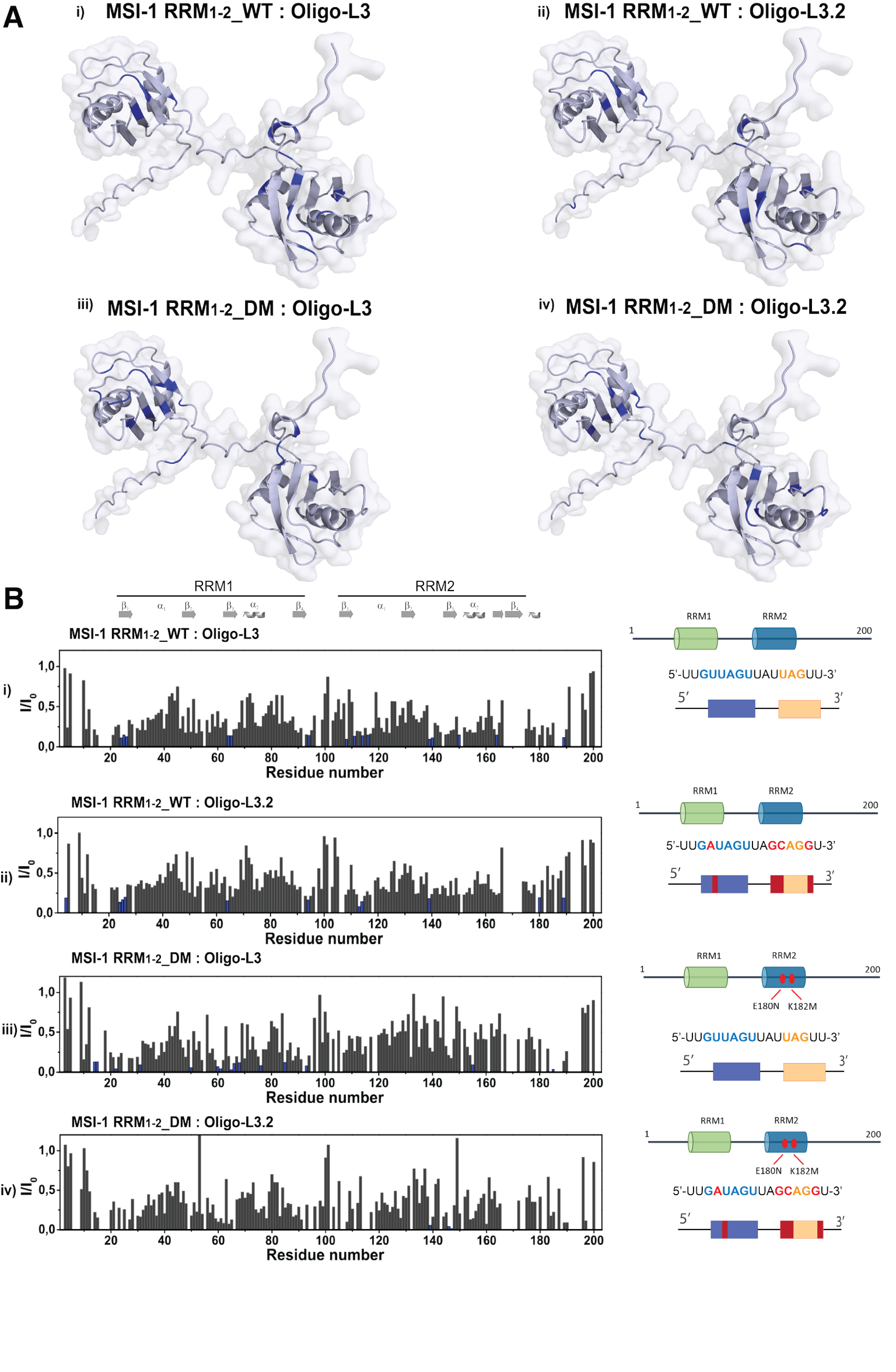
**

**Figure S13.** Comparison of interaction between MSI-1 RRM_1-2_ WT and MSI-1 RRM_1-2_ DM proteins with **oligo-L3** and **-L3.2**. (A) Mapping of the residues experiencing a larger effect in the wild-type and DM tandem domain protein on the structure (AF-O43347-F1) upon the binding with **oligo-L3** and **oligo-L3.2**. Highlighted in blue the residues experiencing a larger Intensity decrease at a protein/RNA molar ratio of 1:0.25. (B) Graphic highlighting in blue on the WT and DM tandem domains the residues mostly affected by an intensity decrease effect during the interaction with **oligo-L3** and **oligo-L3.2** at a molar ratio of protein/RNA of 1:0.25.

**
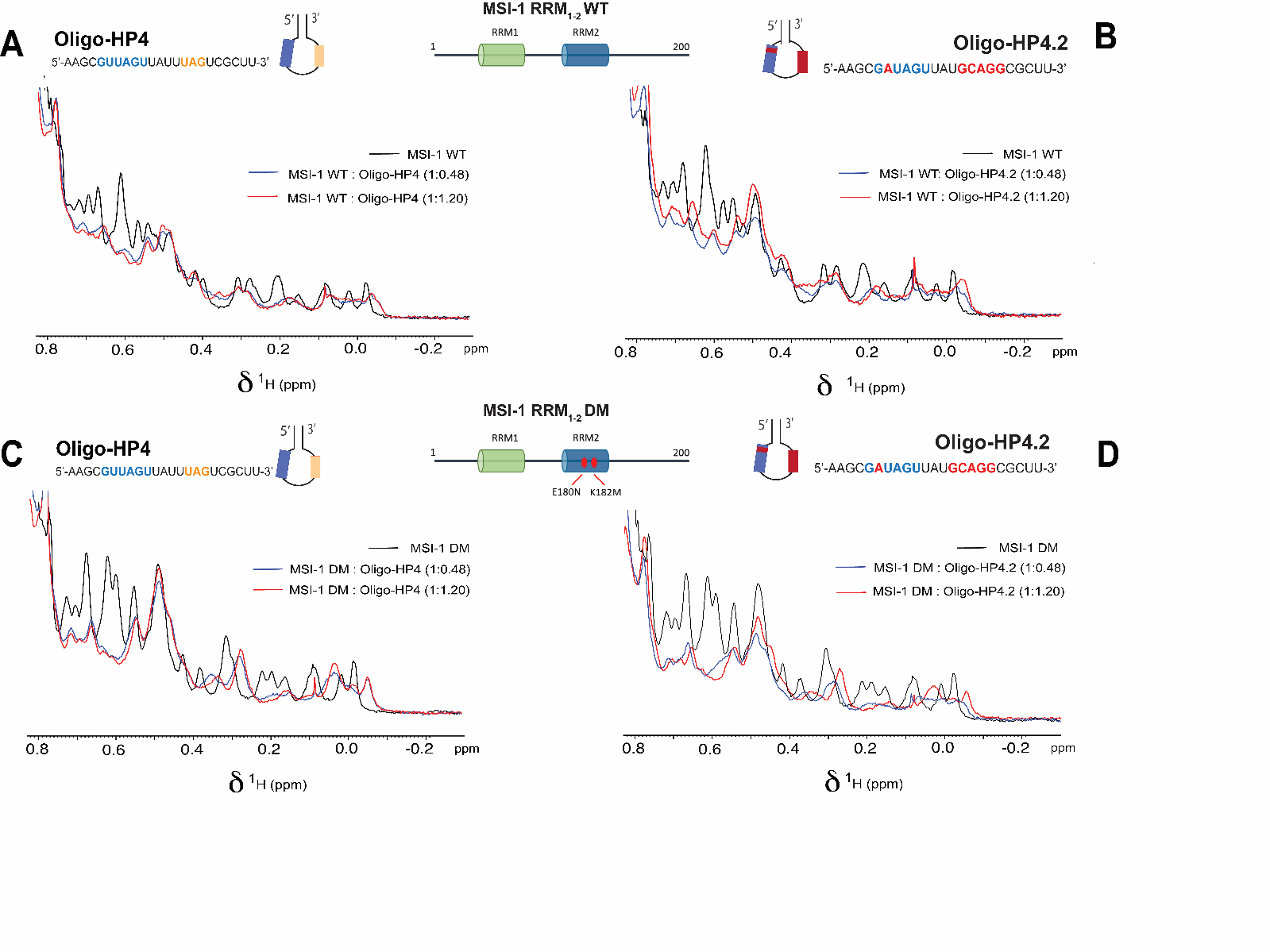
**

**Figure S14.** One-dimensional NMR analysis comparison of MSI-1 RRM_1-2_ WT and MSI-1 RRM_1-2_ DM proteins interaction with **oligo-HP4** and **oligo-HP4.2**. (A) One-dimensional NMR titration steps of MSI-1 RRM_1-2_ WT interaction with **oligo-HP4**. (B) One-dimensional NMR titration steps of MSI-1 RRM_1-2_ WT interaction with **oligo-HP4.2**. (C) One-dimensional NMR titration steps of MSI-1 RRM_1-2_ DM interaction with **oligo-HP4**. (D) One-dimensional NMR titration steps of MSI-1 RRM_1-2_ DM interaction with **oligo-HP4.2**.

**
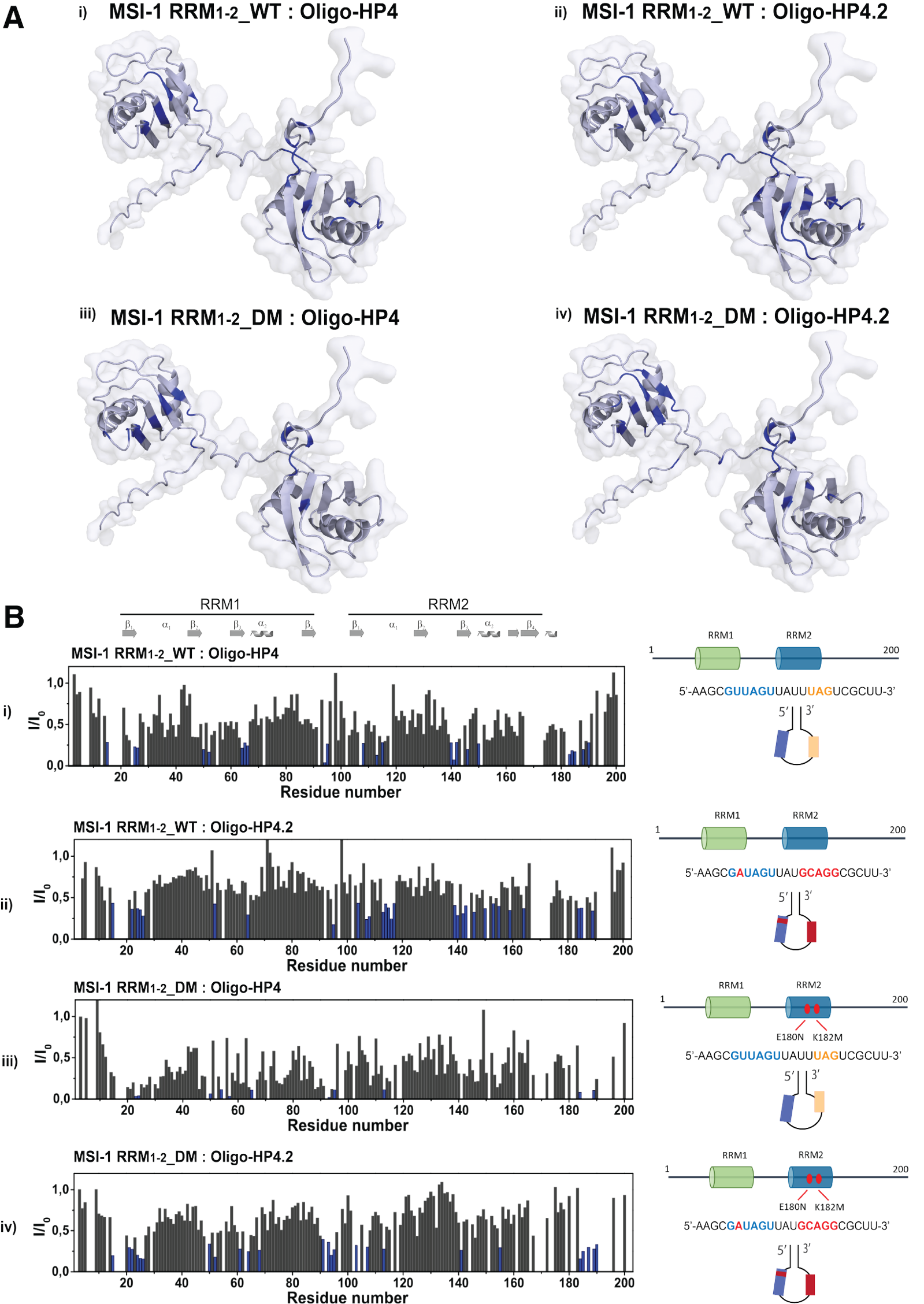
**

**Figure S15.** Comparison of interaction between MSI-1 RRM_1-2_ WT and MSI-1 RRM_1-2_ DM proteins with **oligo-HP4** and **-HP4.2**. (A) Mapping of the residues experiencing a larger effect in the Wild-type and DM tandem domain protein on the structure (AF-O43347-F1) upon the binding with **oligo-HP4** and **oligo-HP4.2**. Highlighted in blue the residues experiencing a larger Intensity decrease at a protein/RNA molar ratio of 1:0.25. (B) Graphic highlighting in blue on the WT and DM tandem domains the residues mostly affected by an intensity decrease effect during the interaction with **oligo-HP4** and **oligo-HP4.2** at a molar ratio of protein/RNA of 1:0.25.

**
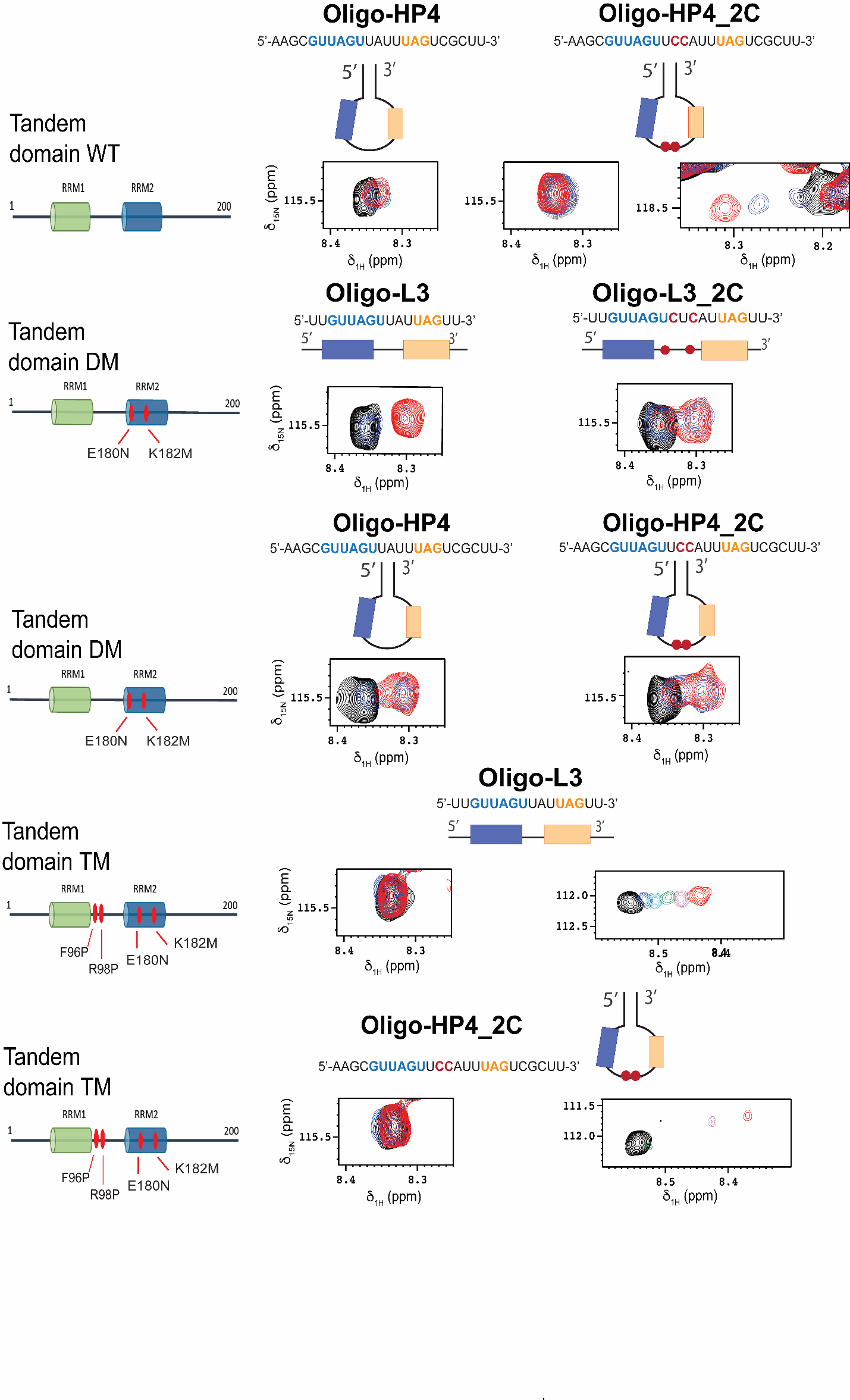
**

**Figure S16.** Zoom views of the 2D ^1^H-^15^N TROSY spectra, recorded on the on the tandem domain wild-type (WT), double mutant (DM: E180N, K182M) and tetra mutant (TM: F96P, R98P, E180N, K182M), respectively. In black are the spectra of the free proteins, in blue the spectra of the proteins in the presence of sub-stoichiometric concentrations of **oligo-L3**, **oligo-L3_2C**, **oligo-HP4** or **oligo-HP4_2C** (protein/RNA ratio of about 1:0.5), and in red the spectra of the proteins in the presence of **oligo-L3**, **oligo-L3_2C**, **oligo-HP4** or **oligo-HP4_2C** in the protein/RNA ratio of 1:1. The signal assigned to Asp-70 are displayed in the figure. Signal of the residue S191 and G114 from the RRM2 domain are also displayed for the interaction of **oligo-HP4_2C** with MSI-1 RRM_1-2_ WT and **oligo-L3** and **oligo-HP4_2C** with MSI-1 RRM_1-2_ TM, respectively.

**
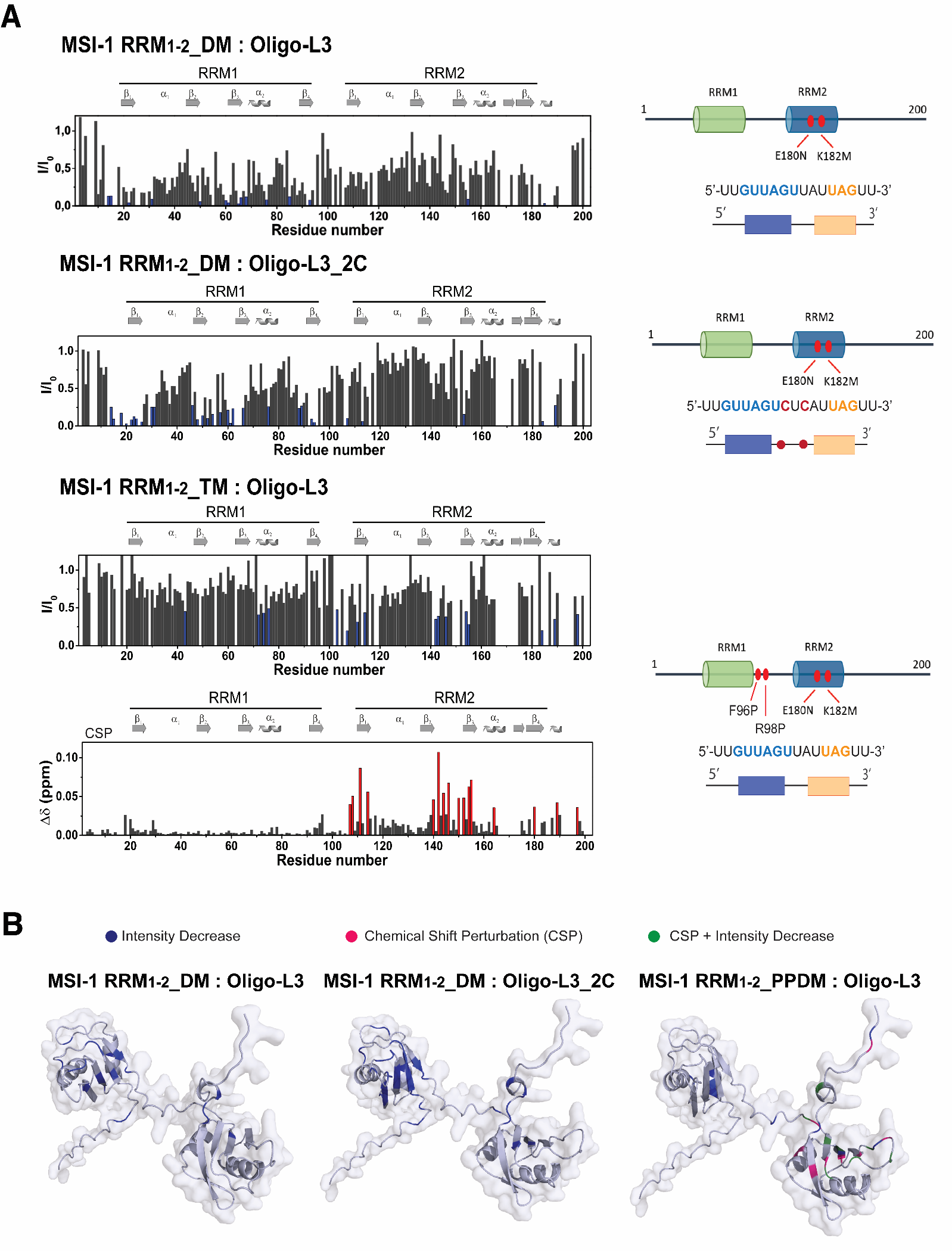
**

**Figure S17.** Interaction of MSI-1 RRM_1-2_ DM and MSI-1 RRM_1-2_ TM with **oligo-L3** and/or **oligo-L3_2C**. (A, top) Plot of the decreases in signal intensity of MSI-1 RRM_1-2_ DM with **oligo-L3** at a protein: RNA molar ratio of 1:0.25 (shown for comparison purposes). (A, middle) Plot of the decreases in signals intensity of MSI-1 RRM_1-2_ DM with **oligo-L3_2C**. The residues experiencing the largest changes during the interaction with **oligo-L3_2C** at the protein: RNA molar ratio of 1:0.25 have been highlighted in blue (Asp-14, Ser-15, Asp-18, Cys-20, Lys-21, Met-22, Phe-23, Ile-24, Gly-25, Gly-26, Gln-30, Thr-31, Val-46, Glu-48, Cys-49, Leu-50, Met-52, Asp-54, Thr-57, Ser-60, Arg-61, Gly-62, Gly-64, Phe-65, Val-66, Lys-76, Lys-88, Thr-89, Lys-93, Val-94, Ala-95, Arg-107, Val-113, Gly-153, Ala-184, Gln-185, Lys.187, Val-189). (A, bottom) Plot of the decreases in signals intensity and CSPs of MSI-1 RRM_1-2_-TM with **oligo-L3**. The residues experiencing the largest decreases in signal intensity during the interaction with **oligo-L3_2C** at the protein: RNA molar ratio of 1:0.25 have been highlighted in blue (Phe-43, Ala-72, Val-74, Lys-76, Lys-103, Arg-107, Ile-111, Gly-114, Phe-142, Asp-143, Thr-146, Arg-150, Gly-153, Phe-154, Val-155, Ala-184, Val-189, Gly-198), while the residues experiencing the largest chemical shift perturbation at a protein: RNA molar ratio of 1:1 have been highlighted in red (Arg-107, Thr-108, Ile-111, Gly-114, Leu-140, Phe-142, Lys-144, Thr-146, Arg-150, Phe-152, Phe-154, Val-155, Glu-164, Glu-180, Val-189, Arg-197). (B) Mapping of the residues experiencing the largest effect in the tandem domain protein on the structure (AF-O43347-F1) upon the binding. Highlighted in blue the residues experiencing the largest intensity decrease, in pink the residues experiencing the largest CSPs, and in green the residues experiencing both effects.


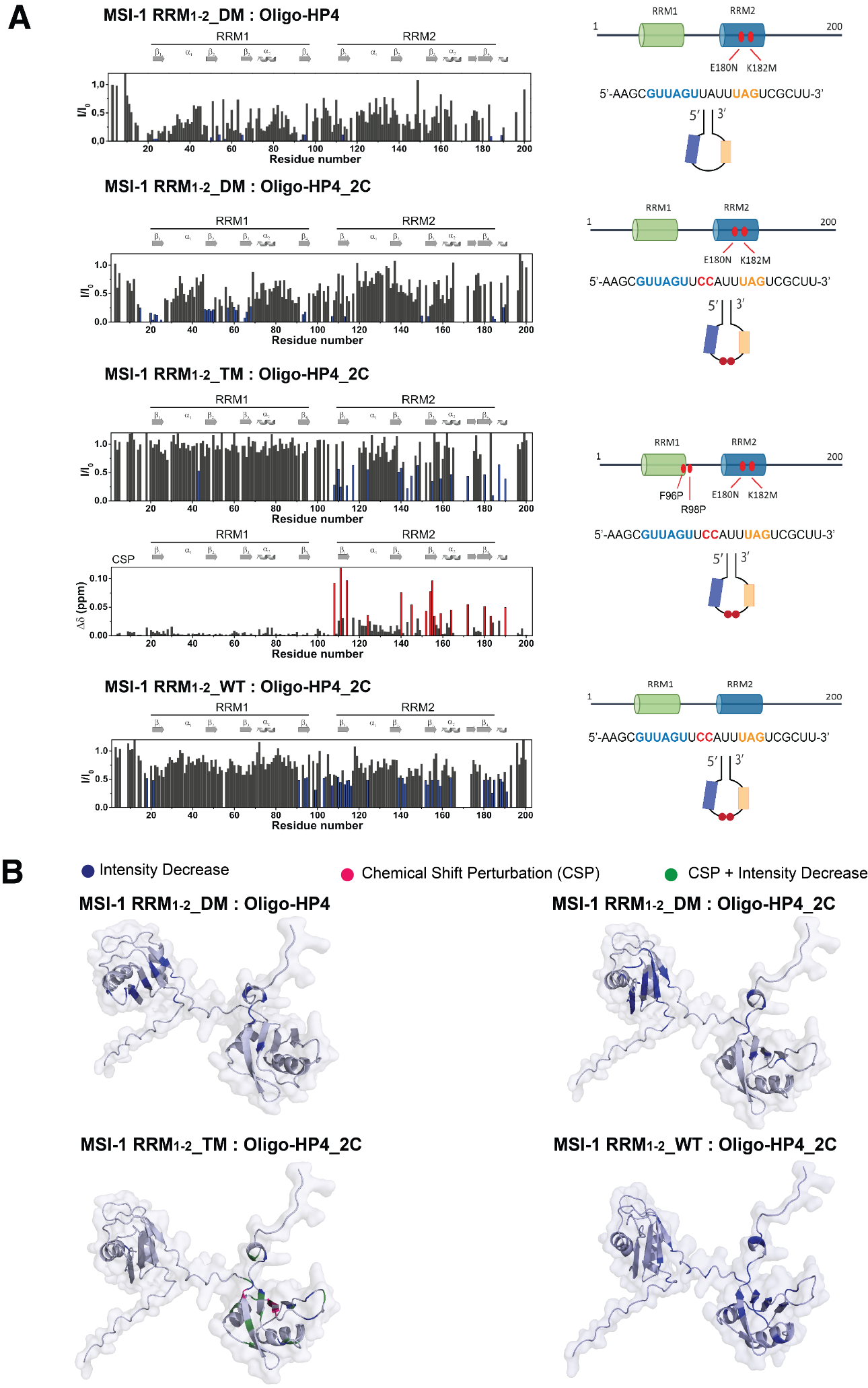


**Figure S18.** Interaction of MSI-1 RRM_1-2_ DM and MSI-1 RRM_1-2_ TM with **oligo-HP4** and/or **oligo-HP4_2C**. (A, top) Plot of the decreases in signal intensity of MSI-1 RRM_1-2_ DM with **oligo-HP4** at a protein: RNA molar ratio of 1:0.25 (shown for comparison purposes). (A, second line) Plot of the decreases in signals intensity of MSI-1 RRM_1-2_ DM with **oligo-HP4_2C**. The residues experiencing the largest changes during the interaction with **oligo-HP4_2C** at the protein: RNA molar ratio of 1:0.25 have been highlighted in blue (Ser-15, Asp-18, Cys-20, Lys-21, Met-22, Phe-23, Ile-24, Gly-25, Gly-26, Val-46, Lys-47, Glu-48, Cys-49, Leu-50, Met-52, Thr-57, Ser-60, Arg-61, Gly-64, Phe-65, Val-66, Phe-68, Lys-93, Val-94, Ala-95, Arg-107, Val-113, Arg-150, Gly-153, Ala-184, Gln-185, Lys.187, Val-189). (A, third and fourth lines) Plot of the decreases in signals intensity and CSPs of MSI-1 RRM_1-2_ TM with **oligo-HP4_2C**. The residues experiencing the largest decreases in signal intensity during the interaction with **oligo-HP_2C** at the protein: RNA molar ratio of 1:0.25 have been highlighted in blue (Phe-43, Thr-108, Lys-110, Ile-111, Gly-114, Ser-117, Asp-124, Met-139, Leu-140, Asp-143, Thr-145, Arg-148, Val-155, Ser-159, Glu-164, His-172, Glu-180, Ala-184, Lys-187, Met-190), while the residues experiencing the largest chemical shift perturbation at a protein: RNA molar ratio of 1:1 have been highlighted in red (Thr-108, Ile-111, Gly-114, Asp-124, Leu-140, Thr-145, Phe-152, Phe-154, Val-155, Thr-156, Ser-159, Glu-164, His-172, Glu-180, Lys-183, Met-190). (A, bottom) Plot of the decreases in signals intensity of MSI-1 RRM_1-2_ WT with **oligo-HP4_2C**. The residues experiencing the largest decreases in signal intensity during the interaction with **oligo-HP_2C** at the protein: RNA molar ratio of 1:0.25 have been highlighted in blue (Asp-18, Lys-21, Asp-91, Val-94, Ala-95, Arg-99, Lys-103, Arg-107, Lys-110, Ile-111, Val-113, Gly-114, Gly-115, Leu-116, Asp-124, Met-139, Met-141, Phe-142, Phe-152, Gly-153, Val-155, Glu-158, Ser-159, Glu-164, Glu-180, Lys-182, Ala-184, Gln-185, Glu-188, Val-189, Ser-191). (B) Mapping of the residues experiencing the largest effect in the tandem domain protein on the structure (AF-O43347-F1) upon binding. Highlighted in blue the residues experiencing the largest intensity decrease, in pink the residues experiencing the largest CSPs, and in green the residues experiencing both effects.


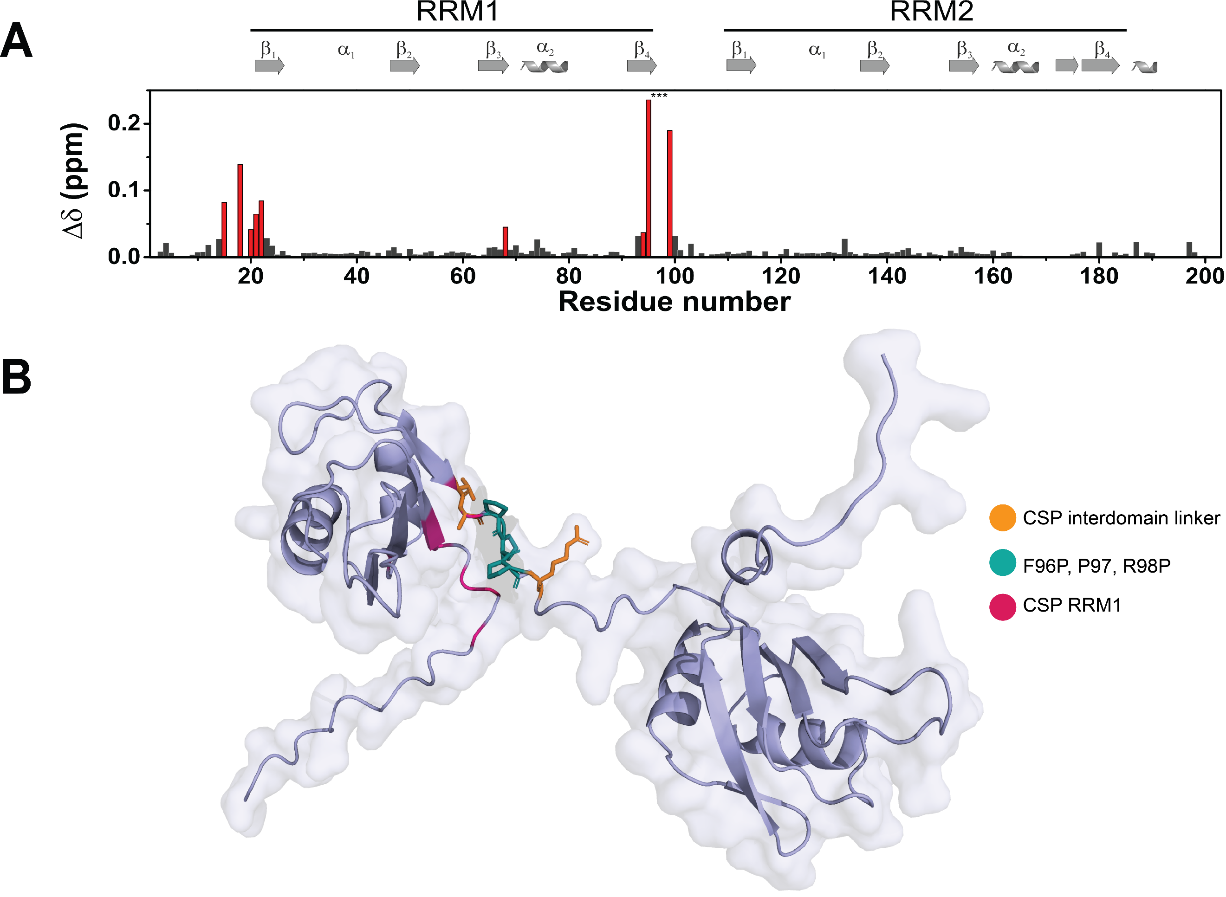


**Figure S19**. (A) Plot of the CSPs of MSI-1 RRM_1-2_ TM with respect to MSI-1 RRM_1-2_ DM. The residues experiencing the largest chemical shift perturbation have been highlighted in red (Ser-15, His-17, Asp-18, Cys-20, Lys-21, Met-22, Phe-68, Val-94, Ala-95, Arg-99). (B) Mapping of the residues experiencing the largest CSP on the structure (AF-O43347-F1). Highlighted in yellow the residues of the interdomain linker experiencing the largest CSP, in green the substitutions and in pink the residues experiencing the largest CSP located on the RRM1 domain.
